# Biogeography of Amazon birds: rivers limit species composition, but not areas of endemism

**DOI:** 10.1038/s41598-017-03098-w

**Published:** 2017-06-07

**Authors:** Ubirajara Oliveira, Marcelo F. Vasconcelos, Adalberto J. Santos

**Affiliations:** 1 Centro de Sensoriamento Remoto, Instituto de Geociências, Universidade Federal de Minas Gerais – UFMG, Av. Antonio Carlos 6627, CEP 31270-901 Belo Horizonte, MG Brazil; 2 Instituto Prístino, Rua Santa Maria Goretti, 86, Barreiro, CEP 30642-020 Belo Horizonte, MG Brazil; 3 Departamento de Zoologia, Instituto de Ciências Biológicas, Universidade Federal de Minas Gerais – UFMG, Av. Antonio Carlos 6627, CEP 31270-901 Belo Horizonte, MG Brazil

## Abstract

Amazonian rivers are usually suggested as dispersal barriers, limiting biogeographic units. This is evident in a widely accepted Areas of Endemism (AoEs) hypothesis proposed for Amazonian birds. We empirically test this hypothesis based on quantitative analyses of species distribution. We compiled a database of bird species and subspecies distribution records, and used this dataset to identify AoEs through three different methods. Our results show that the currently accepted Amazonian AoEs are not consistent with areas identified, which were generally congruent among datasets and methods. Some Amazonian rivers represent limits of AoEs, but these areas are not congruent with those previously proposed. However, spatial variation in species composition is correlated with largest Amazonian rivers. Overall, the previously proposed Amazonian AoEs are not consistent with the evidence from bird distribution. However, the fact that major rivers coincide with breaks in species composition suggest they can act as dispersal barriers, though not necessarily for all bird taxa. This scenario indicates a more complex picture of the Amazonian bird distribution than previously imagined.

## Introduction

The impressive geographic vastness and biological diversity of the Amazon have stimulated attempts of geographic regionalization for more than two centuries. Probably the first proposed subdivision of the Amazonian biota came from A. R. Wallace^1^, who recognised four primate “biogeographic districts” delimited by large rivers. A more recent proposition, based on bird distribution data, emerged as part of the Pleistocene forest refuge hypothesis^2^, which identified four “centers of distribution”. Both hypotheses were based on a general notion that the Amazon’s large rivers could act as dispersal barriers, generating the current distribution patterns of the Amazonian Biota. The most detailed, and accepted proposal for Amazonian biotic regionalization was the areas of endemism (AoEs) firstly proposed for birds by Cracraft^3^. This hypothesis was based on a revision of Amazonian bird distribution, as known at that time, extrapolated to expected distribution range polygons. Since no quantitative methods for identification of AoEs were known back then, Cracraft’s AoEs were delimited among major Amazonian rivers, based on a visual analysis. Thus, as currently understood, the Amazonian Cracraft’s areas of endemism are continuous biogeographic units located between large rivers (the interfluves) that, supposedly, contain a unique, endemic bird fauna.

**Figure 1:**
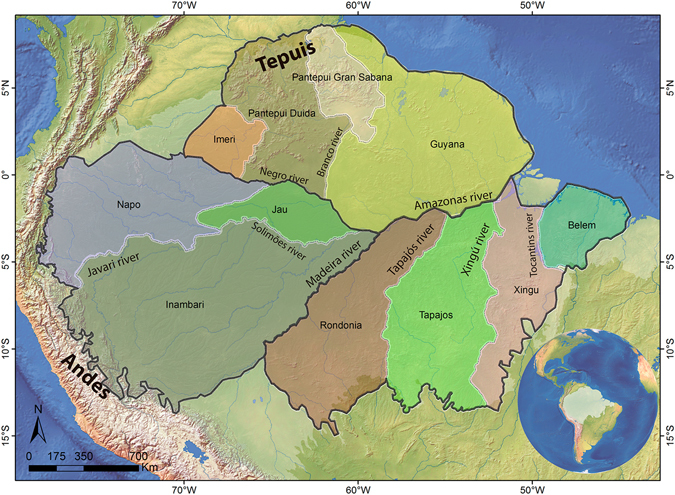
Interfluve hypothesis of Amazonian areas of endemism. The currently accepted Areas of Endemism classification of the Amazon, depicted here, was proposed by Cracraft (1985) and subsequently modified by Silva *et al*. (2002), Naka (2011) and Borges & Silva (2012). Dark lines indicate limits of Wallace (1852) districts. *Map created in ArcGIS 10*.*1* (http://www.esri.com).

Throughout the last three decades, Cracraft’s AoEs have been generally accepted, with only a few modifications. Silva *et al*.^4^ proposed the division of the Cracraft’s *Pará* AoE in the smaller *Tapajós* and *Xingu* AoEs, separated by the Xingu river. Borges & Silva^5^ proposed a subdivision of the *Napo* area with the delimitation of the new *Jaú* area located in the interfluves of Negro and Solimões rivers. Naka^6^, in the first attempt of using quantitative methods for identification of Amazonian bird AoEs, redefined the boundaries of the *Guiana* AoE. The original Cracraft’s AoEs, with those later additions, is what we call herein the interfluve hypothesis (Fig. 1), which, apart from these small additions, have never been tested quantitatively.

The rivers-as-barriers paradigm has also influenced biogeographic studies based on other Amazonian taxa. For instance, Silva & Oren^7^ delimited primate AoEs using Amazonian river interfluves as sampling units, without testing whether the rivers act as barriers. In fact, only a few quantitative studies actually tested this conjecture^8, 9^, with results poorly congruent with the interfluve AoEs. However, these analyses were based on large grid cells, resulting in low accuracy on area delimitation. Thus, it is fair to say that the currently recognized Amazonian AoEs still require quantitative support.

The importance of Amazonian rivers as geographic barriers is supported by several lines of evidence, such as genetic differentiation between populations^10, 11^, and distribution patterns of passerine birds^12, 13^. However, these studies are restricted to a few rivers and taxa. Furthermore, rivers do not appear to be a major barrier for all taxa^12, 14^, suggesting that the Amazonian biota is the product of more complex evolutionary processes^15, 16^.

The situation described above did not prevent the wide use of the interfluve AoEs as a premise in biogeographic studies, such as area relationship inference^17–20^, local species survey^21^ and population genetic analyses^22^. This is particularly problematic considering the growth of knowledge on Amazonian avifauna during the last 30 years, including new species descriptions^23^ and range expansions of many taxa^24, 25^, which do not support the interfluve AoEs.

In this study we test whether the limits of the Interfluve AoEs (Fig. 1) can be recovered through quantitative analysis of bird distribution data, based on three methods of AoEs delimitation and through GIS and statistical methods applied to spatial variation in species and subspecies composition. The interfluve hypothesis, by delineating AoEs, can be tested with quantitative methods^26–30^. Thus, we used methods that assume AoEs as areas limited by species co-occurrence^26, 29, 31^, which is the widely accepted concept of AoEs^30, 32–35^. Different from Cracraft^3^, our analyses are not based on estimated distribution polygons, but on point occurrence data of Amazonian birds taken from the literature and scientific collections. Thus, this is the first study to quantitatively evaluate bird distribution patterns in the Amazon, and the first empirical test of the widely accepted interfluve AoEs hypothesis.

## Results

### Identification of areas of endemism

Our tests of the interfluve hypothesis were based on two datasets, one composed by distribution records of 612 Amazonian bird subspecies and another in which subspecies were merged within species, comprising records of 566 species. We built these two databases because the interfluve hypothesis was originally based on bird subspecies data. However, since bird subspecies delimitation can be controversial and are often based on geographic barriers, we decided to also analyse data classified only at species level. We delimited AoEs through three approaches that use different logical basis to identify co-occurrence patterns of species, in a way to perform a rigorous test of the interfluve AoEs hypothesis. The Geographical Interpolation of Endemism (GIE) interpolates species distribution through a kernel density function^29^ to estimate the degree of overlap in the species ranges in a spatially explicit way. The EnDemisM analysis (NDM) is based on spatial optimization of shared species between grid cells based on an endemism index, resulting in an estimate of species distribution overlap^36^. Finally, the Parsimony Analysis of Endemicity (PAE), identifies AoEs based on a cladistic analysis of grid cells, with species as characters, to identify clusters of grid cells that are interpreted as AoEs^26^.

**Figure 2:**
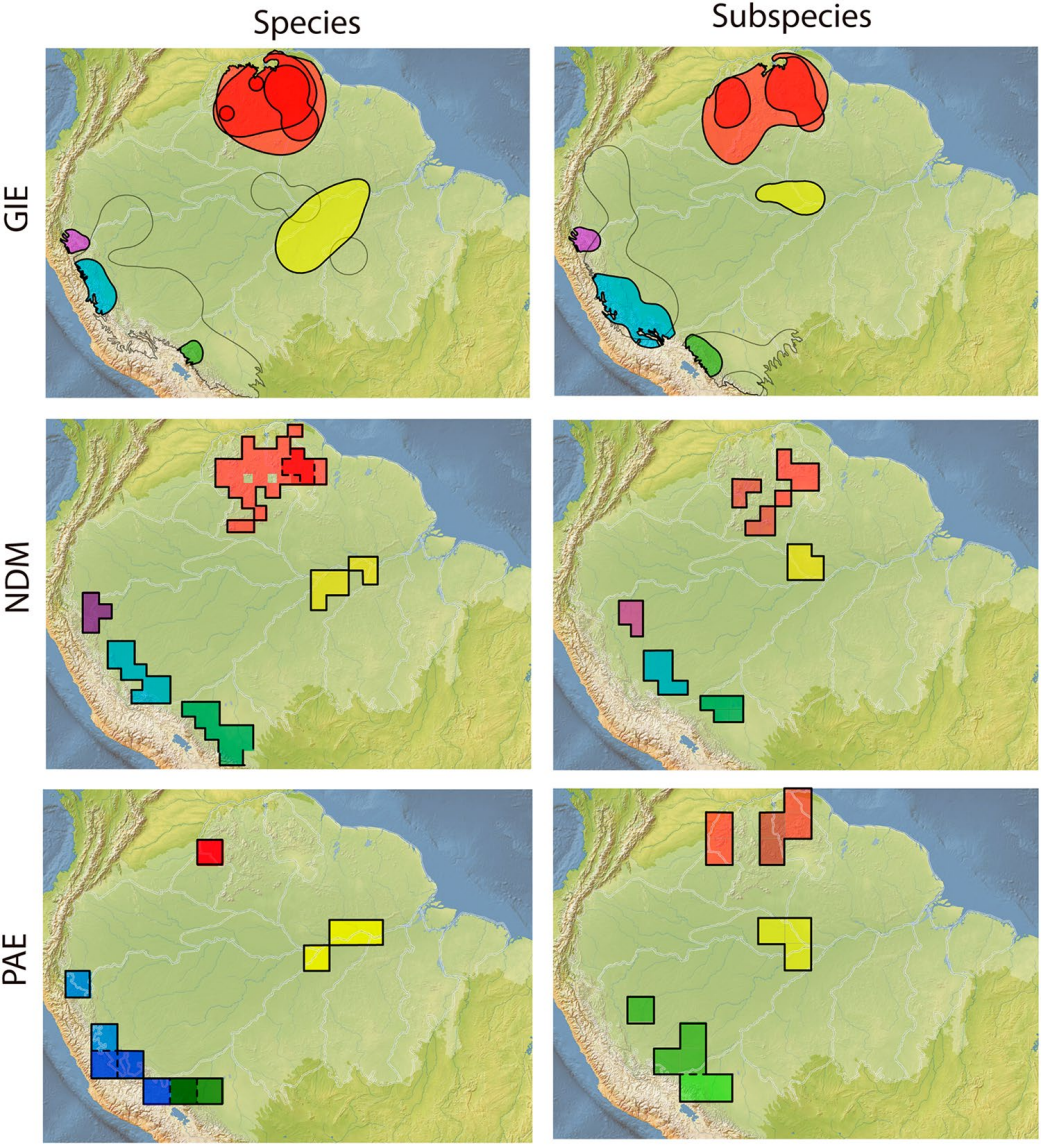
Amazonian bird areas of endemism. Areas were identified using three methods (GIE, NDM and PAE), based on species and subspecies datasets. Same colours indicate areas with high overlap in all analyses. Dashed lines indicate the boundaries of interfluve hypothesis of Amazonian areas of endemism, as in Fig. 1. *Maps created in ArcGIS 10*.*1* (http://www.esri.com).

The three AoEs delimitation methods, applied to each of the two datasets, resulted in geographic units in similar locations, though different in the number and size of AoEs. Most importantly, no analysis recovered the Interfluve AoEs. The species-based GIE identified 46 AoEs (Fig. 2, Appendix S1: Fig. 5), each supported by up to 20 synendemic species. Ten AoEs were identified within a larger area on the Andean foothills (Fig. 2, in blue, green and purple). The Tepuis region shows a large AoE (Fig. 2, red area), containing two other smaller AoEs which encompass portions of the Amazonian highlands and savanna formations. Finally, an AoE was identified in the Solimões/Negro and Tapajós/Madeira/Amazonas interfluves, as well as on the southeastern border of the Amazon (Fig. 2, yellow area). The subspecies-based GIE identified 51 AoEs (Fig. 2, Appendix S1: Fig. 7), each supported by up to 25 synendemic species. These results were relatively similar to those obtained in the species-based analyses (r = 0.54, p = 0.001). Since sampling effort could influence the identification of AoEs, we estimated the density of bird distribution records throughout the study area using a kernel interpolation method. This procedure converted the spatial variation in the number of bird occurrence records into a density surface map (Appendix S1: Fig. 2). The distribution record density was expressed in this map through the resulting kernel index, which was compared through pixel-to-pixel, Pearson correlation analysis with the kernel index of the GIE AoEs. This analysis showed a high variation of sampling effort throughout the Amazon, with several peaks coinciding with the GIE’s AoEs (Appendix S1: Fig. 2). However, several highly sampled areas are located outside AoEs, and the estimated distribution of sampling effort showed low correlation with the GIE results for both species and subspecies (r = 0.16 and 0.24, respectively, p = 001).

The species-based NDM identified nine consensus AoEs (Fig. 2, Appendix S1: Fig. 9), two in the Tepuis, six in the Andean foothills and one in the Madeira/Tapajós interfluve. The subspecies NDM identified 40 consensus AoEs (Fig. 2, Appendix S1: Fig. 10), 20 in the Tepuis, 17 on the Andean foothills and one in Solimões/Negro interfluve.

The species-based PAE resulted in 252 equally parsimonious trees with 44 AoEs, most composed by spatially non-adjacent cells (Fig. 2, Appendix S1: Figs 11 and 12). Four AoEs were composed by adjacent cells at the base of the Andes and in the Madeira/Tapajós interfluve, the latter included within a larger area formed by disjoint grid cells delimited by Madeira and Xingu rivers. The subspecies-based PAE resulted in 3,992 equally parsimonious trees (Appendix S1: Figs 14 and 15), most composed by non-adjacent cells. Twenty four AoEs were composed by adjacent cells, at the base of the Andes, one in the Solimões/Madeira/Tapajós interfluve and another at the Tepuis. The constraint analysis with either species or subspecies data resulted in longer trees (respectively 524 and 178 additional steps), without synendemic species in any AoE (Appendix S1: Figs 13 and 16).

The overlap between the AoEs identified here and the interfluve AoEs was usually below 60%. A moderate spatial congruence between our results and the interfluve AoEs was observed with *Jaú*, though with less than 50% overlap, and with *Rondônia*, which was partially delimited in the species-based analyses (yellow area in Fig. 2).

### Species co-occurrence and fit to AoE

It is expected that AoEs show high fit between its limits and the limits of distribution ranges of its synendemic species^35^. To test this prediction for the interfluve hypothesis, we create an index that expresses the fit between species distribution and limits of AoEs in a 0 to 1 scale (1 = total fit). The fit between species distribution ranges and the interfluve AoEs were usually low, both in species dataset (mean 0.50) and subspecies dataset (0.70) analyses (Appendix S2). The highest fit values were obtained for *Imeri*, *Jaú* and *Pantepui Gran Sabana* AoEs (0.70) in subspecies analysis. In the species analysis, 172 species were fully inserted within the limits of interfluve AoEs, but these species usually occupy at most 10% of each area. Additionally, *Belém* and *Xingu* showed no exclusive species. In subspecies analysis 197 subspecies were fully inserted within the limits of interfluve AoEs, four of these subspecies occupy 100% of *Pantepui Gran Sabana* AoE.

### Breaks in species composition

To test whether major Amazonian rivers act as distribution limits for birds, we identified major breaks in species composition using the Monmonier’s Algorithm, which identify most significant breaks in the spatial distribution of a dataset^37^. The spatial variation in species composition was expressed in a cell-to-cell matrix using the Bray-Curtis dissimilarity index. Most barriers identified through this procedure were coincident with the Branco, Solimões and Amazonas rivers on the analyses of the two datasets (Appendix S1: Fig. 18). Additional barriers were identified at the base of the Andes and on the Tepuis, which is consistent with the AoEs delimited in this study.

**Figure 3:**
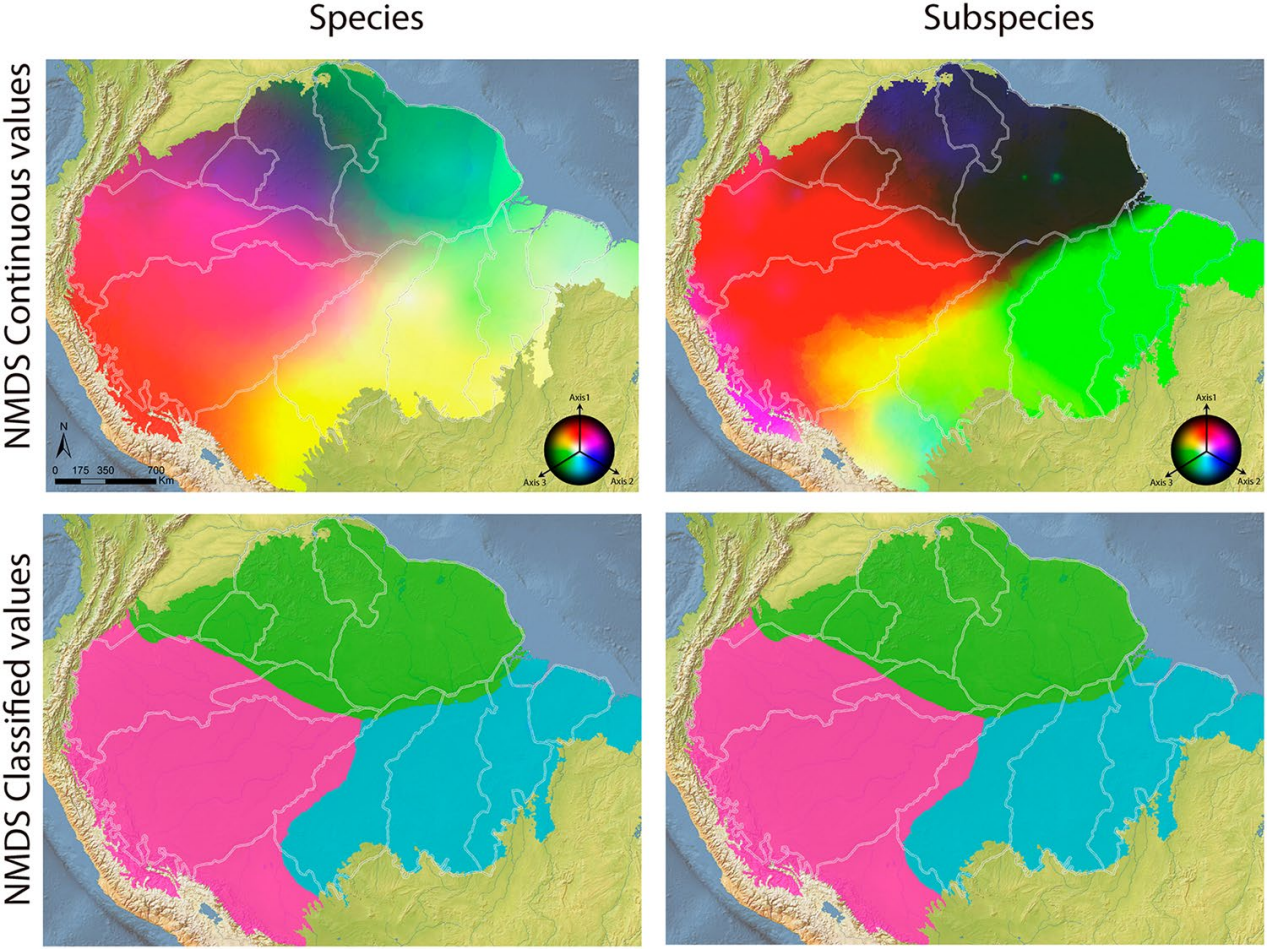
Spatial variation in bird species composition throughout the Amazon. Species composition were obtained by the interpolation of NMDS scores (three axis represented by a RGB scale) and the first three breaks (most significant in search order) in species composition identified by the Maximum Likelihood classification, both based on species and subspecies datasets. *Maps created in ArcGIS 10*.*1* (http://www.esri.com).

**Figure 4:**
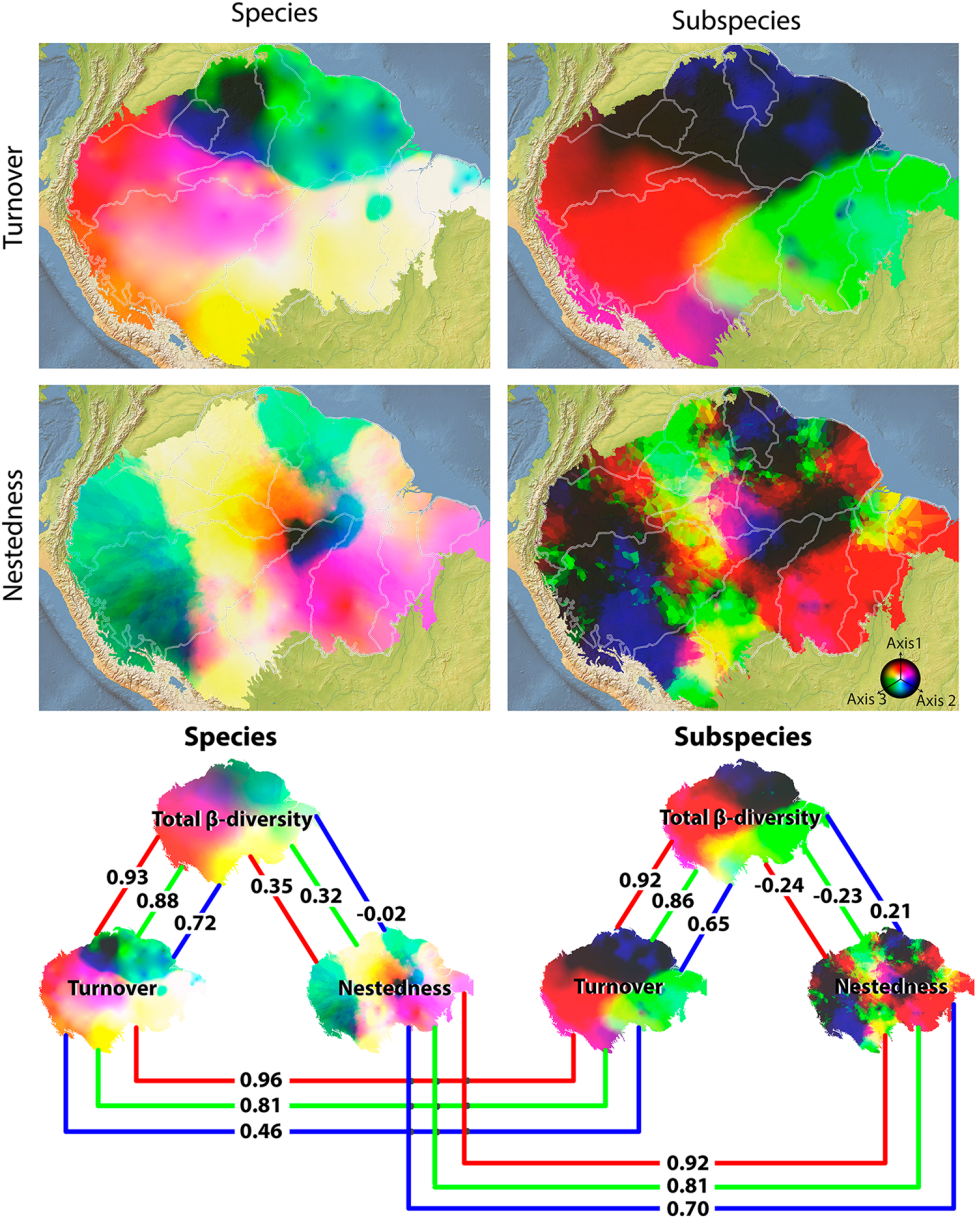
Partitions of spatial variation of bird species composition throughout the Amazon. Species composition was obtained by interpolation of NMDS scores (three axis represented by a RGB scale). Beta-diversity is partitioned into turnover and nestedness for species and subspecies. Lines indicate each axis and correlation between them. *Maps created in ArcGIS 10*.*1* (http://www.esri.com).

We described the spatial variation in species/subspecies composition using the Bray-Curtis dissimilarity index mentioned above, transformed into vector values through Non-metric Multidimensional Scaling (NMDS). The vector values were then interpolated through a Bayesian kriging technique^38^ to generate a surface map of variation of species composition (Figs 3 and 4). To identify the components that generate bird beta-diversity variation throughout the Amazon, we partitioned beta-diversity values into turnover and nestedness components^39^. The results of the partition analyses were expressed as surface maps. The NMDS analysis showed a high correlation between the observed distance and the distance of ordination, both when analysed from species (non-metric fit R^2^ = 0.96, linear fit R^2^ = 0.79), subspecies datasets (0.97, 0.84) and partition of beta-diversity: species turnover (0.95, 0.59) and nestedness (0.96, 0.76), and subspecies turnover (0.96, 0.74) and nestedness (0.94, 0.55), indicating that the analyses satisfactorily represented the Bray-Curtis distance matrices. All NMDS axes showed high and significant values of spatial autocorrelation (Appendix S1: Fig. 17), satisfying the premise of empirical Bayesian Kriging interpolation. The scores of the tests based on species and subspecies are quite similar, with high correlation with the three main axes (Appendix S1: Fig. 19). The first axis indicated a division between the southern and northern lands around the Amazonas and Solimões rivers (Appendix S1: Fig. 19), suggesting that these rivers coincide with distribution range limits for most bird species. In subspecies analysis the first axis indicated an east/west division around the Tapajós and Negro rivers. The most apparent division according to the second axis in species analysis coincides with the Madeira River and approximately with the Branco River. In subspecies analysis the division coincides with Madeira and Amazonas. The third axis showed a higher disagreement between the datasets, indicating higher composition dissimilarity between western and northern versus central Amazonia on the species dataset (Appendix S1: Fig. 19). In subspecies dataset the third axis coincides with highlands of Amazonia, as the Tepuis and the base of the Andes. The three axes combined identified major changes in species composition around the limits of the *Guiana* interfluve AoE, and the Negro and the Madeira rivers, though with conflict between species and subspecies datasets (Fig. 3). Additionally, differences in species composition were observed among the *Rondônia*, *Tapajós* and *Xingu* interfluve AoEs, but the transition between them were mostly continuous, and not steep as would be expected if the rivers act as strong dispersal barriers. In fact, the strongest breaks in species composition detected within these AoEs do not coincide with their proposed limits (Fig. 3). The variation in species/subspecies composition seems more related to the taxa turnover than to nestedness (see correlations in Fig. 4). The turnover maps resemble the general pattern of species/subspecies composition more strongly than nestedness maps (Fig. 4). To identify the main species composition breaks, we used an unsupervised Maximum Likelihood classification, which groups areas based on their similarity. In this analysis, species composition showed low concordance between species and subspecies datasets, but neither identified interfluve AoEs, with the exception of *Napo* (partially, in all datasets) (Appendix S1: Fig. 20). As another way of testing the congruence between the boundaries of the interfluve AoEs and species composition breaks, we tested whether the data could support the interfluve AoEs through a Discriminant Analysis of the NMDS scores None of the interfluve AoEs were identified in the Discriminant Analysis, which showed a low hit percentage by the discriminant model. Only a few areas in the subspecies analysis showed high values of accuracy (Appendix S1: Table 22).

## Discussion

Our analyses showed little support for the interfluve AoEs. However, our results support the role of some major Amazonian rivers as breaks in species composition. Although the methods used in this study to identify AoEs have different logical properties, they provided similar results even applied to either species or subspecies datasets. This means that differences in taxonomic treatment of the data, either using subspecies or species-level classification, does not explain the conflict between our AoEs delimitation analyses and the AoEs delimited in the interfluve hypothesis. Finally, we demonstrated that bird distribution patterns in Amazonia are more complex than previously envisioned.

The incongruity between our results and the interfluve hypothesis may be related to the accumulation of new data since Cracraft^3^. For example, the Yapacana Antbird *Myrmeciza disjuncta* and the Peruvian Recurvebill *Syndactyla ucayalae* were considered restricted to one AoE in Cracraft^3^, but recently had their distribution ranges expanded to different interfluves^40, 41^. In addition, some recently described species have distribution records inconsistent with the interfluve hypothesis (e.g. the Western-Strolated Puffbird *Nystalus obamai* ^23^). The spatial variation of distribution record density (Appendix S1: Fig. 2) show that most of the Amazon are still undersampled. However, the low correlation between AoEs and distribution record density shows that our results cannot be explained solely by variation in sampling effort. In fact, poorly sampled areas may represent unknown AoEs, but those that have been identified here are supported by strong empirical evidence, and several areas outside GIE’s AoEs also have high distribution record density. Finally, it is worth mentioning that even with sampling effort deficiencies, the datasets analysed here are more complete than those used for the original formulation of the interfluve hypothesis^3^.

In this study, we evaluated the interfluve hypothesis through a test of a basic prediction of any AoEs hypothesis, which is a high congruence between the limits of synendemic species distribution and the limits of the area of endemism^35^. The species-to-AoE fit analyses showed that some interfluve AoEs have few endemic species/subspecies or have endemic species occupying a very small portion of its area. For instance, species that have been indicated by Cracraft^3^ as restricted to specific AoEs, such as the Cayenne Nightjar *Setopagis maculosa*, White-throated Pewee *Contopus albogularis*, Tinamou *Crypturellus ptaritepui*, White-faced Whitestart *Myioborus albifacies*, the Guaiquinima Whitestart *M*. *cardonai*, *and* the Chestnut-headed Nunlet *Nonnula amaurocephala*, occupy less than 30% of specific interfluves (Appendix S2). Thus, these species could at most support small AoEs inside interfluves, but do not allow the delimitation of the whole interfluves as single AoEs, and neither indicates the rivers were responsible for generating AoEs. Unless one extrapolates the distribution ranges of each species up to the nearest major rivers, necessarily considering them as the limits of species ranges (as done in Cracraft^3^), it would not be possible to identify the limits of AoEs as those proposed in the interfluve hypothesis. However, this extrapolation would lead to a tautological argument, since the rivers as AoEs’ limits is exactly what has been evaluated in this study.

Another prediction of the interfluve AoEs hypothesis is that most species should be endemic to a specific interfluve. However, about 90% of the subspecies and 80% of the species analysed herein occur in more than one interfluve (Appendix S2). Additionally, more than half of the species and subspecies also occur in more than five interfluve AoEs. This demonstrates that, even though rivers can limit the distribution of Amazonian bird species or subspecies, they do not do so in such a way that species ranges are usually restricted to a single interfluve, as will be discussed in detail below.

**Figure 5:**
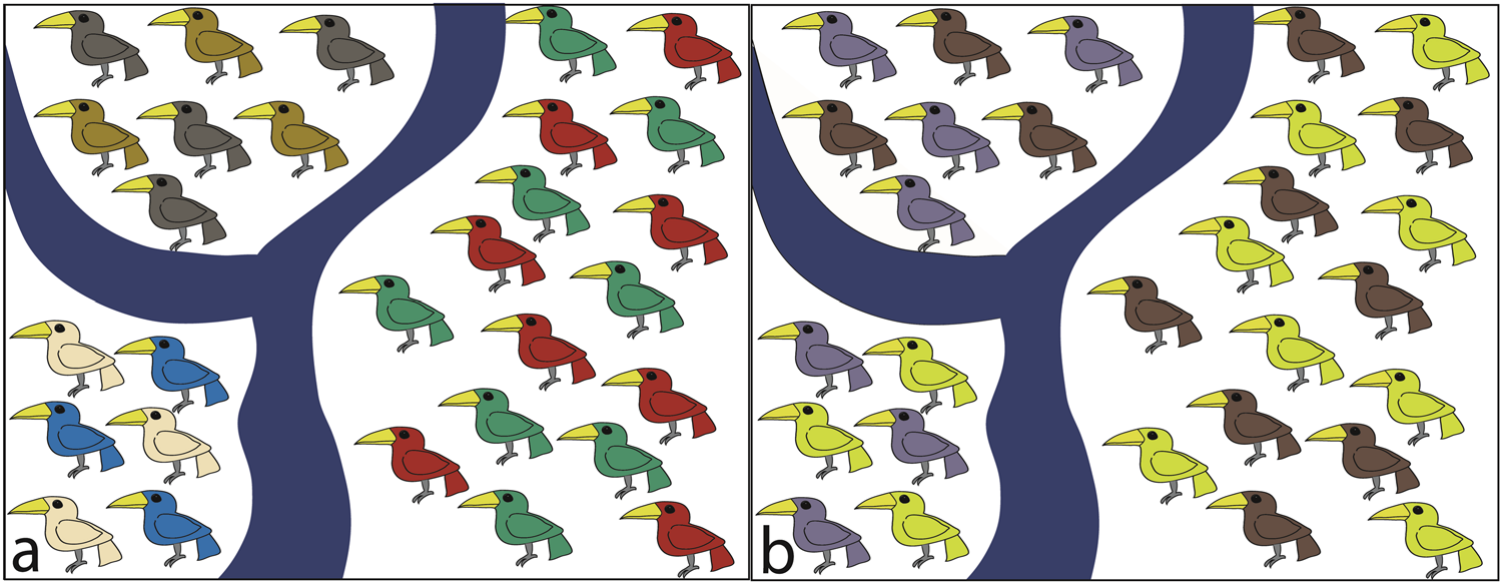
Hypothetical examples illustrating the role of rivers as limits of areas of endemism and as breaks of species composition. As discussed in this study, in (**a**) bird species distribution are confined by rivers, generating areas of endemism in each interfluve. In (**b**), bird species respond differently to each river as a dispersal barrier. Thus, the rivers mark steep changes in species composition, but do not limit areas of endemism.

Although our results do not corroborate that large Amazonian rivers act necessarily as borders of AoEs, there is evidence that they can limit species distribution ranges. To act as AoEs limits, rivers should limit the distribution of a set of species, and these species should occur across the interfluve (Fig. 5a), something we did not observe. Bird species differ in dispersal ability, which is mostly related to species habitat or lineage age, generating idiosyncratic responses of species distribution to rivers as barriers^12, 42^. Thus a given river could be a dispersal barrier to some species, but not to others (Fig. 5b). Additionally, for each interfluve to correspond to an AoE, its endemic species should occupy all or most of its extension. For instance, we have found an AoE within the Cracraft’s *Rondônia*, limited by the Amazonas, Madeira and Tapajós rivers (yellow in Fig. 2). However, apparently other factors pose a southern limit to the distribution of species in this area, restricting this AoE to the northern portion of the interfluve. Of course, we must consider that the distribution ranges of some species may be larger than what we know today, due to lack of sampling (Appendix S1: Fig. 2). However, this cannot be used as evidence in favour of the interfluve hypothesis, since we do not have evidence of the occurrence of these species throughout the entire interfluve. One can imagine that a species with high dispersal capacity, which would occupy the whole interfluve, would also be capable of crossing the rivers at least in their narrower, upriver portions. In fact, population genetics evidence suggests that dispersal of Amazonian birds over major rivers is dependent on the width of the watercourse^43^, and our results suggest that the largest Amazonian rivers are probably the most effective dispersal barriers, since they marked the major changes in species composition (Fig. 3).

Our results indicate that the greatest changes in bird species/subspecies composition in Amazonia partially coincide with the limits of AoEs and other biogeographic units previously proposed^1–3, 6, 44^. These composition changes seem to be more associated with species turnover than nestedness. Among the major rivers, the Amazonas/Solimões and Madeira emerged as potentially important biogeographic barriers, as demonstrated in the Monmonier’s and NMDS analyses (Appendix S1: Fig. 18). The importance of these rivers as biogeographic barriers has been already postulated^1–3^ and demonstrated empirically for passerine birds at least along the Amazonas^12^. In fact, the Maximum Likelihood classification of species composition was very similar to Wallace’s^1^ districts, which emphasize the importance of these rivers in the Amazon biogeography. However, other rivers such as the Tapajós and Xingu, do not seem to represent barriers along its entire length, as shown in our analyses and in phylogeographic studies^45^. Thus, our results have shown that, although rivers can limit distribution ranges of Amazonian birds, this does not mean that bird species should have similar distribution, generating interfluve AoEs. For instance, two taxa limited by the same river may have poorly overlapped distribution ranges, occurring in different interfluves and crossing different rivers, generating species composition breaks, but not an AoE (Fig. 5b).

Despite the importance of rivers as geographic barriers in Amazonia, other physical factors may be responsible for the regionalization of the Amazonian biota. The quantitative analyses presented here, based on two datasets, agree on the presence of AoEs on the Andean hillside and the Tepuis (Fig. 2). This suggests that orographic processes seem to be more important than rivers in the delimitation of Amazonian AoEs. Furthermore, the altitudinal variation also appears to exert a strong influence on species composition on those regions. Other studies have shown marked differences in species composition^46–48^, and phylogeographic structuring^16, 49–51^ in these regions, compared to the Amazon basin. This is possibly related to environmental differentiation due to altitude. For instance, Andean hillside forests are influenced by their distance from the Atlantic and the Andean cordillera, which prevents the arrival of the humid currents from the Pacific^52^. These climatic differences, together with historical factors on the Andean hillside and the Tepuis, are related to differences in species composition^53, 54^. Thus, based on our results, we suggest that rivers are not the only, nor necessarily the main factors, generating bird AoEs and species composition variation in the Amazon.

In conclusion, our quantitative analyses of species or subspecies distribution data did not corroborate the interfluve hypothesis of AoEs for Amazonian birds. However, this does not discard the importance of Amazonian rivers as biogeographic barriers, as demonstrated both in this study and in others. If some groups with low dispersal ability, as the species of the Trumpeter genus *Psophia*, are limited by all major Amazonian rivers^55^, many other species can apparently cross some rivers^42^. This is dependent not only on biological attributes of the species, but also on the width variation along the watercourse^42, 43^. Thus, the rivers can generate species composition patterns (Fig. 5b), without necessarily generating AoEs. The results of this study suggest that other physical factors, such as variation in the structure of vegetation and altitude, can be more important than the hydrography in the regionalisation of the Amazonian biota. This highlights that the evolution of Amazonian biota is more complex than previously imagined, requiring the use of different analytical tools and multiple sources of data for its understanding. Our results have also shown that the central Amazon is particularly complex, since the analyses with different methods, based either on species or subspecies data, generated inconsistent results over this region. This part of the Amazon is probably the one that will require more sampling effort (Appendix S1: Fig. 2) and analyses in the future.

## Methods

This study is restricted to the Tropical & Subtropical Moist Broadleaf Forests ecoregion (http://www.worldwildlife.org/pages/conservation-science-data-and-tools), including islands of Tropical & Subtropical Grasslands, Savannas & Shrub lands contained within the limits of the Amazon. We built a database of bird distribution records through GBIF (http://www.gbif.org), SpeciesLink (http://www.splink.org.br), Brazilian scientific collections, and recently published species lists (Appendix S1: Table 3). These data include major collections with a large representation of Amazonian bird records (Appendix S1: Table 4). Our database includes 54,187 records for 566 species and 612 subspecies, totalling 1,099 taxonomic entities, all endemic to the Amazon according to Natureserve polygons and distribution information available in AVIBASE (http://avibase.bsc-eoc.org) and Handbook of the Birds of the World (http://www.hbw.com). We checked whether coordinates originally provided for each distribution point match their localities through comparison with vector layers of the state, conservation units and municipality boundaries of Amazonian countries using ArcGIS. As a result, 61% of occurrence points were considered correctly georeferenced, and remaining records were georeferenced through gazetteers and online databases (http://www.splink.org.br; http://mapas.ibge.gov.br). The distribution of all species was manually checked by visual comparison to estimated bird distribution in the Handbook of the Birds of the World, and the few disparate records found were excluded. Species taxonomy follows the South American Classification Committee (2014) (http://www.museum.lsu.edu/~Remsen/SACCBaseline.htm).

### Species dataset

This dataset included distribution data at the species level, ignoring subspecies classification. Most studies on Amazon birds are based on subspecies distribution, supposedly because, as more recently diverged lineages they could provide a more detailed picture of recent biogeographic processes. However, bird subspecies delimitation is particularly prone to disagreement between authors and taxonomic catalogues, which hampers dataset compilation. Additionally, because most Amazonian bird subspecies were delimited based chiefly on populations separated by large rivers, including them in our analyses could generate a circularity effect on the results. Thus, we built a conservative, though taxonomically coarse dataset with 54,187 records of species-level taxonomic entities, with subspecies merged under each species.

### Subspecies dataset

This dataset was built to facilitate comparison with previous studies, and to evaluate the impact of database taxonomic level over our conclusions. Taxonomic catalogues and data sources strongly disagree on subspecies limits and distribution, and most collection records are not identified at subspecies level. Thus, we used the known bird distribution according to the Handbook of the Birds of the World as a basis to split records identified only at species level into subspecies records. This was possible because Amazonian bird subspecies are usually allopatric, with rare exceptions that were not included in our analyses. We adopted subspecies distribution data even in the cases subspecies distinction was based on populations separated by major rivers, though it could bias the results in favour of the interfluve AoEs. The database included 54,187 records of 612 subspecies.

### Identification of areas of endemism

To identify AoEs in GIE, species are divided into different range size categories to optimize the overlap analysis, since species with very different ranges do not have a high degree of sympatry. The size of the categories does not change significantly the results (see details in Oliveira *et al*.^29^). Thus, we split data in 11 categories, according to the distance between the centroid of the species/subspecies distribution and their farthest occurrence point: up to 100 km, 101–200, 201–400, 401–600, 601–800, 801–1000, 1001–1200, 1201–1400, 1401–1600, 1601–2000, and between 2001 and 2150. To generate the consensus AoEs, the kernel index of each category were standardized between 0 and 1 before assembling the maps. The results of GIE analysis for species and subspecies datasets were compared through map correlation.

The NDM and PAE are based on grid cell data, and their results are sensitive to cell size. Thus, we tested several cell sizes (0.5° to 2°) and used the size that allowed the identification of more AoEs. The NDM analyses were implemented in VNDM^56^ on a one-degree grid map. Search analyses were set to retain areas with scores equal to or above 1.0 and with one or more endemic species. The search was repeated 100 times, keeping overlapping areas only if 90% of the species in each one are unique. Only grids with actual species records were included in the analysis. The results included areas that share at least 80% of their endemic species through the “consensus flexible AoE” criterion^57^.

The PAE was based on a presence/absence matrix of bird species over a two-degree grid. The matrix was analysed through TNT 1.1^58^, using search procedures designed for analysis of large data matrices^59^. The tree-search started with 1,000 random-addition sequence trees, which were submitted to TBR Branch Swapping, retaining 99 trees per replicate, followed by a sequence of 50 cycles of sectorial search through the entire trees (with sectors below 75 taxa analysed through RAS + TBR); tree-drifting with 200 iterations; ratchet with 500 iterations; and 100 rounds of tree-fusing. The shortest trees obtained were submitted to two additional rounds of TBR to assure global optimum was found. The AoEs were delimited from clades unambiguously supported by at least one non-homoplastic species occurrence, identified in the strict consensus tree. To compare the results to the interfluve hypothesis, the analysis was repeated constraining grid cells to interfluve AoEs.

In order to verify the effect of the sampling effort on the identification of the areas of endemism we estimated the density of distribution records through a kernel interpolation. We performed correlation analysis with corrected degrees of freedom^60^ between the kernel index of distribution records (used as an index of sampling effort) and the kernel index of the GIE, based on species and subspecies datasets. We used the GIE results because it presents continuous values that can be used in the correlation analysis. In addition, our results with the other methods were very congruent to those obtained in the GIE analysis.

### Species fit to AoE

It is expected that AoEs show high fit between its limits and the limits of distribution ranges of its synendemic species^35^. To test this prediction for the interfluve hypothesis, we quantified the percentage of area overlap between species distribution and interfluve AoEs on one-degree grid maps. We measured species-to-AoE fit through an index calculated by:

$${\bf{S}}{\bf{p}}{\bf{e}}{\bf{c}}{\bf{i}}{\bf{e}}{\bf{s}}\,-\,{\bf{t}}{\bf{o}}\,-{\bf{A}}{\bf{o}}{\bf{E}}\,{\bf{f}}{\bf{i}}{\bf{t}}=\frac{a+b}{c}$$

- percentage of species distribution areas contained within the AoE
- percentage of the AoE’s area in which the species occurs
- sum of the maximum values of each percentage (=200)

High values of this index indicate that the species is very restricted to AoE and occupies much of its area. To obtain the Species-to-AoE index we compute the average of the indices of species within each AoE. In this analysis we considered only the species that had more than 90% of their distribution contained within the AoE. We chose this arbitrary threshold only as a way to exclude species that are not restricted to specific AoEs.

### Breaks in species composition

To test whether major Amazonian rivers act as distribution limits for birds, we identified major breaks in species composition using the Monmonier’s Algorithm^37^ based on a Bray-Curtis dissimilarity matrix. We identified the top 15 barriers using a network connection based on Thiessen polygons and Delaunay triangulation. This analysis was performed in the R software (www.r-project.org) packages adegenet (http://adegenet.r-forge.r-project.org) and vegan (http://vegan.r-forge.r-project.org).

We also described the spatial variation in species/subspecies composition using statistical ordination. Spatial variation in species composition is usually mapped through Generalized Dissimilarity Modelling (GDM)^61^, which assumes a correlation between environmental variables and species composition. Since we prefer not to rely on that premise, we implemented an analogous analysis. This analysis consists on the use of a Bray-Curtis dissimilarity matrix transformed into linear values through Non-metric Multidimensional Scaling (NMDS) of species distribution, which are then interpolated through a Bayesian technique on a map. This procedure of interpolation assumes only that the interpolated values are spatially autocorrelated. Thus, values of non-sampled sites are estimated as intermediate to values of nearby, sampled sites, proportionally to the distance between the points. The analysis was performed through the following steps (Appendix 1: Fig. 1):

1. A species presence/absence matrix was assembled from one-degree grid maps.
2. A cell-to-cell Bray-Curtis dissimilarity matrix was calculated.
3. This matrix was analysed through NMDS with a hundred random starts used to find the lowest stress values and the minimum number of axes which satisfactorily represent the dissimilarity matrix (in our case three axes).
4. The NMDS scores (for each axis) were plotted on the centroid of each grid cell.
5. These points were interpolated using an empirical Bayesian Kriging technique, which considers that intermediate values must occur proportionally to the distance between points in a normal distribution, describing a smooth curve^38^. Thus, we obtained three surface maps (one for each axis) with interpolated NMDS scores.
6. The maps were summarized on a RGB map, with a different colour representing each NMDS axis.

To test the premise of spatial autocorrelation, we calculate the Moran’s I for each axis of the NMDS. Grid cells with less than ten records were excluded from the analysis, since they could inflate Bray-Curtis dissimilarity, influencing the NMDS results. The similarity between the results of species and subspecies-based analyses was measured through a Pearson correlation analysis for each NMDS axis. The agreement between species composition maps and interfluve AoEs was tested through a general Discriminant Analysis model of NMDS scores taken at 100 random points within each interfluve AoE in the R software. Furthermore, we conducted an unsupervised classification of the species composition map, testing two to 11 groups by Maximum Likelihood. The NMDS analysis was performed in the R package vegan, and Bayesian Kriging interpolation and Maximum Likelihood classification in ArcGIS.

To investigate which beta-diversity partition would be influential on the variation of species composition, we performed the same analyses described above for turnover and nestedness partitions. To do this, we calculated the matrices of each partition in the R package “betapart”^39^ and performed steps 3 to 6 described above.

## Electronic supplementary material

Appendix S1

Appendix s2
