# Supplementary material for: Biogeography of Amazon birds: rivers limit species composition, but not areas of endemism
Source: Sci Rep. 2017 Jun 7;7:2992. doi: 10.1038/s41598-017-03098-w (PMC5462822; doi:10.1038/s41598-017-03098-w)
Supplement: Supplementary file 1 — Appendix S1 [file 41598_2017_3098_MOESM1_ESM.pdf]

SUPPORTING INFORMATION

Biogeography of Amazon birds: rivers limit species composition, but not areas of endemism

Ubirajara Oliveira, Marcelo F. Vasconcelos and Adalberto J. Santos

Appendix S1: Additional references, institutions that provided distribution records and results of AoEs and Detailed results of species composition analyses.

1 - Step-by-step analysis of spatial variation in species composition. See the Material and methods for details. Map created in ArcGIS 10.1 (http://www.esri.com)..................................3

2 - Map of sampling effort. The sampling effort were estimated by density of records through kernel interpolation in ArcGIS 10.1. To establish the search radius of kernel estimation we used the average distance between points of occurrence. 4

3 - List of checklist references 4

4 - List of institutions that provided distribution data to Gbif and Specieslink............................6

5 - AoEs identified by GIE through species occurrence. Numbers indicate corresponding areas in table 6...9

6 -Endemic species restricted to the areas indicated figure 5. 10

7 - AoEs identified by GIE through subspecies occurrence. Numbers indicate corresponding areas in table 8. 13

8 - Endemic species restricted to the areas indicated figure 7. 14

9 - AoEs identified by NDM through species occurrence...20

10 - AoEs identified by NDM through subspecies occurrence. 25

11 - AoE identified by PAE through species occurrence. 45

12 - Consensus tree of PAE based on species occurrence. 46

13 - Tree of constrained PAE based on species occurrence...47

14 - AoEs identified by PAE through subspecies occurrence. 48

15 - Consensus tree of PAE based on subspecies occurrence..49

16 - Tree of constrained PAE based on subspecies occurrence. 50

17 - Autocorrelogram of Moran I analysis of three axis of NMDS analysis...............................51

18 - Most relevant breaks in species composition obtained by Monmonier's Algorithm..........52

19 - Interpolation of the three axes of NMDS based on species occurrence, subspecies occurrence. Numbers indicate correlation between maps...53

20 - Unsupervised classification of the spatial variation in species composition. Colours represent different groups in the classification. Each line represents a classification in the number of classes indicated at left...54

21- Interpolation of the three axes of NMDS based on species and subspecies occurrence. Beta-diversity is partitioned into turnover and nestedness components 56

22- Results of Discriminant Analysis. 57

1 - Step-by-step analysis of spatial variation in species composition. See the Material and methods for details. Map created in ArcGIS 10.1 (http://www.esri.com).

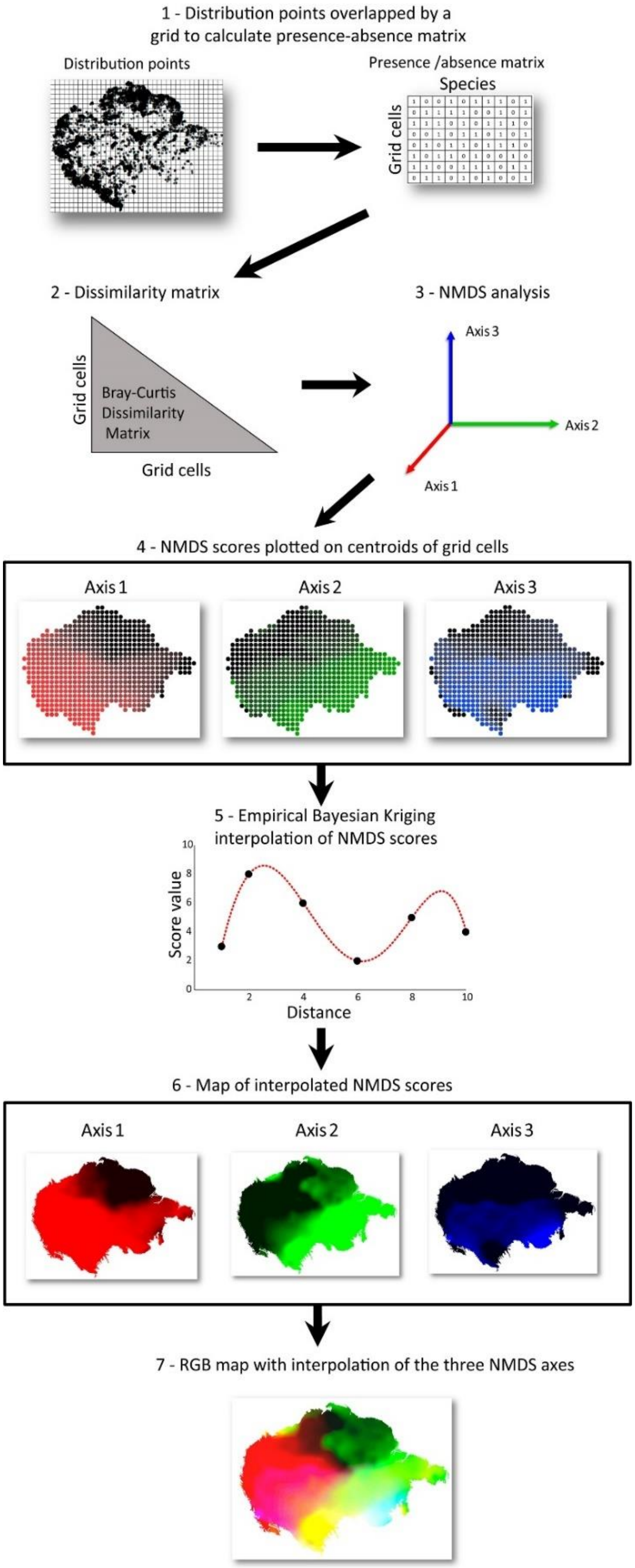

2 - Map of sampling effort. The sampling effort were estimated by density of records through kernel interpolation in ArcGIS 10.1. To establish the search radius of kernel estimation we used the average distance between points of occurrence.

Map created in ArcGIS 10.1 (http://www.esri.com)

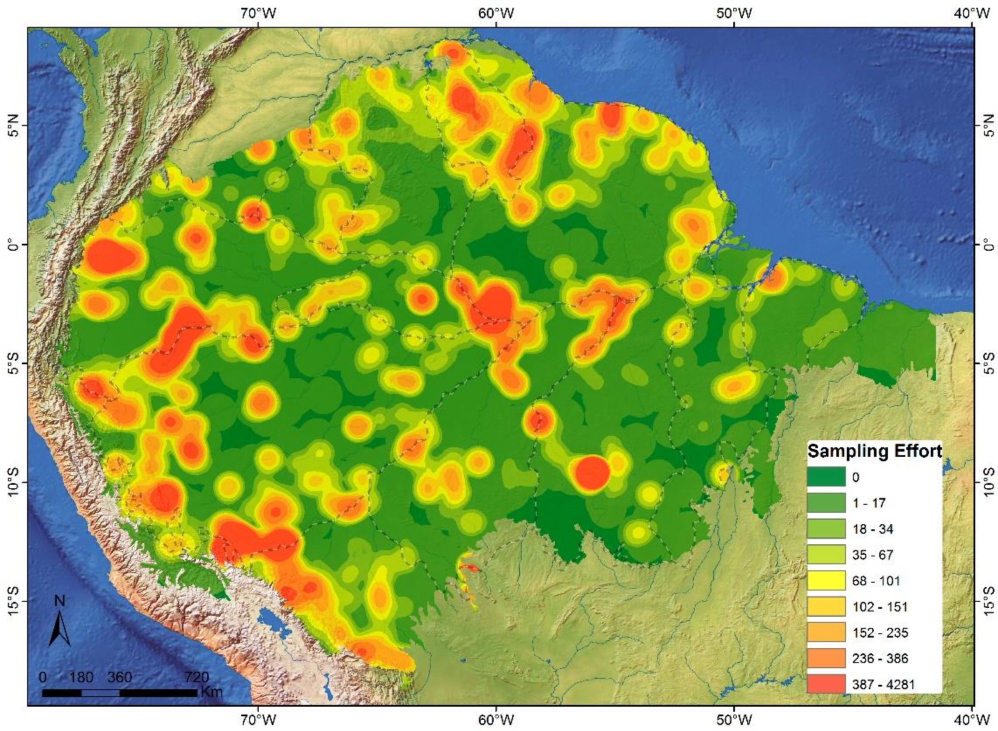

3 - List of checklist references

Borges S.H. & Almeida R.A.M. de (2011) Birds of the Jaú National Park and adjacent areas, Brazilian Amazon: new species records with reanalysis of a previous checklist. *Revista Brasileira de Ornitologia*, **19**, 108–133.

Dantas S. de M., Faccio M.S., & Lima M. de F. (2011) Avifaunal inventory of the Floresta Nacional de Pau-Rosa, Maués, state of Amazonas, Brazil. *Revista Brasileira de Entomologia*, **19**, 154–166.

Del-Rio G., Silveira L.F., Cavarzere V., & Rêgo M.A. (2013) A taxonomic review of the Golden-green Woodpecker, *Piculus chrysochloros* (Aves: Picidae) reveals the existence of six valid taxa. *Zootaxa*, **3626**, 531.

Dornas T. & Pinheiro R.T. (2011) Aves coligidas por José Hidasi e Manoel Santa-Brígida na Amazônia Tocantinense: implicações para a distribuição geográfica das aves amazônicas brasileiras. *Revista Brasileira de Ornitologia*, **19**, 276–301.

Fernandes A.M. (2007) Southern range extension for the Red-And-Black Grosbeak (*Periporphyrus erythromelas*, Cardinalidae), Amazonian, Brazil. *Revista Brasileira de Ornitologia*, **15**, 468–469.

Guilherme E. & Dantas S. de M. (2011) Avifauna of the Upper Purus River, State of Acre, Brazil. *Revista Brasileira de Ornitologia*, **19**, 185–199.

Lees A.C., Moura N.G. de, Andretti C.B., Davis B.J.W., Lopes E. V., Henriques L.M.P., Aleixo1 A., Barlow J., Ferreira J., & Gardner T.A. (2013) One hundred and thirty-five years of avifaunal surveys around Santarém, central Brazilian Amazon. *Revista Brasileira de Ornitologia*, **21**, 16–57.

Olmos F., Silveira L.F., & Benedicto G.A. (2011) A Contribution to the Ornithology of Rondônia, Southwest of the Brazilian Amazon. *Revista Brasileira de Ornitologia*, **19**, 200–229.

Portes C.E., Carneiro L.S.B., Schunck F., Silva M. de S., Zimmer K.J., Whittaker A., Poletto F., Silveira L.F., & Aleixo A. (2011) Annotated checklist of birds recorded between 1998 and 2009 at nine areas in the Belém area of endemism, with notes on some range extensions and the conservation status of endangered species. *Revista Brasileira de Ornitologia*, **19**, 167–184.

Santos M.P.D., Aleixo A., D'Horta F.M., & Portes C.E.B. (2011a) Avifauna of the Juruti Region, Pará, Brazil. *Revista Brasileira de Ornitologia*, **19**, 134–153.

Santos M.P.D., Silveira L.F., & Silva J.M.C. da (2011b) Birds of Serra do Cachimbo, Pará State, Brazil. *Revista Brasileira de Ornitologia*, **19**, 244–259.

Schunck F., Luca A.C. De, Piacentini V. de Q., Rego M.A., Rennó B., & Corrêa A.H. (2011) Avifauna of two localities in the south of Amapá, Brazil, with comments on the distribution and taxonomy of some species. *Revista Brasileira de Ornitologia*, **19**, 93–107.

Dantas M.P., Silva G.O., Reis A.L.(2011) Birds of the Igarapé Lourdes Indigenous Territory, Jí-Paraná, Rondônia, Brazil. *Revista Brasileira de Ornitologia*, **19**, 230–243.

Somenzari M., Silveira L.F., Piacentini V. de Q., Rego M.A., Schunck F., & Cavarzere V. (2011) Birds of an Amazonia-Cerrado ecotone in southern Pará, Brazil, and the efficiency of associating multiple methods in avifaunal inventories. *Revista Brasileira de Ornitologia*, **19**, 260–275.

Whittaker A. (2004) Noteworthy ornithological records from Rondonia, Brazil, including a first country record, comments on austral migration, life history, taxonomy and distribution, with relevant data from neighbouring states, and a first record for Bolivia. *British Ornithologists' Club*, **124**, 239–271.

4 - List of institutions that provided distribution data to Gbif and Specieslink.

Academy of Natural Sciences, Philadelphia

American Museum of Natural History

Australian National Wildlife Collection

Burke Museum of Natural History and Culture

Carnegie Museum of Natural History

Centro Universitário Moura Lacerda

Coleção Ornitológica Museu de Biologia Prof. Mello Leitão

Denver Museum of Nature & Science

Field Museum of Natural History Collection of Birds

Florida Museum of Natural History

Fundação Universidade do Tocantins

Fundação Universidade Federal do Rio Grande

Fundación Miguel Lillo - Colección Ornitológica

Fundación Puerto Rastrojo - Colombia

Instituto Adolfo Lutz São Paulo

Instituto de Investigación de Recursos Biológicos Alexander von Humboldt

Instituto Nacional de Pesquisas da Amazônia

KU Biodiversity Institute & Natural History Museum

Marjorie Barrick Museum of Natural History, University of Nevada - Las Vegas

Museo Argentino de Ciencias Naturales, Buenos Aires

Museu de História Natural Capão da Imbuia

Museu de Zoologia da Universidade de São Paulo

Museu Nacional, Universidade Federal do Rio de Janeiro

Muséum national d'Histoire naturelle

Museum of Comparative Zoology - Harvard University

Museum of Vertebrate Zoology of Berkeley

Museum of Zoology, University of Michigan

National Museum of Natural History, Smithsonian Institution

Natural History Museum London

Natural History Museum of Los Angeles County

Peabody Museum of Natural History, Yale University

Pontifícia Universidade Católica de Minas Gerais

Pontifícia Universidade Católica do Rio Grande do Sul

Provincial Museum of Alberta, Edmonton, AB, Canada

Royal Belgian Institute of natural Sciences

Royal Ontario Museum: ROM
San Diego Natural History Museum
Santa Barbara Museum of Natural History
Secretaria de Meio Ambiente - Acre
Senckenberg Museum, Frankfurt
Staatliches Museum Für Naturkunde Stuttgart
Universidade da Região de Joinville
Universidade de Brasília
Universidade do Estado de Mato Grosso
Universidade Estadual de Campinas
Universidade Estadual de Feira de Santana
Universidade Estadual de Maringá
Universidade Estadual Paulista
Universidade Federal da Bahia
Universidade Federal da Paraíba
Universidade Federal de Goiás
Universidade Federal de Juiz de Fora
Universidade Federal de Mato Grosso
Universidade Federal de Mato Grosso do Sul
Centro de Coleções Taxonômicas, Universidade Federal de Minas Gerais
Universidade Federal de Pernambuco
Universidade Federal de Rondônia
Universidade Federal de Santa Catarina
Universidade Federal de Sergipe
Universidade Federal de Viçosa
Universidade Federal do Ceará
Universidade Federal do Espírito Santo
Universidade Federal do Paraná
Universidade Federal do Paraná
Universidade Federal do Piauí
Universidade Federal do Rio Grande do Norte
Universidade Federal do Rio Grande do Sul
Universidade Federal do Vale do São Francisco
Universidade Federal dos Vales do Jequitinhonha e Mucuri
Universidade Federal Rural de Pernambuco
Universidade Federal Rural do Semi-Árido
Universidade Regional de Blumenau
University of East London

University of Wyoming Museum of Vertebrates

Zoological Museum, University of Amsterdam

5 - AoEs identified by GIE through species occurrence. Numbers indicate corresponding areas in table 6.

Map created in ArcGIS 10.1 (http://www.esri.com)

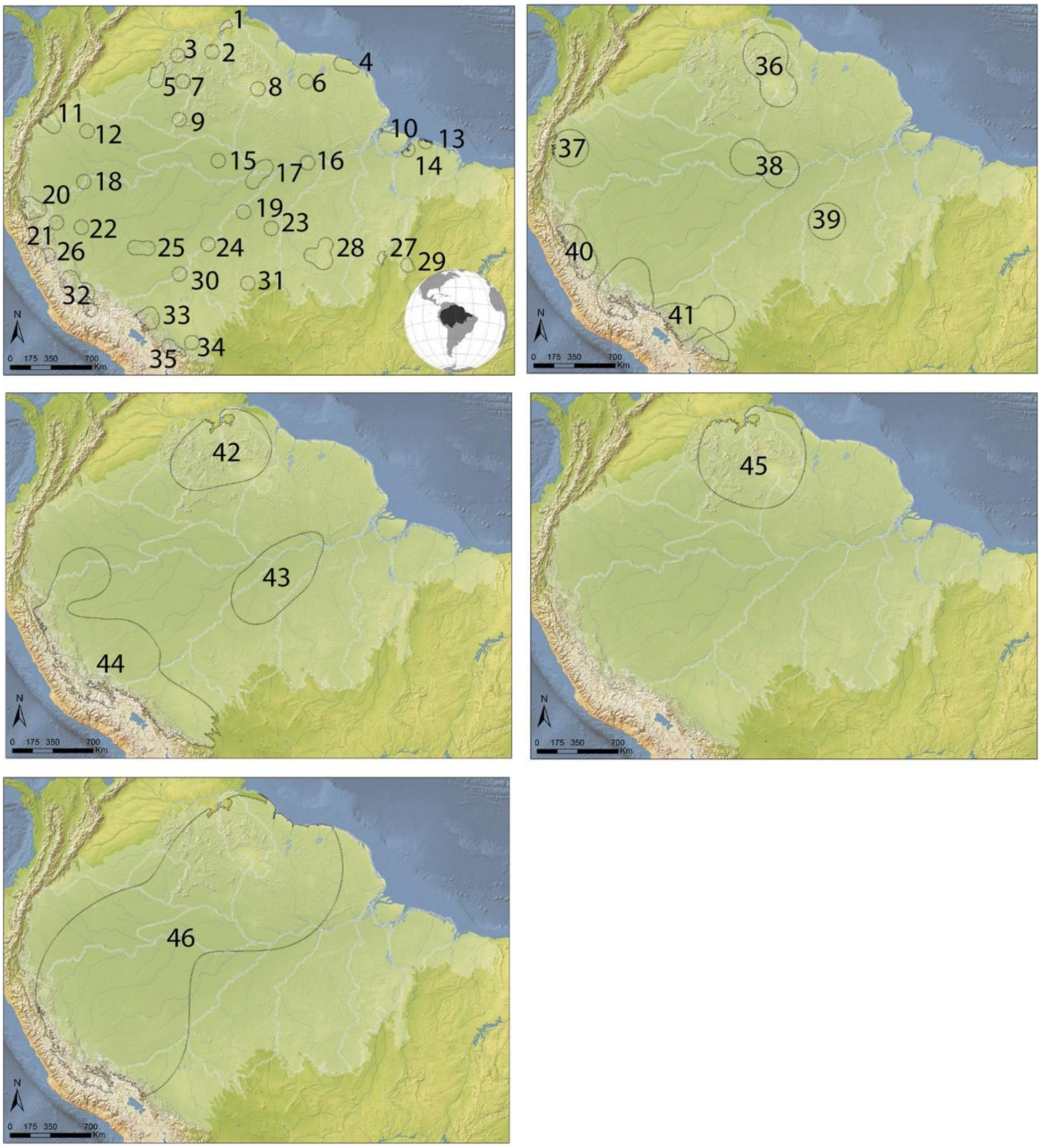

6 -Endemic species restricted to the areas indicated figure 5.

Area of Endemism	Sinendemic species
1	*Amaurospiza carrizalensis*
2	*Myioborus cardonai*
3	*Myioborus albifacies*
4	*Anas americana*
4	*Setopagis maculosa*
5	*Cyanocorax heilprini*
5	*Thripophaga cherriei*
6	*Aulacorhynchus whitelianus*
7	*Emberizoides duidae*
8	*Synallaxis kollari*
9	*Diglossa duidae*
10	*Xiphorhynchus guttatoides*
11	*Accipiter collaris*
11	*Dysithamnus occidentalis*
12	*Chlorostilbon olivaresi*
13	*Ardea cinerea*
14	*Turdus grayi*
15	*Polioptila facilis*
16	*Polioptila paraensis*
17	*Contopus albogularis*
17	*Polioptila attenboroughi*
17	*Thamnophilus melanothorax*
18	*Percnostola arenarum*
19	*Zimmerius chicomendesi*
20	*Grallaria przewalskii*
20	*Grallaricula ochraceifrons*
20	*Hemitriccus cinnamomeipectus*
20	*Herpsilochmus parkeri*
20	*Loddigesia mirabilis*
21	*Capito wallacei*
22	*Thamnophilus divisorius*
23	*Hypocnemis rondoni*
24	*Amazilia rondoniae*
25	*Cacicus latirostris*
25	*Hemitriccus cohnhafti*
26	*Cnemathraupis aureodorsalis*
27	*Piculus chrysochloros*
28	*Herpsilochmus sellowi*
28	*Hylonympha macrocerca*
28	*Phaethornis aethopyga*

Area of Endemism	Sinendemic species
28	*Synallaxis cinnamomea*
29	*Neomorphus squamiger*
30	*Hylopezus auricularis*
31	*Philydor erythrocercus*
32	*Asthenes palpebralis*
32	*Asthenes vilcabambae*
32	*Atlapetes melanopsis*
32	*Atlapetes terborghi*
32	*Cranioleuca marcapatae*
32	*Hemispingus parodii*
32	*Leptasthenura xenothorax*
32	*Metallura eupogon*
33	*Asthenes helleri*
33	*Schistocichla brunneiceps*
33	*Terpsiphone smithii*
34	*Discosura letitiae*
35	*Cranioleuca henricae*
36	*Campylopterus hyperythrus*
36	*Diglossa major*
36	*Mitrospingus oleagineus*
36	*Pipreola whitelyi*
37	*Epinecrophylla fjeldsaai*
38	*Percnostola minor*
38	*Stigmatura napensis*
39	*Lepidothrix vilasboasi*
40	*Grallaria capitalis*
41	*Anairetes alpinus*
41	*Ara glaucogularis*
41	*Cacicus koepckeae*
41	*Grallaria erythroleuca*
41	*Phlogophilus harterti*
42	*Elaenia dayi*
42	*Lipaugus streptophorus*
42	*Poecilotriccus russatus*
42	*Pyrrhura egregia*
42	*Setopagis whitelyi*
43	*Dendrocolaptes hoffmannsi*
43	*Picumnus varzeae*
43	*Rhegmatorhina berlepschi*
43	*Tolmomyias sucunduri*
44	*Aglaeactis castelnaudii*
44	*Amazilia viridicauda*
44	*Andigena cucullata*
44	*Atlapetes melanolaemus*

Area of Endemism	Sinendemic species
44	*Cinclodes aricomae*
44	*Conioptilon mcilhennyi*
44	*Conothraupis speculigera*
44	*Cranioleuca albicapilla*
44	*Cranioleuca albiceps*
44	*Creurgops dentatus*
44	*Formicarius rufifrons*
44	*Hemispingus calophrys*
44	*Hemitriccus spodiops*
44	*Herpsilochmus motacilloides*
44	*Lepidothrix coeruleocapilla*
44	*Lipaugus uropygialis*
44	*Myiophobus inornatus*
44	*Myrmoborus melanurus*
44	*Nothoprocta taczanowskii*
44	*Pipreola pulchra*
45	*Lophornis pavoninus*
45	*Polytmus milleri*
46	*Aramides calopterus*
46	*Galbalcyrhynchus purusianus*

7 - AoEs identified by GIE through subspecies occurrence. Numbers indicate corresponding areas in table 8.

Map created in ArcGIS 10.1 (http://www.esri.com)

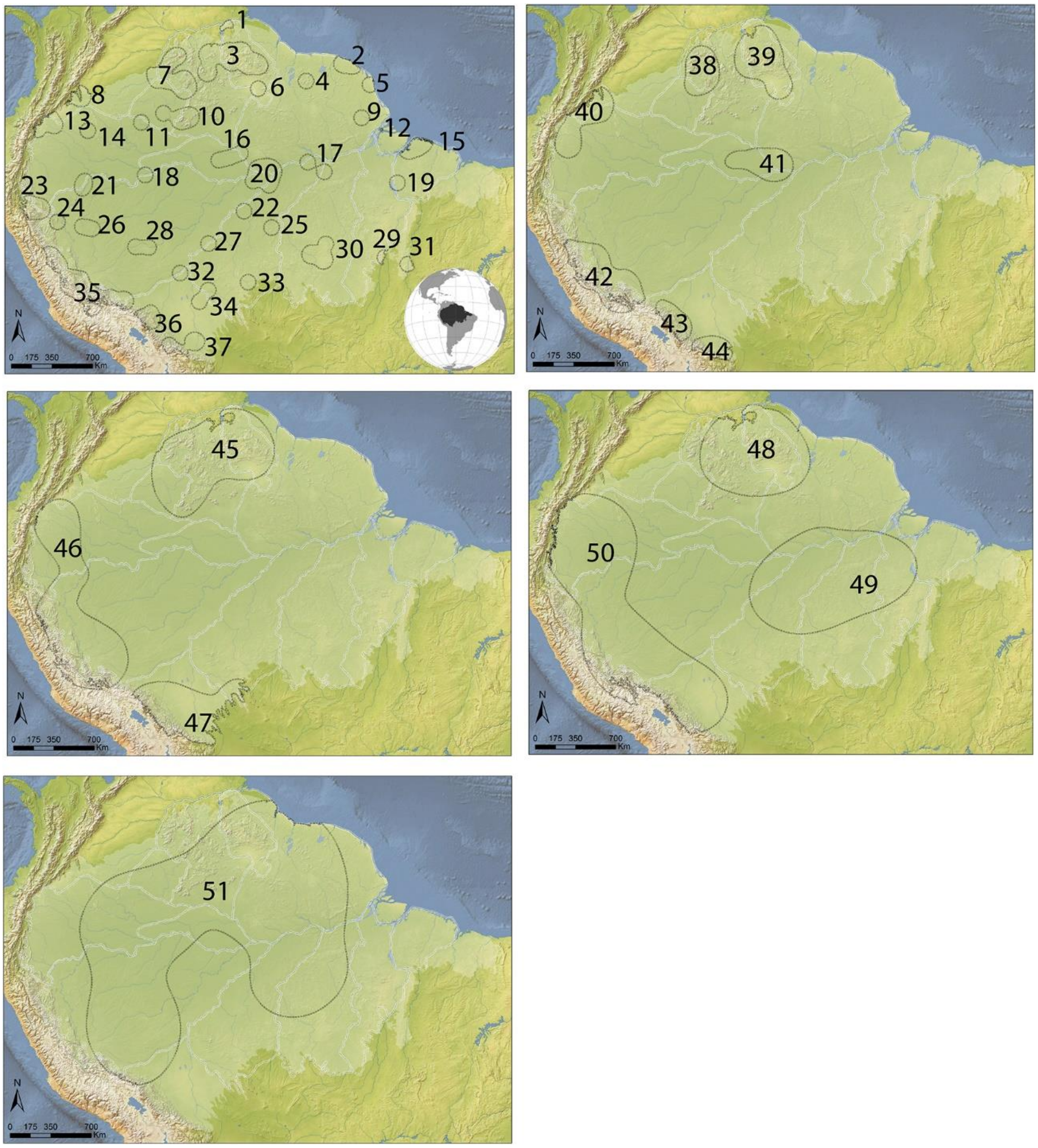

8 - Endemic species restricted to the areas indicated figure 7.

Area of Endemism	Sinendemic species
1	*Amaurospiza carrizalensis*
2	*Amazilia brevirostris orienticola*
2	*Anas americana*
2	*Setopagis maculosa*
3	*Atlapetes personatus collaris*
3	*Atlapetes personatus personatus*
3	*Campylopterus duidae guaiquinimae*
3	*Crypturellus ptaritepui*
3	*Diglossa major chimantae*
3	*Diglossa major disjuncta*
3	*Diglossa major gilliardi*
3	*Diglossa major major*
3	*Elaenia dayi auyantepui*
3	*Elaenia dayi dayi*
3	*Microcerculus ustulatus obscurus*
3	*Mitrospingus oleagineus oleagineus*
3	*Myioborus cardonai*
3	*Myiophobus roraimae roraimae*
3	*Myrmothera simplex guaiquinimae*
3	*Myrmothera simplex pacaraimae*
3	*Myrmotherula behni inornata*
3	*Myrmotherula behni yavii*
3	*Pheugopedius coraya obscurus*
3	*Pipreola whitelyi kathleenae*
3	*Pipreola whitelyi whitelyi*
3	*Roraimia adusta mayri*
3	*Schistocichla leucostigma saturata*
3	*Troglodytes rufulus rufulus*
3	*Xenopipo uniformis uniformis*
4	*Aulacorhynchus whitelianus*
5	*Thamnophilus nigrocinereus kulczynskii*
6	*Synallaxis kollari*
7	*Atlapetes personatus parui*
7	*Cyanocorax heilprini*
7	*Emberizoides duidae*
7	*Epinecrophylla ornata*
7	*Galbula dea*
7	*Hemitriccus minor*
7	*Knipolegus poecilurus paraquensis*
7	*Lophornis pavoninus duidae*
7	*Myioborus albifacies*
7	*Roraimia adusta duidae*

Area of Endemism	Sinendemic species
7	*Roraimia adusta obscurodorsalis*
7	*Thripophaga cherriei*
7	*Troglodytes rufulus duidae*
7	*Troglodytes rufulus marahuacae*
7	*Troglodytes rufulus yavii*
8	*Cercomacra nigrescens*
8	*Epinecrophylla ornata ornata*
9	*Threnetes leucurus loehkeni*
10	*Atlapetes personatus jugularis*
10	*Cranioleuca demissa cardonai*
10	*Diglossa duidae hitchcocki*
10	*Frederickena unduligera*
10	*Heliodoxa xanthogonys willardi*
10	*Myioborus castaneocapilla maguirei*
10	*Troglodytes rufulus wetmorei*
11	*Hylexetastes stresemanni insignis*
12	*Xiphorhynchus guttatoides*
13	*Accipiter collaris*
13	*Dysithamnus occidentalis*
13	*Hylopezus fulviventris caquetae*
13	*Microbates collaris colombianus*
13	*Synallaxis cherriei napoensis*
14	*Chlorostilbon olivaresi*
15	*Ardea cinerea*
15	*Dendrexetastes rufigula paraensis*
15	*Philydor erythropterum diluviale*
15	*Picumnus spilogaster pallidus*
15	*Psophia crepitans*
15	*Turdus grayi*
16	*Picumnus lafresnayi pusillus*
16	*Polioptila facilis*
16	*Sclateria naevia*
17	*Lepidothrix iris iris*
17	*Polioptila paraensis*
18	*Thamnophilus nigrocinereus*
19	*Nonnula ruficapilla inundata*
20	*Brotogeris chrysoptera solimoensis*
20	*Celeus grammicus*
20	*Contopus albogularis*
20	*Hylophilus hypoxanthus albigula*
20	*Microcerculus ustulatus*
20	*Myrmoborus lugubris femininus*
20	*Pipra aureola flavicollis*
20	*Polioptila attenboroughi*

Area of Endemism	Sinendemic species
20	*Thamnophilus melanothorax*
21	*Percnostola arenarum*
21	*Percnostola rufifrons jensoni*
22	*Zimmerius chicomendesi*
23	*Brotogeris cyanoptera gustavi*
23	*Grallaria przewalskii*
23	*Grallaricula ochraceifrons*
23	*Hemitriccus cinnamomeipectus*
23	*Herpsilochmus parkeri*
23	*Loddigesia mirabilis*
23	*Myrmeciza castanea castanea*
23	*Pheugopedius coraya albiventris*
24	*Capito wallacei*
25	*Hypocnemis rondoni*
26	*Tangara punctata annectens*
26	*Thamnophilus divisorius*
27	*Amazilia rondoniae*
28	*Cacicus latirostris*
28	*Hemitriccus cohnhafti*
29	*Piculus chrysochloros*
30	*Herpsilochmus sellowi*
30	*Hylonympha macrocerca*
30	*Phaethornis aethopyga*
30	*Pyrrhura lepida lepida*
30	*Synallaxis cinnamomea*
31	*Neomorphus squamiger*
32	*Hylopezus auricularis*
33	*Philydor erythrocercus*
34	*Lampropsar tanagrinus violaceus*
34	*Synallaxis cabanisi cabanisi*
35	*Aglaeactis castelnaudii*
35	*Aglaeactis castelnaudii*
35	*Asthenes palpebralis*
35	*Asthenes vilcabambae*
35	*Atlapetes melanopsis*
35	*Atlapetes terborghi*
35	*Cinnycerthia fulva fitzpatricki*
35	*Cinnycerthia fulva fulva*
35	*Cnemathraupis aureodorsalis*
35	*Cranioleuca albicapilla albigula*
35	*Cranioleuca marcapatae weskei*
35	*Hemispingus parodii*
35	*Leptasthenura xenothorax*
35	*Metallura eupogon*

Area of Endemism	Sinendemic species
35	*Pipreola intermedia intermedia*
35	*Synallaxis cherriei*
35	*Tangara chilensis*
35	*Tangara punctata perenensis*
36	*Asthenes harterti harterti*
36	*Asthenes helleri*
36	*Cranioleuca albiceps albiceps*
36	*Schistocichla brunneiceps*
36	*Terpsiphone smithii*
37	*Cranioleuca henricae*
37	*Discosura letitiae*
37	*Pteroglossus azara*
38	*Xenopipo uniformis duidae*
39	*Campylopterus hyperythrus*
39	*Microcerculus ustulatus ustulatus*
39	*Mitrospingus oleagineus obscuripectus*
39	*Myioborus castaneocapilla castaneocapilla*
39	*Pheugopedius coraya barrowcloughianus*
39	*Pyrrhura egregia egregia*
39	*Schistocichla leucostigma obscura*
39	*Thamnophilus insignis insignis*
39	*Troglodytes rufulus fulvigularis*
40	*Colinus cristatus leucotis*
40	*Myrmotherula behni behni*
41	*Frederickena unduligera unduligera*
41	*Myrmoborus lugubris stictopterus*
41	*Percnostola minor*
41	*Percnostola rufifrons subcristata*
42	*Atlapetes canigenis*
42	*Cranioleuca albicapilla albicapilla*
42	*Grallaria capitalis*
42	*Grallaria erythroleuca*
42	*Hypocnemis subflava subflava*
42	*Myrmoborus leucophrys koenigorum*
42	*Pheugopedius coraya cantator*
42	*Synallaxis gujanensis canipileus*
43	*Anairetes alpinus bolivianus*
44	*Asthenes harterti bejaranoi*
44	*Cranioleuca albiceps discolor*
45	*Atlapetes personatus paraquensis*
45	*Campylopterus duidae duidae*
45	*Cranioleuca demissa demissa*
45	*Gymnopithys rufigula pallidus*

Area of Endemism	Sinendemic species
45	*Herpsilochmus roraimae kathleenae*
45	*Hyloctistes subulatus lemae*
45	*Lipaugus streptophorus*
45	*Microcerculus ustulatus duidae*
45	*Myiophobus roraimae sadiecoatsae*
45	*Myrmothera simplex duidae*
45	*Myrmothera simplex simplex*
45	*Phylloscartes chapmani chapmani*
45	*Phylloscartes chapmani duidae*
45	*Poecilotriccus russatus*
45	*Psarocolius angustifrons oleagineus*
45	*Pyrrhura egregia obscura*
45	*Roraimia adusta adusta*
45	*Schistocichla caurensis caurensis*
45	*Setopagis whitelyi*
45	*Synallaxis macconnelli macconnelli*
45	*Thamnophilus insignis nigrofrontalis*
45	*Tyrannus dominicensis*
46	*Amazilia viridicauda*
46	*Atlapetes melanolaemus*
46	*Cercomacra nigrescens notata*
46	*Cinclodes aricomae*
46	*Doliornis sclateri*
46	*Epinecrophylla spodionota sororia*
46	*Herpsilochmus motacilloides*
46	*Hypocnemis cantator subflava*
46	*Lepidothrix coeruleocapilla*
46	*Picumnus lafresnayi taczanowskii*
46	*Pipreola pulchra*
46	*Pyrrhura rupicola rupicola*
46	*Rhegmatorhina melanosticta brunneiceps*
46	*Topaza pyra amaruni*
47	*Andigena cucullata*
47	*Creurgops dentatus*
47	*Dendrocincla merula remota*
47	*Hemispingus calophrys*
47	*Hemitriccus spodiops*
47	*Lipaugus uropygialis*
47	*Metallura aeneocauda malagae*
47	*Myiophobus inornatus*
47	*Pipreola intermedia signata*
47	*Tangara punctata punctulata*
48	*Herpsilochmus roraimae roraimae*
48	*Polytmus milleri*

Area of Endemism	Sinendemic species
48	*Tangara xanthogastra phelpsi*
49	*Capito brunneipectus*
49	*Euchrepomis spodioptila meridionalis*
49	*Hypocnemis hypoxantha ochraceiventris*
50	*Basileuterus chrysogaster*
50	*Eubucco tucinkae*
50	*Frederickena fulva*
50	*Heliodoxa branickii*
50	*Heterocercus aurantiivertex*
50	*Hylopezus fulviventris fulviventris*
50	*Metallura aeneocauda aeneocauda*
50	*Nannopsittaca dachilleae*
51	*Hemitriccus inornatus*
51	*Pteroglossus azara azara*
51	*Synallaxis rutilans confinis*

9 - AoEs identified by NDM through species occurrence.

Consensus area 0 of 9 (from 15 areas; max. values)

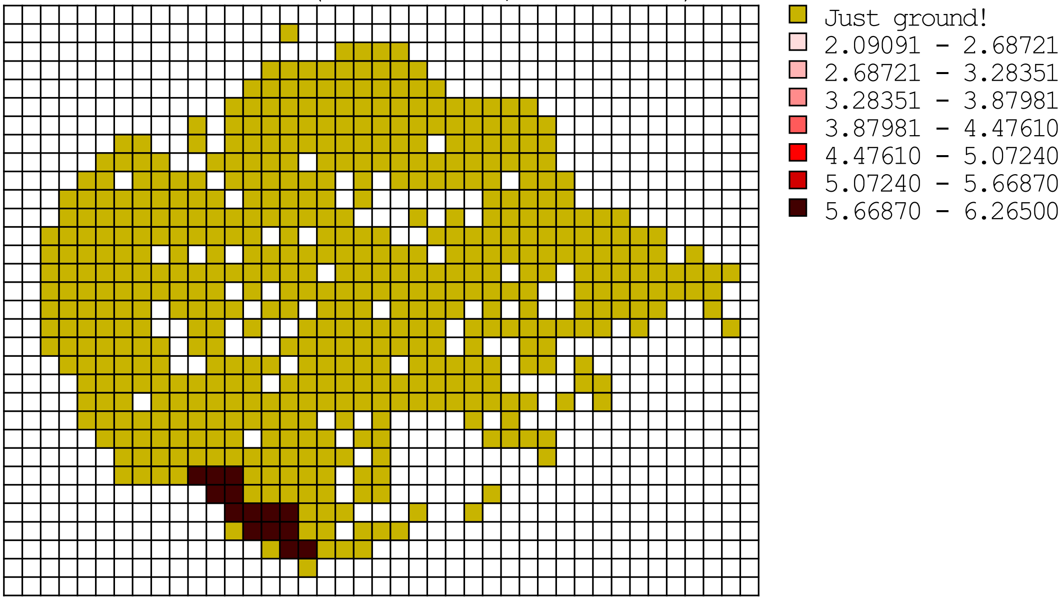

10 species give score:

Asthenes_harterti (0.636-1.000)
Asthenes_urubambensis (0.000-1.000)
Creurgops_dentatus (0.000-0.563)
Hemispingus_calophrys (0.000-0.619)
Myiophobus_inornatus (0.000-0.750)
Myiotheretes_fuscorufus (0.000-0.643)
Cranioleuca_albiceps (0.000-0.833)
Grallaria_erythrotis (0.000-0.783)
Hemispingus_trifasciatus (0.000-0.710)
Odontophorus_balliviani (0.000-0.507)

Consensus area 1 of 9 (from 10 areas; max. values)

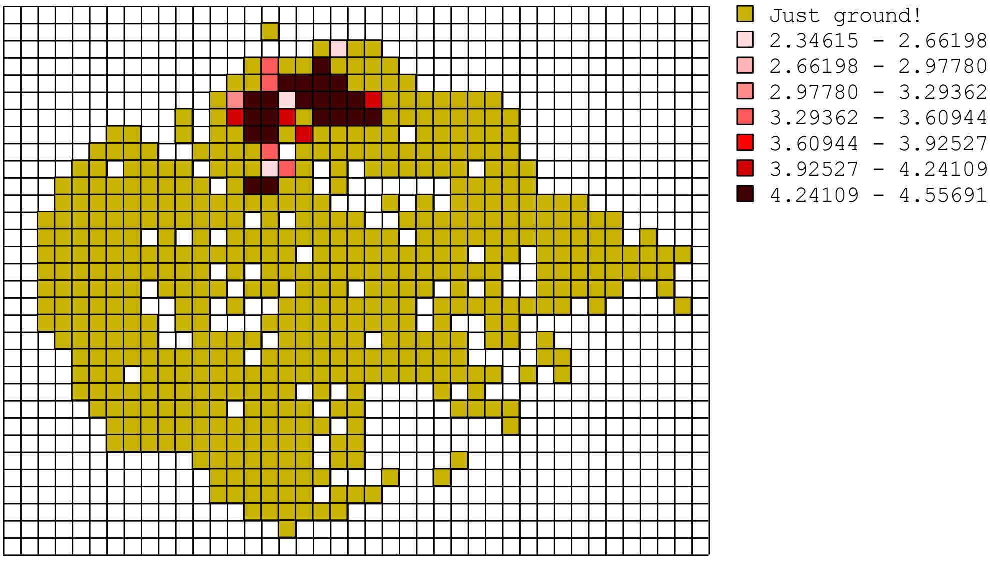

10 species give score:

Atlapetes_melanolaemus (0.000-0.643)
Ceratopipra_cornuta (0.000-0.747)
Grallaria_erythroleuca (0.000-0.750)
Myioborus_castaneocapilla (0.000-0.762)
Phlogophilus_harterti (0.000-0.875)
Atlapetes_personatus (0.000-0.827)
Cranioleuca_demissa (0.000-0.717)
Herpsilochmus_roraimae (0.000-0.672)
Myrmothera_simplex (0.000-0.808)
Phylloscartes_nigrifrons (0.000-0.743)

Consensus area 2 of 9 (from 2 areas; max. values)

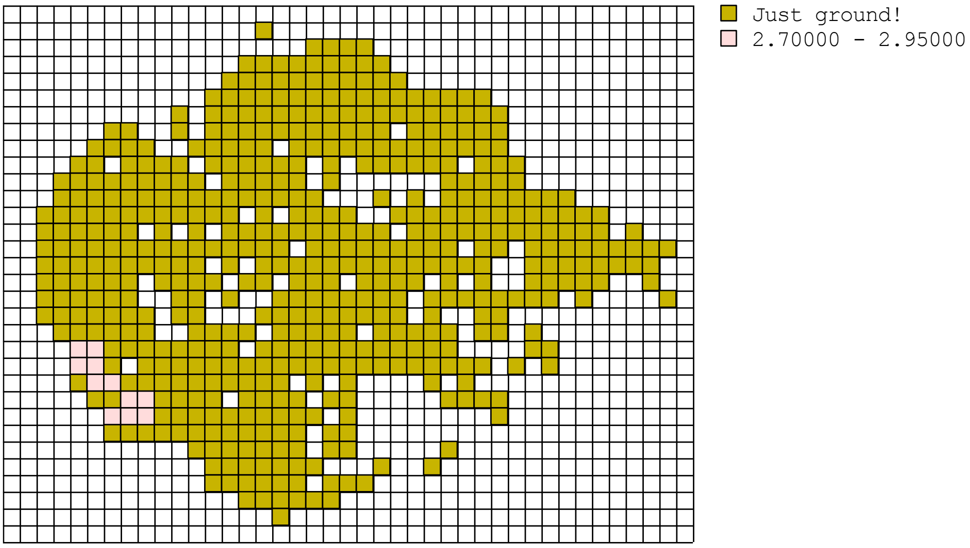

4 species give score:
Amazilia_viridicauda(0.500-0.800) Herpsilochmus_motacilloides(0.750-0.857)
Lepidothrix_coeruleocapilla(0.629-0.667) Pipreola_pulchra(0.650-0.714)

Consensus area 3 of 9 (from 1 areas; max. values)

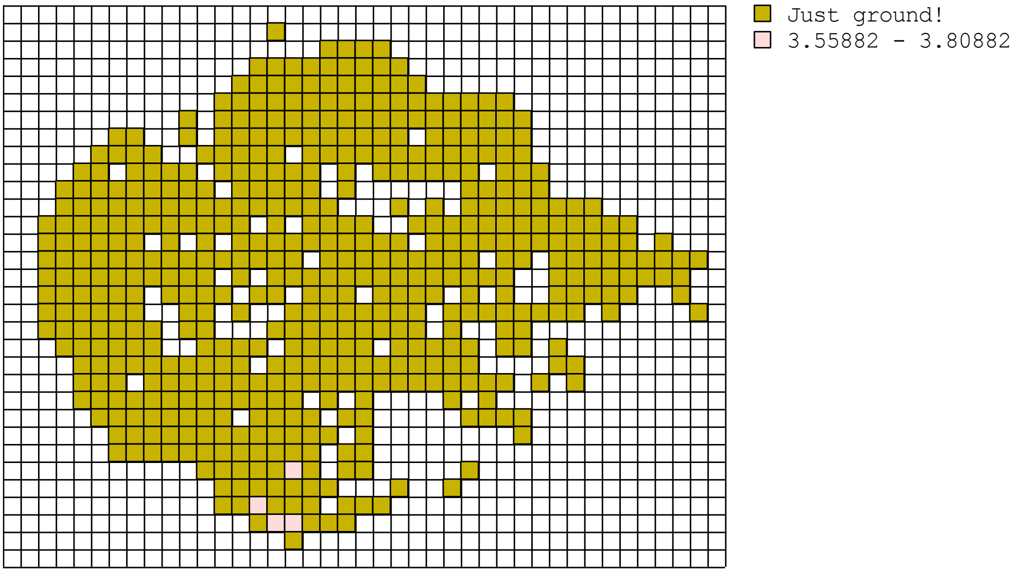

5 species give score:
Aglaeactis_pamela(0.669) Andigena_cucullata(0.566)
Ara_rubrogenys(0.669) Compsosp_iza_garleppi(0.772)
Hapalopsittaca_melanotis(0.882)

Consensus area 4 of 9 (from 1 areas; max. values)

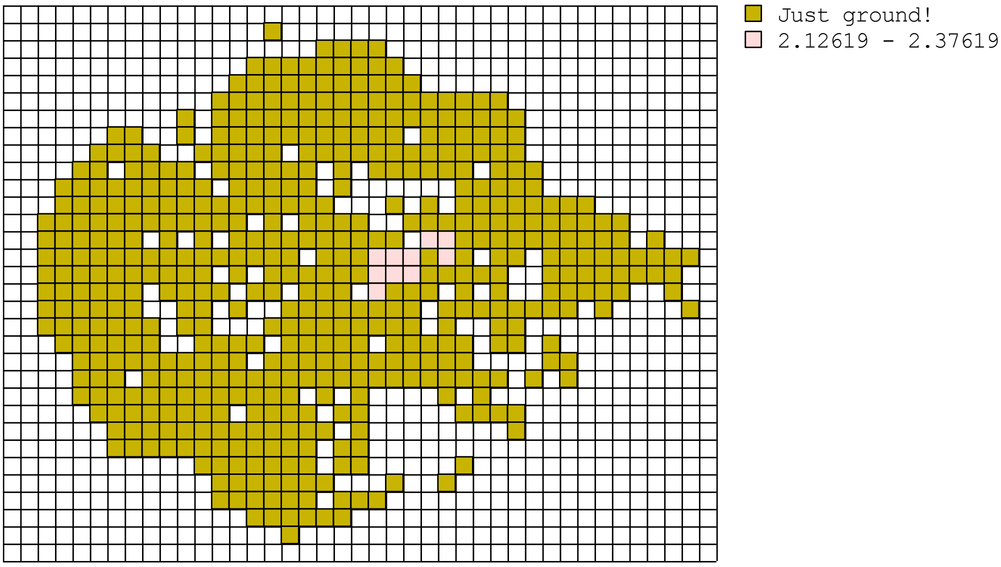

3 species give score:

Picumnus_varzeae(0.750)
Rhegmatorhina_berlepschi(0.607)
Tolmomyias sucunduri(0.769)

Consensus area 5 of 9 (from 1 areas; max. values)

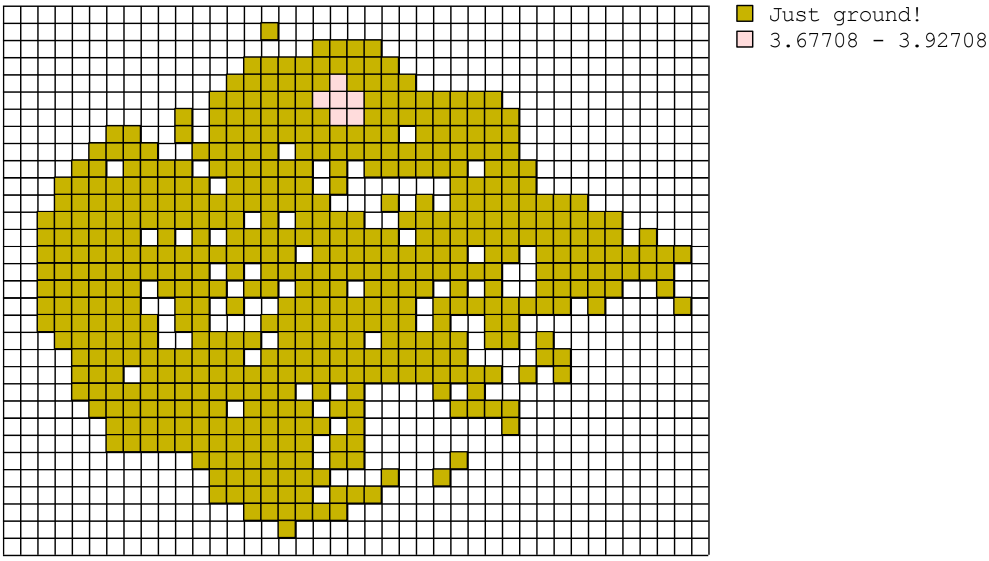

5 species give score:

Campylopterus_hyperythrus(0.729)
Diglossa_major(0.833)
Mitrosp ingus_oleagineus(0.875)
Pipreola_whitelyi(0.656)
Setopagis whitelyi(0.583)

Consensus area 6 of 9 (from 1 areas; max. values)

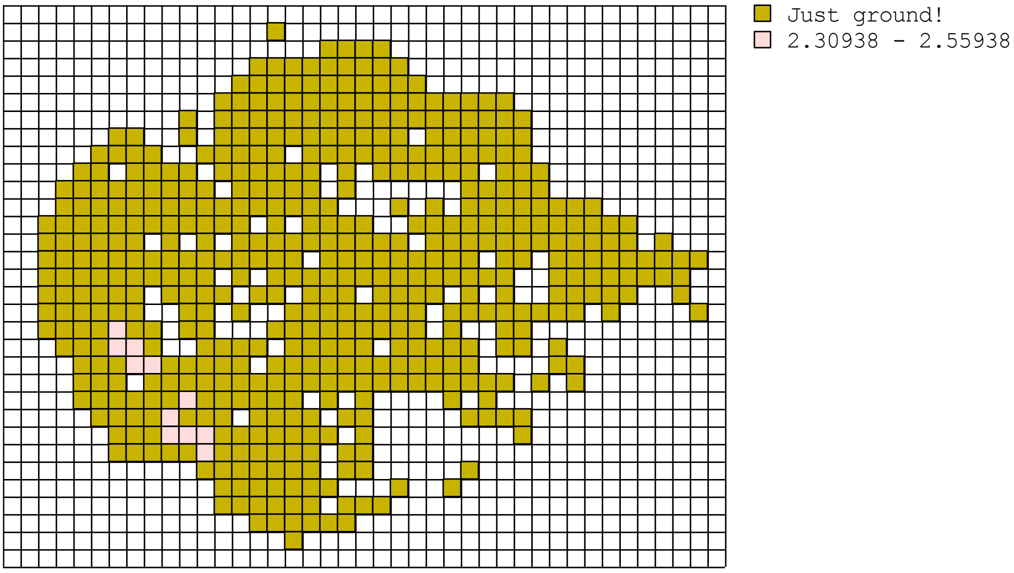

3 species give score:
Eubucco_tucinkae(0.864) Grallaria_eludens(0.680)
Nannopsittaca_dachilleae(0.765)

Consensus area 7 of 9 (from 1 areas; max. values)

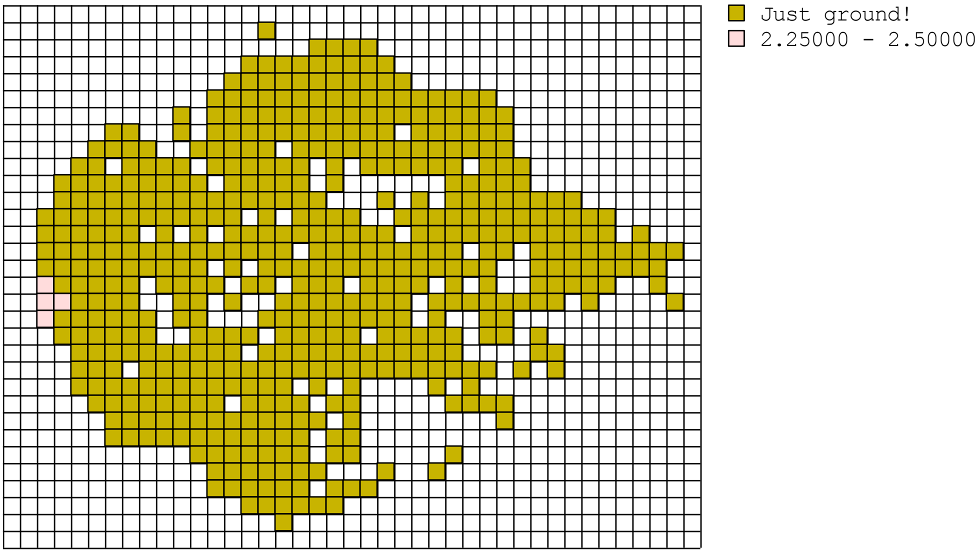

3 species give score:
Grallaria_przewalskii(0.750) Grallaricula_ochraceifrons(0.750)
Hemitriccus_cinnamomeipectus(0.750)

Consensus area 8 of 9 (from 1 areas; max. values)

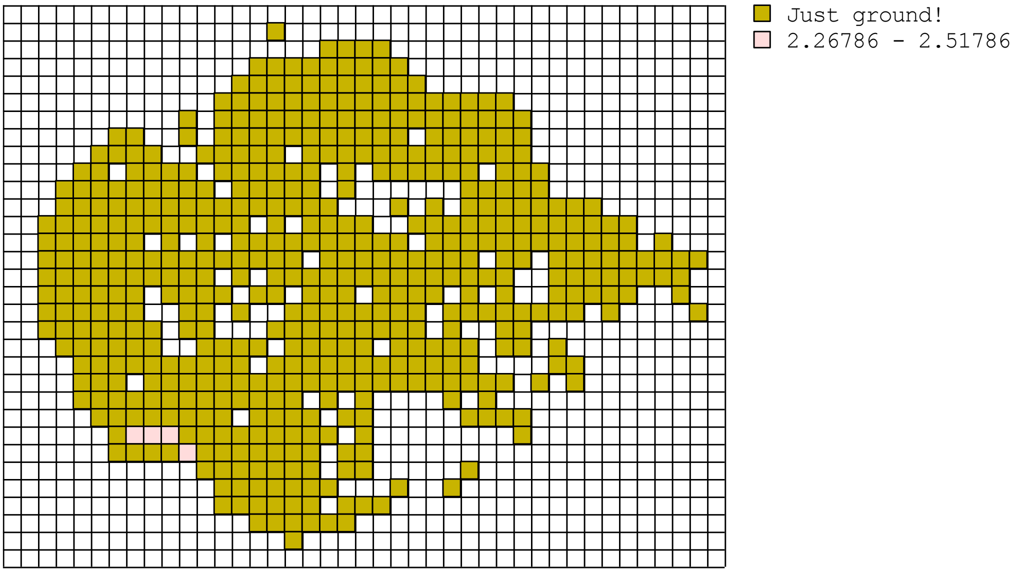

3 species give score:

Atlapetes_melanolaemus(0.643) Grallaria_erythroleuca(0.750)

Phlogophilus harterti(0.875)

10 - AoEs identified by NDM through subspecies occurrence.

Consensus area 0 of 41 (from 1 areas; max. values)

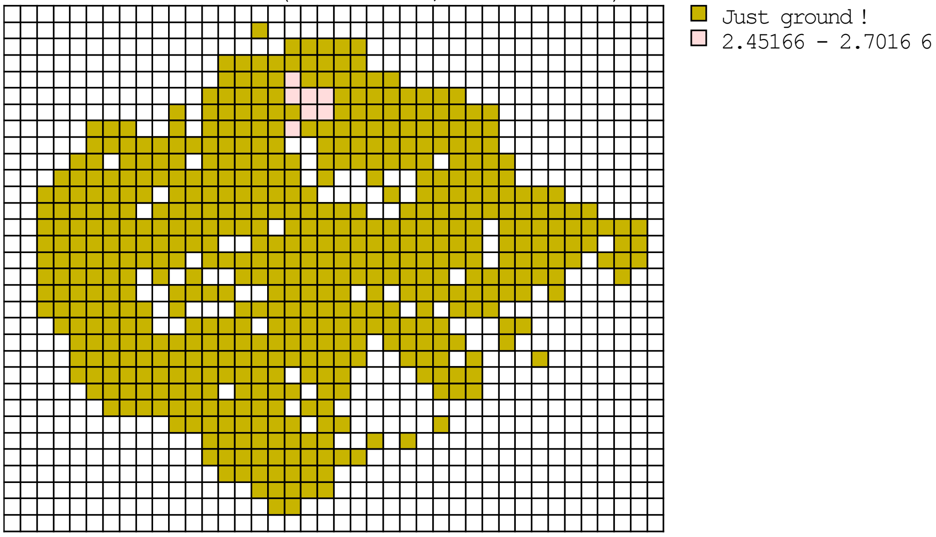

5 species give score :
157 Elaenia_dayi_auyantepui (0.286)
302 Microbates_collaris_paraguensis (0.182)
400 Phylloscartes_chapmani_chapmani (0.714)
500 Synallaxis_macconnelli_yavii (0.714)
581 Troglodytes_rufulus_fulvigularis (0.556)

Consensus area 1 of 41 (from 11 areas; max. values)

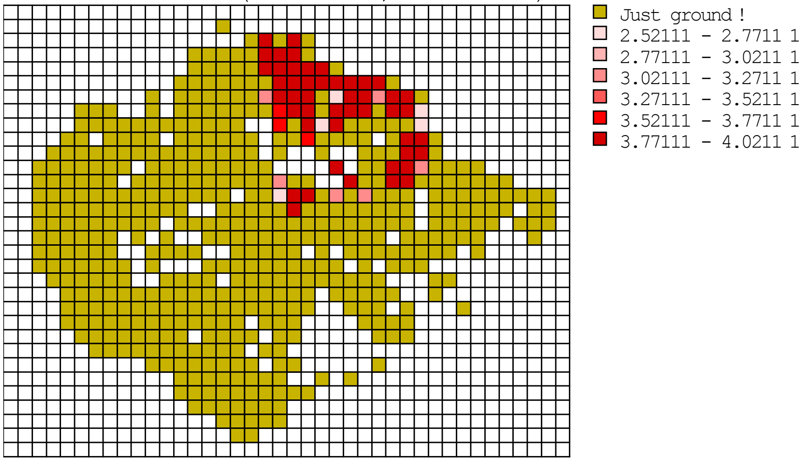

8 species give score :
112 Campylorhamphus_procurvoides_procurvoides (0.000-0.596)
170 Formicarius_analis_crissalis (0.000-0.667)
188 Gymnopithys_rufigula_rufigula (0.600-0.741)
308 Myrmeciza_ferruginea_ferruginea (0.000-0.614)
378 Pithys_albifrons_albifrons (0.000-0.896)
677 Capito_niger_ (0.462-0.765)
757 Epinecrophylla_gutturalis_ (0.542-0.607)
1087 Tyranneutes_virescens_ (0.000-0.316)

Consensus area 2 of 41 (from 1 areas; max. values)

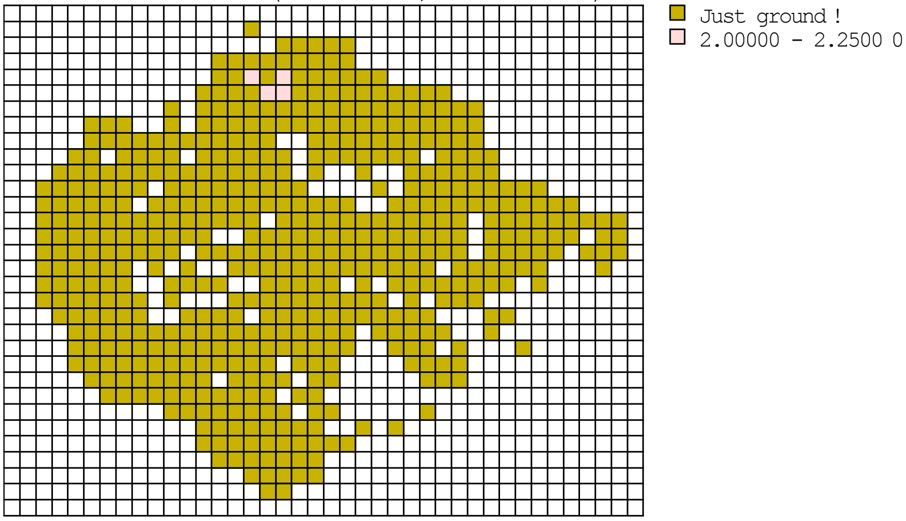

4 species give score :
302 Microbates_collaris_paraguensis(0.250)
356 Myrmothera_simplex_guaiquinimae(0.500)
365 Microcerculus_ustulatus_lunatipectus(0.750)
896 Myioborus_cardonai_(0.500)

Consensus area 3 of 41 (from 1 areas; max. values)

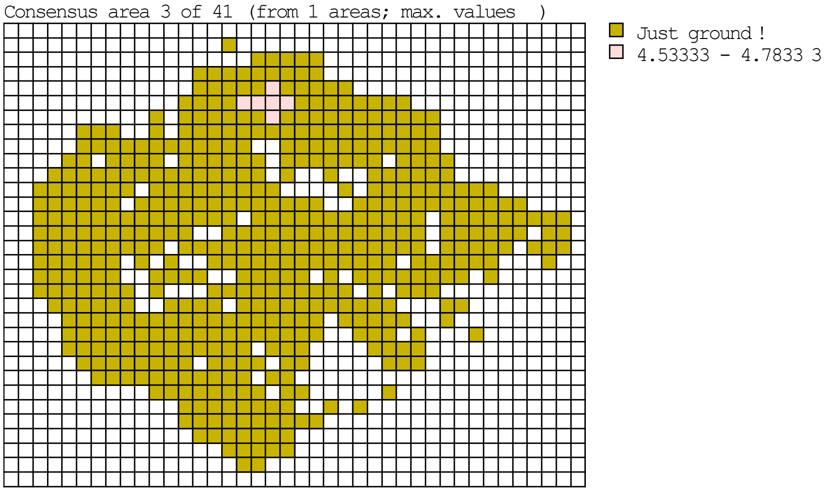

15 species give score :
18 Atlapetes_personatus_collaris(0.250)
73 Cranioleuca_demissa_demissa(0.188)
157 Elaenia_dayi_auyantepui(0.333)
239 Hyloctistes_subulatus_lemae(0.188)
292 Myioborus_castaneocapilla_castaneocapilla(0.333)
302 Microbates_collaris_paraguensis(0.375)
346 Mitrospingus_oleagineus_obscuripectus(0.500)
400 Phylloscartes_chapmani_chapmani(0.500)
411 Pyrrhura_egregia_obscura(0.200)
424 Schistocichla_leucostigma_obscura(0.167)
500 Synallaxis_macconnelli_yavii(0.167)
549 Thamnophilus_insignis_insignis(0.500)
670 Campylopterus_hyperythrus_(0.250)
731 Crypturellus_ptaritepui_(0.333)
1053 Setopagis_whitelyi_(0.250)

Consensus area 4 of 41 (from 1 areas; max. values)

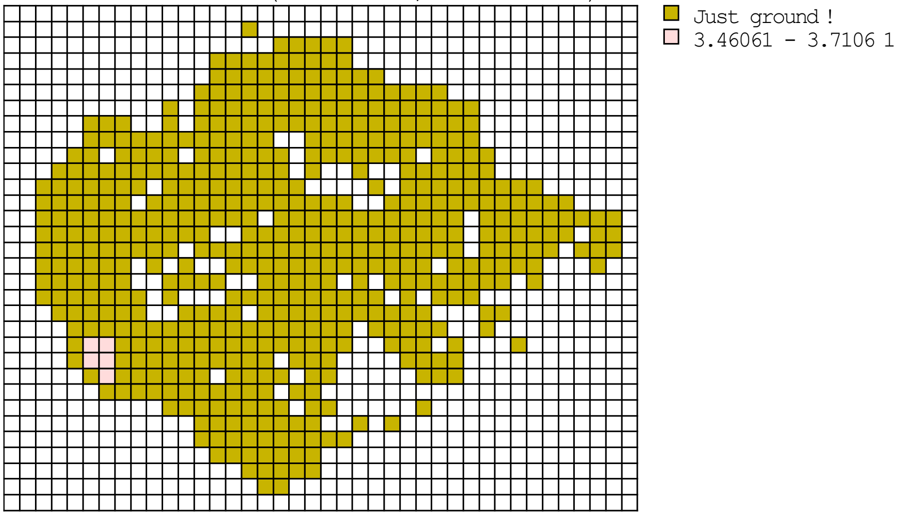

8 species give score :

42 Cranioleuca_albicapilla_albicapilla(0.273)
243 Hypocnemis_subflava_subflava(0.333)
323 Myrmoborus_leucophrys_koenigorum(0.714)
531 Pheugopedius_coraya_cantator(1.000)
646 Atlapetes_melanopsis_(0.286)
788 Grallaria_capitalis_(0.182)
825 Herpsilochmus_motacilloides_(0.273)
1071 Tangara_chilensis_(0.400)

Consensus area 5 of 41 (from 1 areas; max. values)

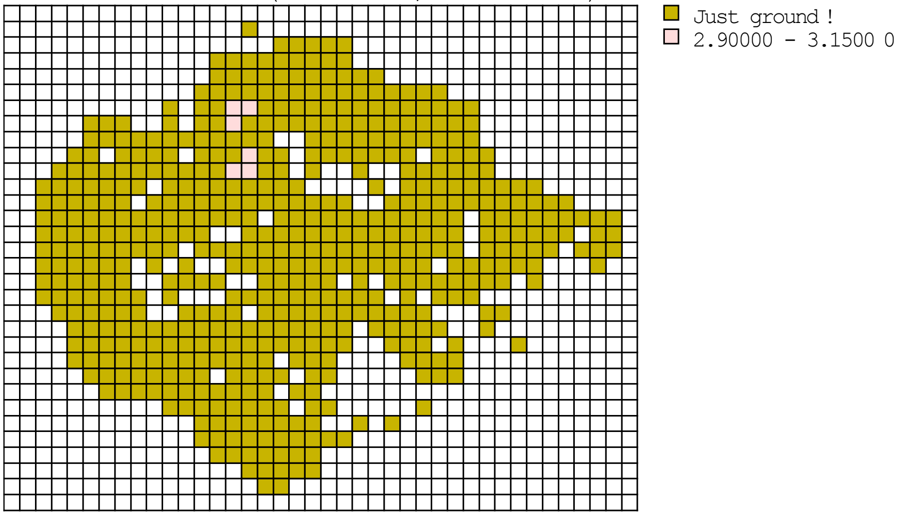

7 species give score :

74 Campylopterus_duidae_duidae(0.625)
232 Herpsilochmus_roraimae_kathleenae(0.500)
287 Microcerculus_bambla_caurensis(0.167)
355 Myrmothera_simplex_duidae(0.333)
364 Microcerculus_ustulatus_duidae(0.375)
399 Schistocichla_caurensis_caurensis(0.500)
550 Thamnophilus_insignis_nigrofrontalis(0.400)

Consensus area 6 of 41 (from 1 areas; max. values)

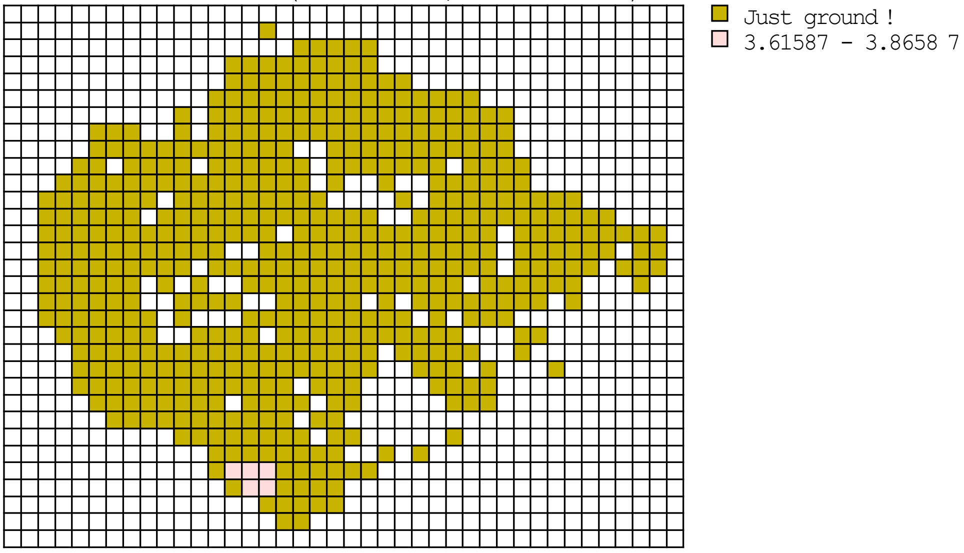

7 species give score :

9 Asthenes_harterti_bejaranoi (0.800)
52 Cranioleuca_albiceps_discolor (0.800)
282 Metallura_aeneocauda_malagae (0.286)
615 Aglaeactis_pamela_ (0.600)
626 Andigena_cucullata_ (0.444)
707 Compsospiza_garleppi_ (0.286)
862 Leptasthenura_yanacensis_ (0.400)

Consensus area 7 of 41 (from 4 areas; max. values)

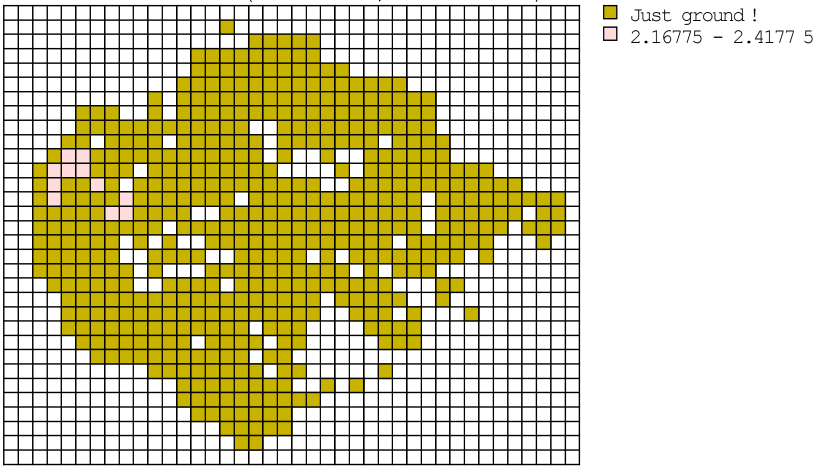

5 species give score :

28 Anabacerthia_ruficaudata_subflavescens (0.500-0.750)
208 Hylopezus_fulviventris_fulviventris (0.750)
212 Hylophilus_hypoxanthus_fuscicapillus (0.227-0.300)
349 Epinecrophylla_ornata_saturata (0.000-0.357)
769 Frederickena_fulva_ (0.333-0.500)

Consensus area 8 of 41 (from 1 areas; max. values)

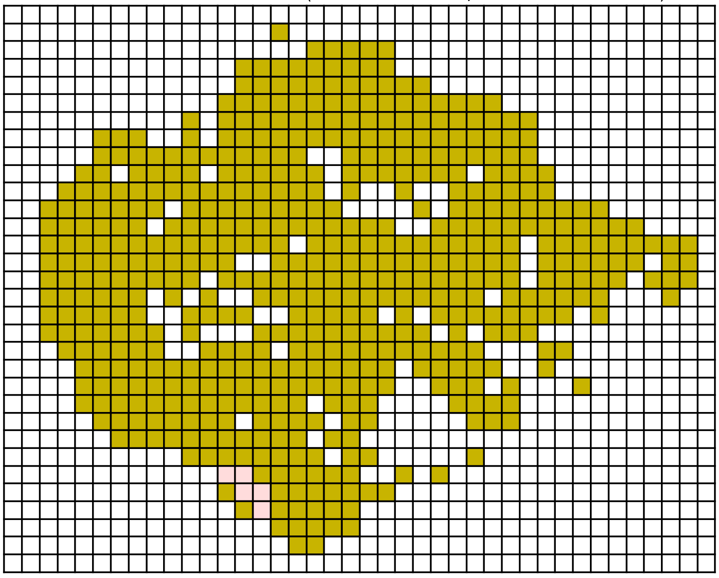

Just ground !
2.70014 - 2.9501 4

7 species give score :

26 Atlapetes_rufinucha_rufinucha(0.333)
52 Cranioleuca_albiceps_discolor(0.429)
575 Tangara_punctata_punctulata(0.364)
615 Aglaeactis_pamela_(0.286)
820 Hemitriccus_spodiops_(0.556)
862 Leptasthenura_yanacensis_(0.400)
985 Phyllomyias_weedeni_(0.333)

Consensus area 9 of 41 (from 2 areas; max. values)

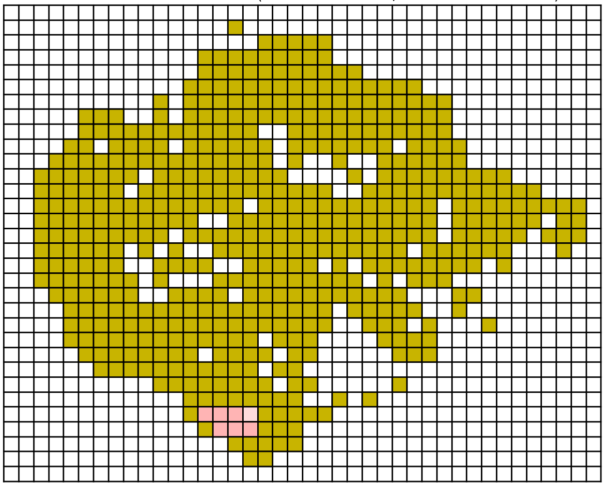

Just ground !
3.33395 - 3.5839 5
3.58395 - 3.8339 5

8 species give score :

9 Asthenes_harterti_bejaranoi(0.429-0.667)
24 Atlapetes_rufinucha_carrikeri(0.308-0.600)
52 Cranioleuca_albiceps_discolor(0.667-0.800)
221 Hapalopsittaca_melanotis_melanotis(0.167-0.182)
282 Metallura_aeneocauda_malagae(0.250-0.286)
615 Aglaeactis_pamela_(0.500-0.600)
626 Andigena_cucullata_(0.444-0.625)
707 Compsospiza_garleppi_(0.250-0.286)

Consensus area 10 of 41 (from 1 areas; max. values)

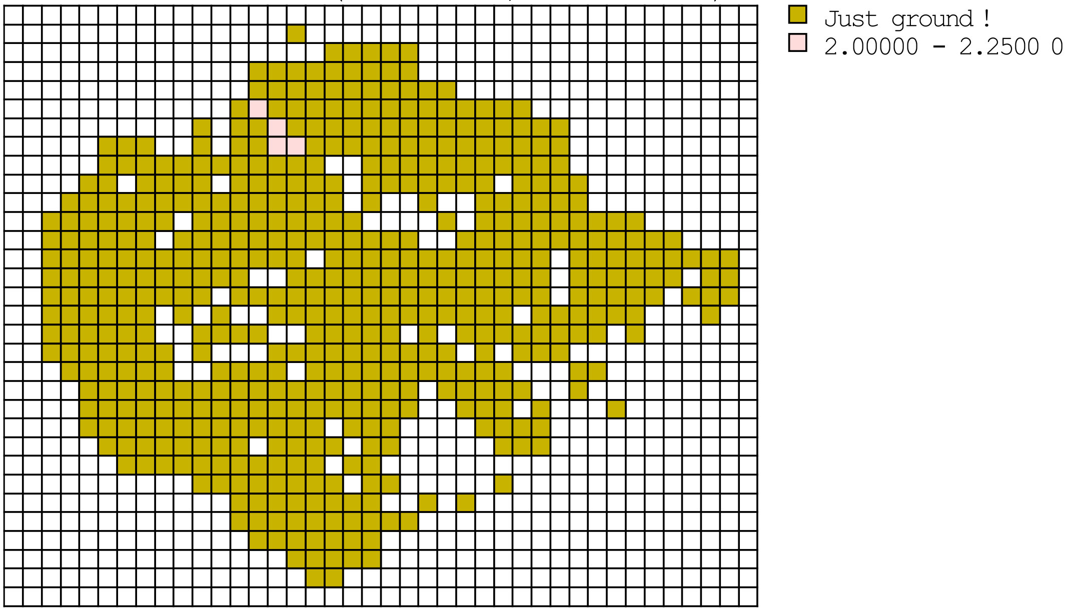

4 species give score :

123 Xenopipo_uniformis_duidae(0.500)
159 Elaenia_dayi_tyleri(0.500)
298 Myioborus_castaneocapilla_duidae(0.500)
471 Roraimia_adusta_duidae(0.500)

Consensus area 11 of 41 (from 2 areas; max. values)

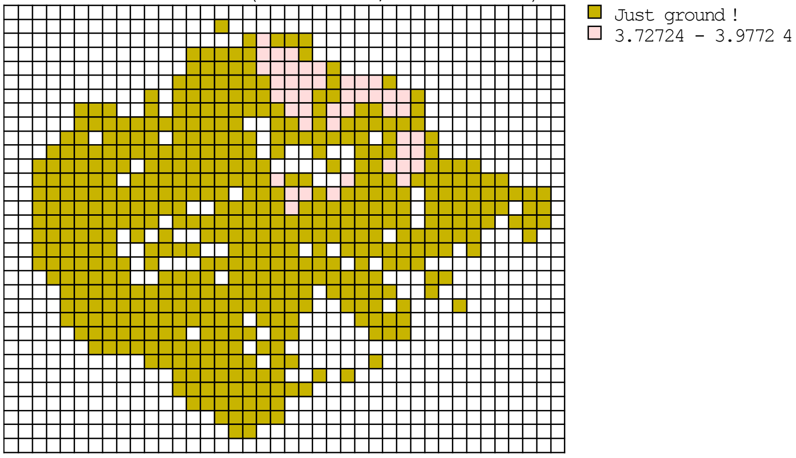

8 species give score :

112 Campylorhamphus_procurvoides_procurvoides(0.521-0.563)
188 Gymnopithys_rufigula_rufigula(0.530-0.561)
308 Myrmeciza_ferruginea_ferruginea(0.409-0.469)
378 Pithys_albifrons_albifrons(0.731-0.769)
677 Capito_niger_(0.484-0.484)
757 Epinecrophylla_gutturalis_(0.536-0.571)
1050 Selenidera_piperivora_(0.000-0.290)
1087 Tyranneutes_virescens_(0.310-0.333)

Consensus area 12 of 41 (from 1 areas; max. values)

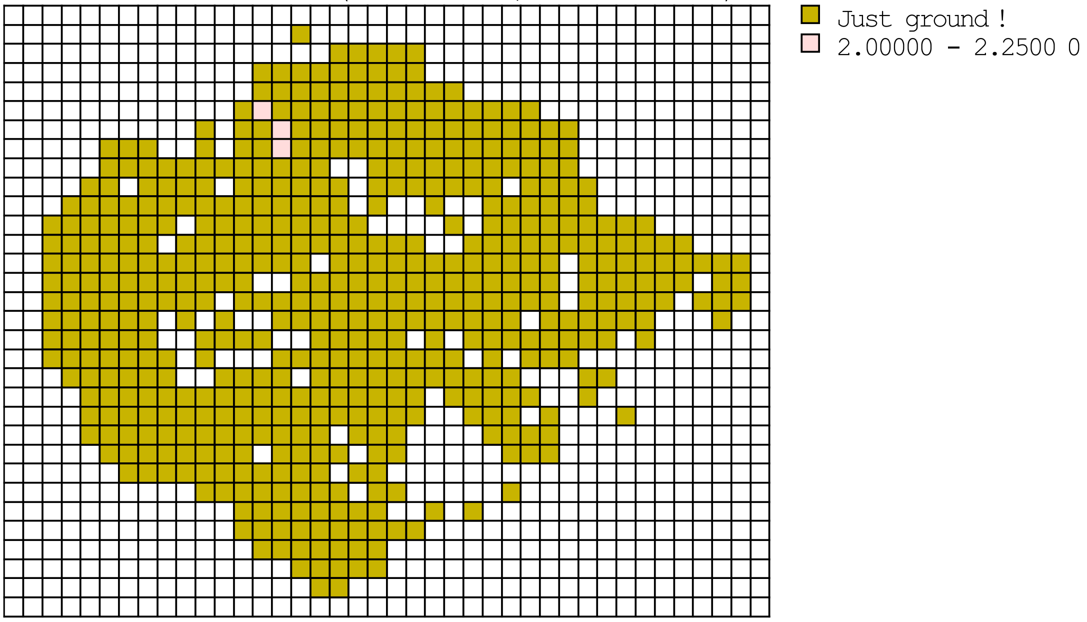

3 species give score :
123 Xenopipo_uniformis_ duidae(0.667)
159 Elaenia_dayi_tyleri(0.667)
471 Roraimia_adusta_duidae(0.667)

Consensus area 13 of 41 (from 1 areas; max. values)

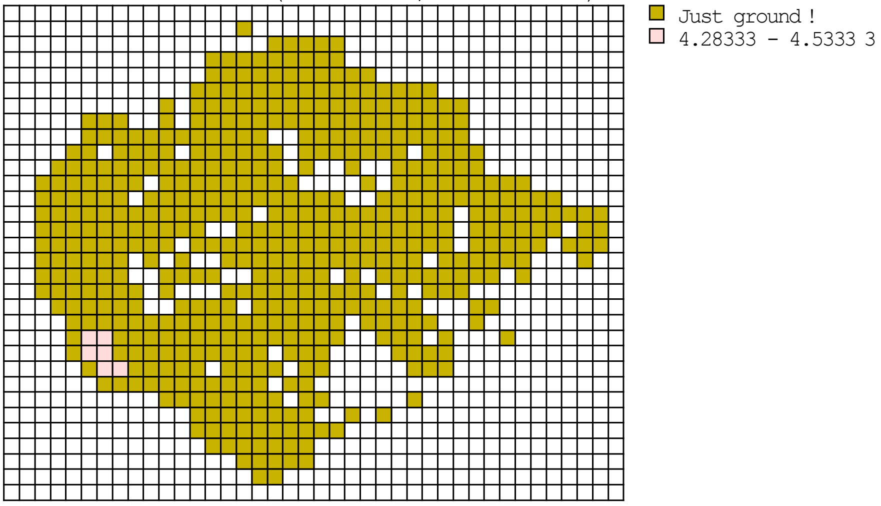

10 species give score :
42 Cranioleuca_albicapilla_albicapilla(0.250)
243 Hypocnemis_subflava_subflava(0.300)
323 Myrmoborus_leucophrys_koenigorum(1.000)
359 Epinecrophylla_spodionota_sororia(0.250)
531 Pheugopedius_coraya_cantator(0.833)
618 Amazilia_viridicauda_(0.167)
646 Atlapetes_melanopsis_(0.250)
825 Herpsilochmus_motacilloides_(0.400)
1008 Pipreola_pulchra_(0.500)
1071 Tangara_chilensis_(0.333)

Consensus area 14 of 41 (from 3 areas; max. values)

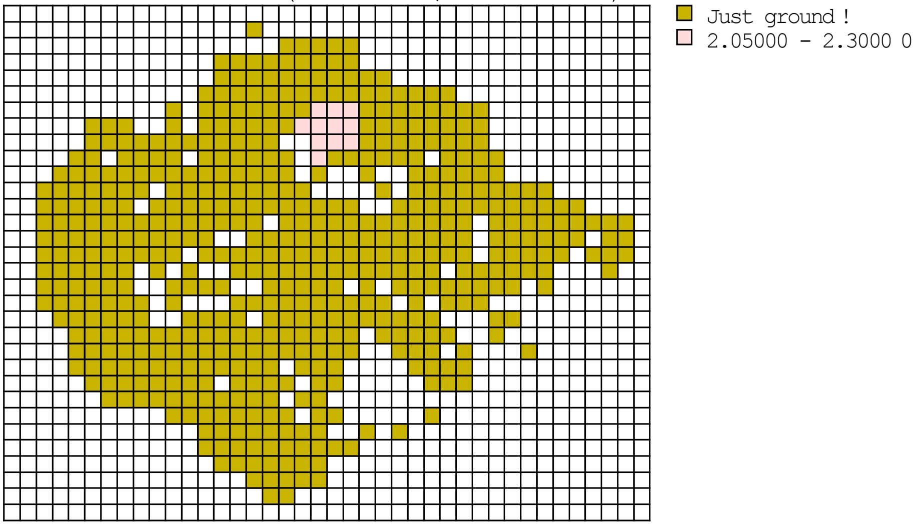

4 species give score :

78 Celeus_elegans_hellmayri (0.308-0.400)
84 Campylorhynchus_griseus_griseus (0.500-0.909)
686 Cercomacra_carbonaria_(0.308-0.400)
1056 Sporophila_intermedia_(0.545-0.750)

Consensus area 15 of 41 (from 1 areas; max. values)

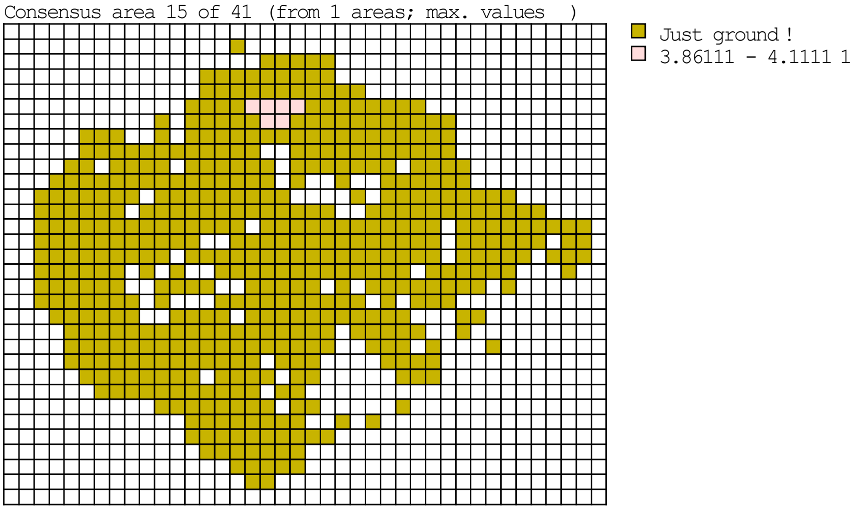

12 species give score :

18 Atlapetes_personatus_collaris (0.250)
73 Cranioleuca_demissa_demissa (0.111)
157 Elaenia_dayi_auyantepui (0.333)
302 Microbates_collaris_paraguensis (0.667)
346 Mitrospingus_oleagineus_obscuripectus (0.300)
400 Phylloscartes_chapmani_chapmani (0.500)
411 Pyrrhura_egregia_obscura (0.200)
470 Roraimia_adusta_adusta (0.250)
500 Synallaxis_macconnelli_yavii (0.167)
549 Thamnophilus_insignis_insignis (0.500)
731 Crypturellus_ptaritepui_(0.333)
1053 Setopagis_whitelyi_(0.250)

Consensus area 16 of 41 (from 2 areas; max. values)

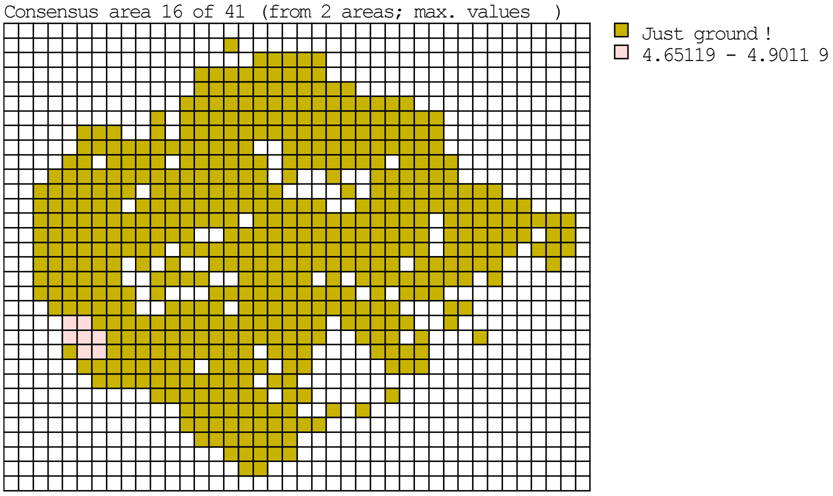

11 species give score :
42 Cranioleuca_albicapilla_albicapilla(0.143-0.231) 106 Cercomacra_nigrescens_notata(0.462-0.500)
243 Hypocnemis_subflava_subflava(0.714-0.833) 323 Myrmoborus_leucophrys_koenigorum(0.364-0.400)
359 Epinecrophylla_spodionota_sororia(0.222-0.250) 430 Picumnus_lafresnayi_taczanowskii(0.556-0.625)
531 Pheugopedius_coraya_cantator(0.444-0.500) 745 Doliornis_sclateri_(0.250-0.364)
788 Grallaria_capitalis_(0.500-0.714) 825 Herpsilochmus_motacilloides_(0.400-0.556)
1008 Pipreola_pulchra_(0.222-0.250)

Consensus area 17 of 41 (from 2 areas; max. values)

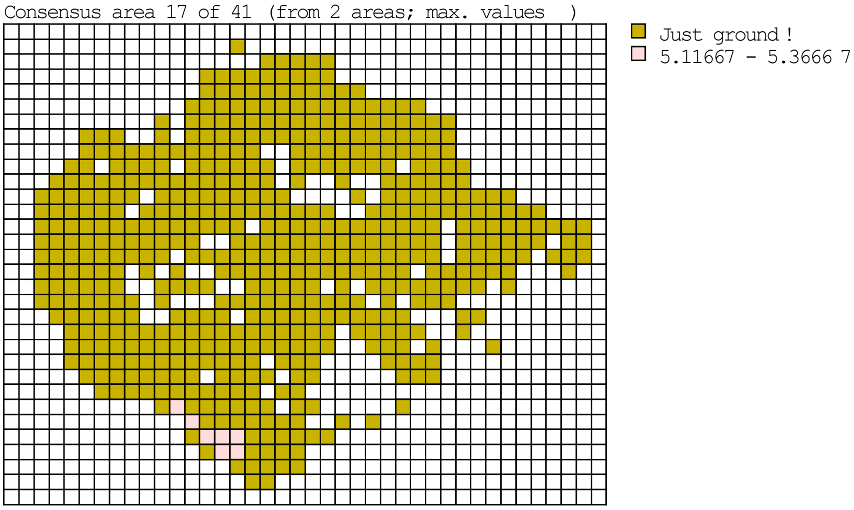

13 species give score :
29 Asthenes_urubambensis_urubambensis(0.500) 55 Crypturellus_atrocapillus_garleppi(0.250)
82 Cinnycerthia_fulva_gravesi(0.400) 415 Pipreola_intermedia_signata(0.400-0.625)
575 Tangara_punctata_punctulata(0.333-0.500) 640 Asthenes_maculicauda_(0.333)
726 Creurgops_dentatus_(0.250) 808 Hemispingus_calophrys_(0.250)
811 Hemispingus_trifasciatus_(0.400) 820 Hemitriccus_spodiops_(0.500)
868 Lipaugus_uropygialis_(0.500) 901 Myiophobus_inornatus_(0.667)
916 Myrmotherula_grisea_(0.167)

Consensus area 18 of 41 (from 1 areas; max. values)

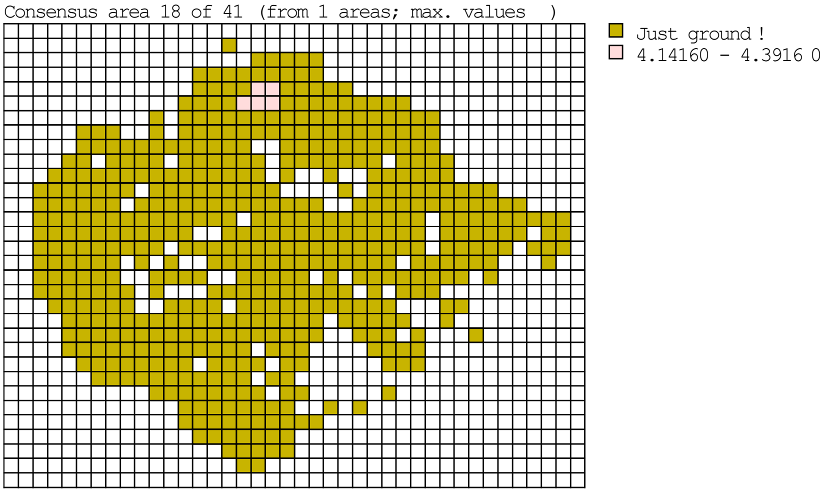

12 species give score :

18 Atlapetes_personatus_collaris(0.600)
239 Hyloctistes_subulatus_lemae(0.118)
292 Myioborus_castaneocapilla_castaneocapilla(0.364)
302 Microbates_collaris_paraguensis(0.222)
346 Mitrospingus_oleagineus_obscuripectus(0.333)
356 Myrmothera_simplex_guaiquinimae(0.400)
365 Microcerculus_ustulatus_lunatipectus(0.286)
424 Schistocichla_leucostigma_obscura(0.333)
472 Roraimia_adusta_mayri(0.400)
549 Thamnophilus_insignis_insignis(0.286)
731 Crypturellus_ptaritepui_(0.400)
896 Myioborus_cardonai_(0.400)

Consensus area 19 of 41 (from 1 areas; max. values)

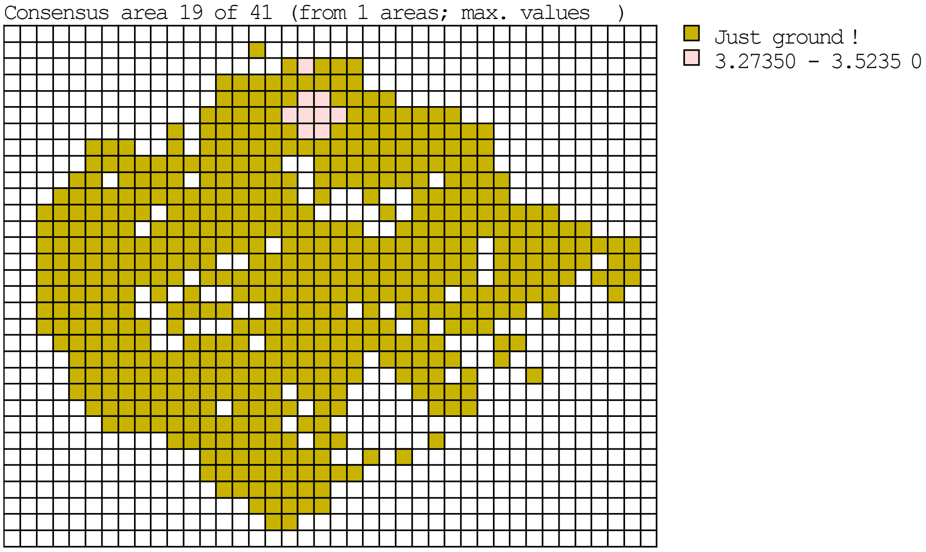

5 species give score :

21 Atlapetes_personatus_paraquensis(0.385)
358 Myrmothera_simplex_simplex(0.889)
529 Pheugopedius_coraya_barrowcloughianus(0.333)
867 Lipaugus_streptophorus_(1.000)
1012 Poecilotriccus_russatus_(0.667)

Consensus area 20 of 41 (from 1 areas; max. values)

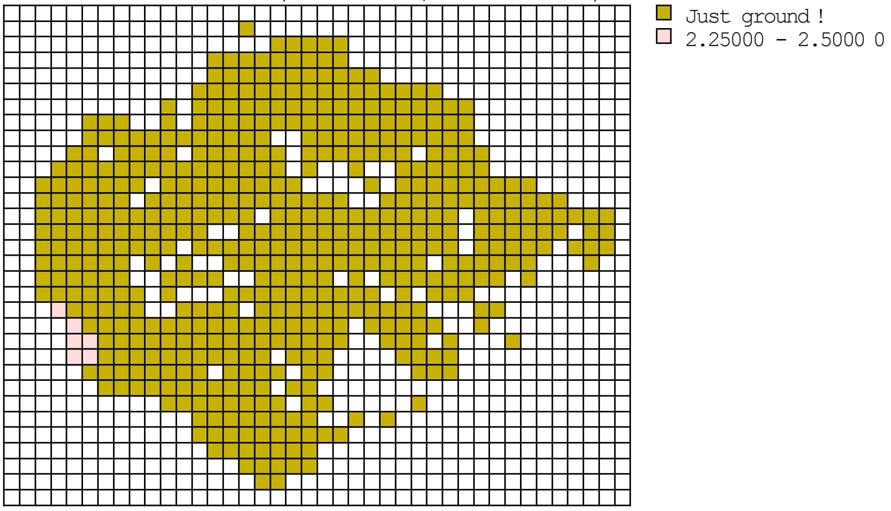

5 species give score :
7 Aglaeactis_castelnaudii_castelnaudii(0.333) 243 Hypocnemis_subflava_subflava(0.167)
745 Doliornis_sclateri_(1.000) 788 Grallaria_capitalis_(0.500)
932 Nephelornis_oneilli_(0.250)

Consensus area 21 of 41 (from 1 areas; max. values)

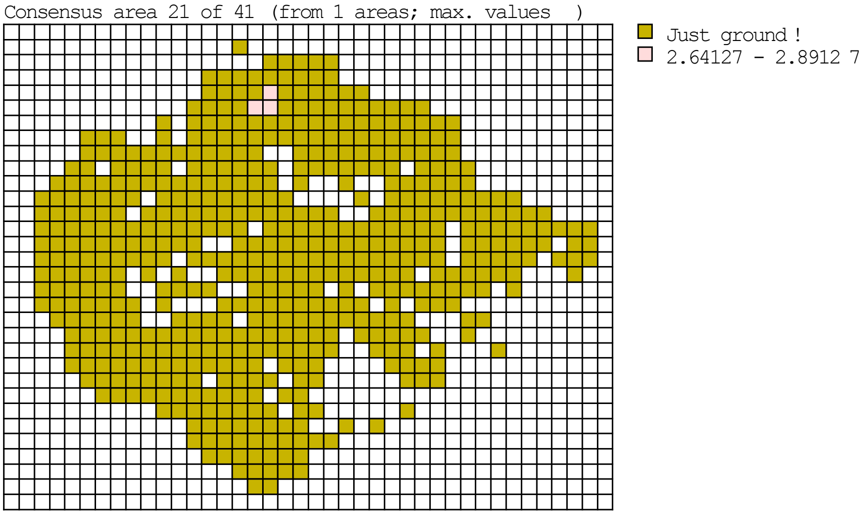

6 species give score :
18 Atlapetes_personatus_collaris(0.400) 302 Microbates_collaris_paraguensis(0.286)
356 Myrmothera_simplex_guaiquinimae(0.667) 365 Microcerculus_ustulatus_lunatipectus(0.400)
424 Schistocichla_leucostigma_obscura(0.222) 896 Myioborus_cardonai_(0.667)

Consensus area 22 of 41 (from 2 areas; max. values)

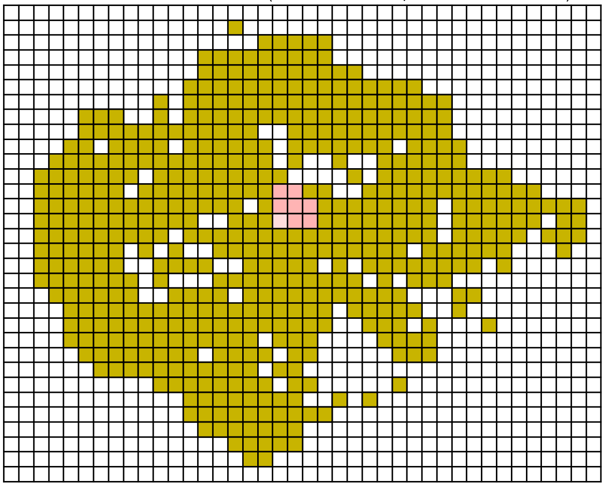

Just ground !
2.25000 - 2.5000 0
2.50000 - 2.7500 0

6 species give score :

38 Brotogeris_chrysoptera_tenuifrons(0.308-0.500)
56 Capito_auratus_hypochondriacus(0.500-0.667)
143 Dendrocincla_merula_obidensis(0.000-0.444)
331 Myrmoborus_lugubris_stictopterus(0.429-0.500)
453 Percnostola_rufifrons_subcristata(0.444-0.500)
1059 Stigmatura_napensis_(0.222-0.250)

Consensus area 23 of 41 (from 1 areas; max. values)

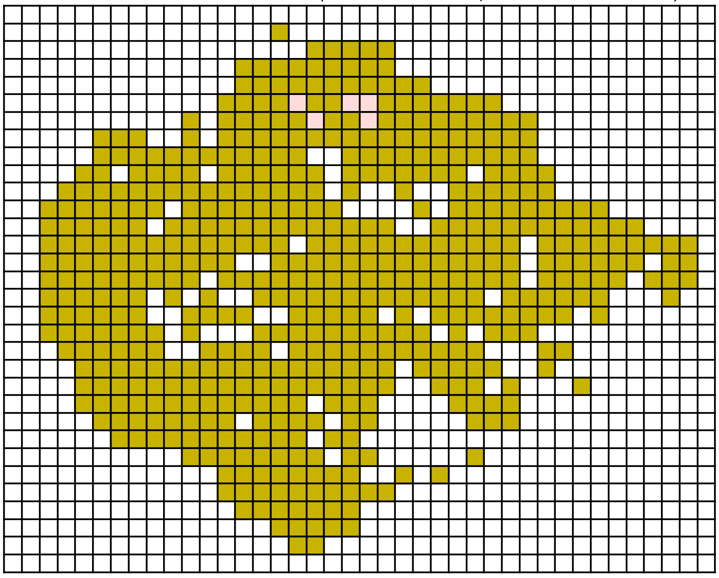

Just ground !
2.13333 - 2.3833 3

4 species give score :

411 Pyrrhura_egregia_obscura(0.800)
470 Roraimia_adusta_adusta(0.600)
599 Tangara_xanthogastra_phelpsi(0.133)
1053 Setopagis_whitelyi_(0.600)

Consensus area 24 of 41 (from 1 areas; max. values)

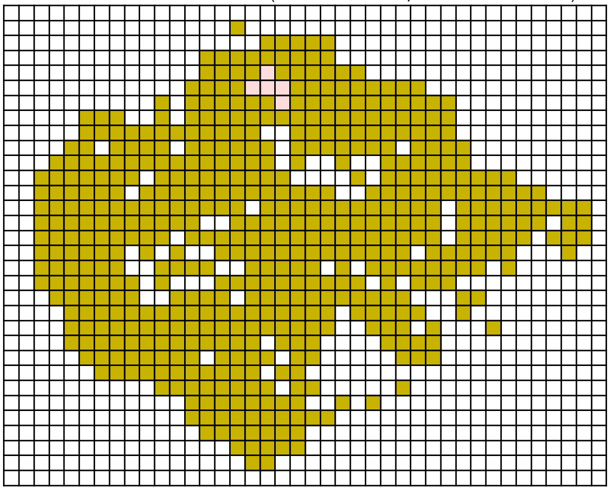

Just ground !
4.02720 - 4.2772 0

13 species give score :

18 Atlapetes_personatus_collaris(0.600) 157 Elaenia_dayi_auyantepui(0.400)
239 Hyloctistes_subulatus_lemae(0.118) 292 Myioborus_castaneocapilla_castaneocapilla(0.231)
302 Microbates_collaris_paraguensis(0.429) 346 Mitrospingus_oleagineus_obscuripectus(0.182)
356 Myrmothera_simplex_guaiquinimae(0.400) 365 Microcerculus_ustulatus_lunatipectus(0.286)
400 Phylloscartes_chapmani_chapmani(0.333) 424 Schistocichla_leucostigma_obscura(0.182)
500 Synallaxis_macconnelli_yavii(0.182) 549 Thamnophilus_insignis_insignis(0.286)
731 Crypturellus_ptaritepui_(0.400)

Consensus area 25 of 41 (from 1 areas; max. values)

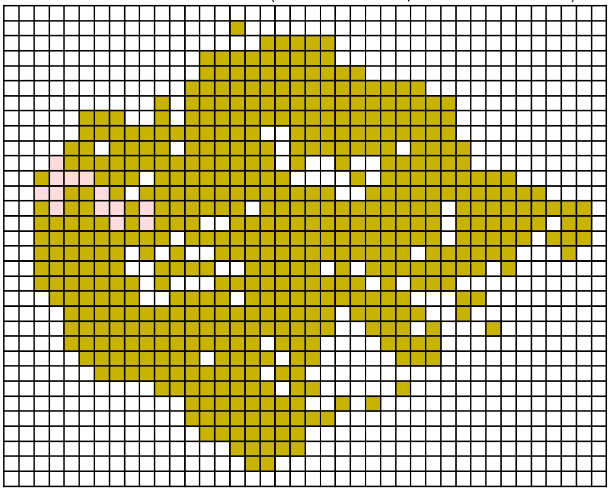

Just ground !
2.34842 - 2.5984 2

4 species give score :

212 Hylophilus_hypoxanthus_fuscicapillus(0.923) 537 Pheugopedius_coraya_griseipectus(0.440)
769 Frederickena_fulva_(0.462) 823 Herpsilochmus_dugandi_(0.524)

Consensus area 26 of 41 (from 1 areas; max. values)

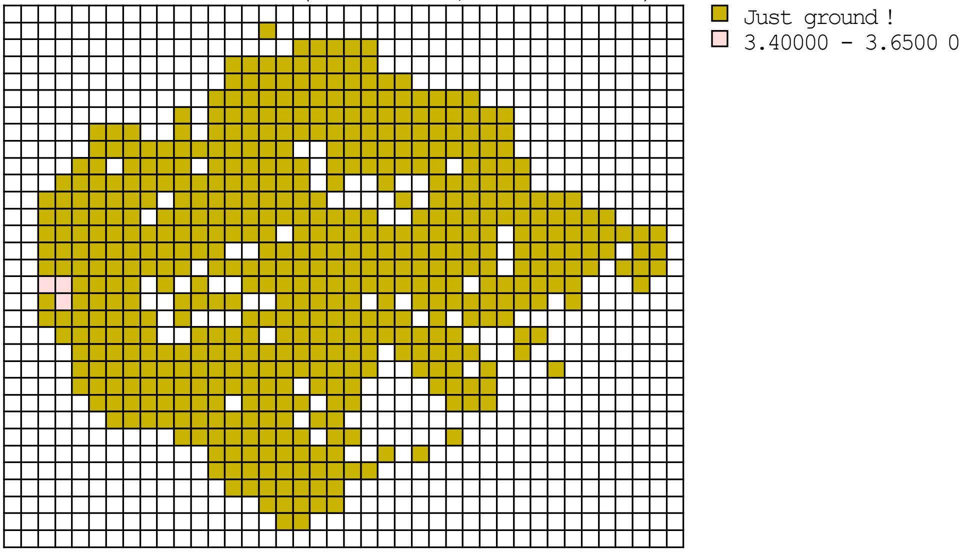

5 species give score :

36 Brotogeris_cyanoptera_gustavi (1.000)
534 Tangara_chilensis_chlorocorys (0.400)
812 Hemitriccus_cinnamomeipectus_(0.667)
291 Myrmeciza_castanea_castanea (0.667)
795 Grallaricula_ochraceifrons_(0.667)

Consensus area 27 of 41 (from 1 areas; max. values)

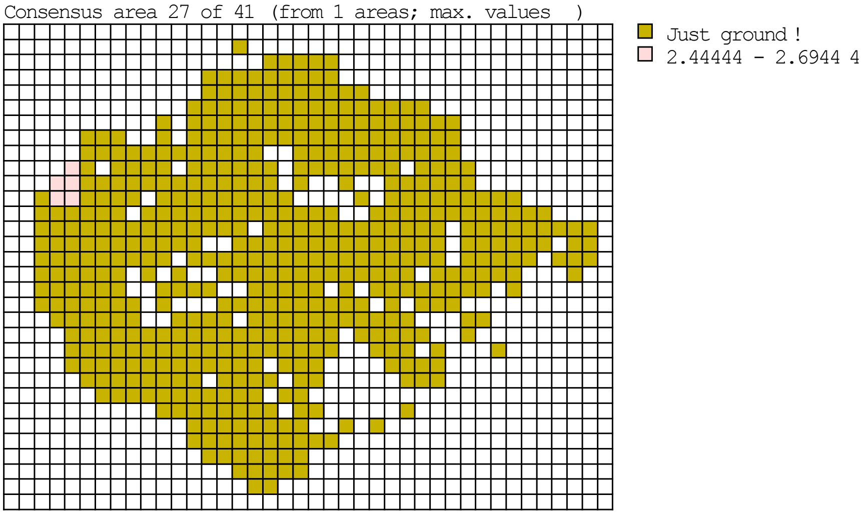

4 species give score :

6 Anurolimnas_castaneiceps_coccineipes (0.444)
206 Hylopezus_fulviventris_caquetae (0.400)
62 Conopophaga_aurita_occidentalis (1.000)
296 Microbates_collaris_colombianus (0.600)

Consensus area 28 of 41 (from 1 areas; max. values)

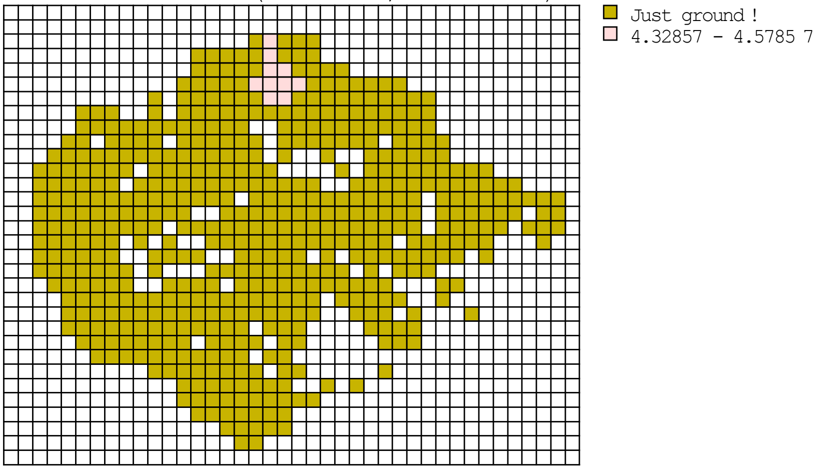

7 species give score :
21 Atlapetes_personatus_paraquensis(0.500)
358 Myrmothera_simplex_simplex(0.800)
670 Campylopterus_hyperythrus_(0.600)
1012 Poecilotriccus_russatus_(0.600)
292 Myioborus_castaneocapilla_castaneocapilla(0.500)
529 Pheugopedius_coraya_barrowcloughianus(0.429)
867 Lipaugus_streptophorus_(0.900)

Consensus area 29 of 41 (from 1 areas; max. values)

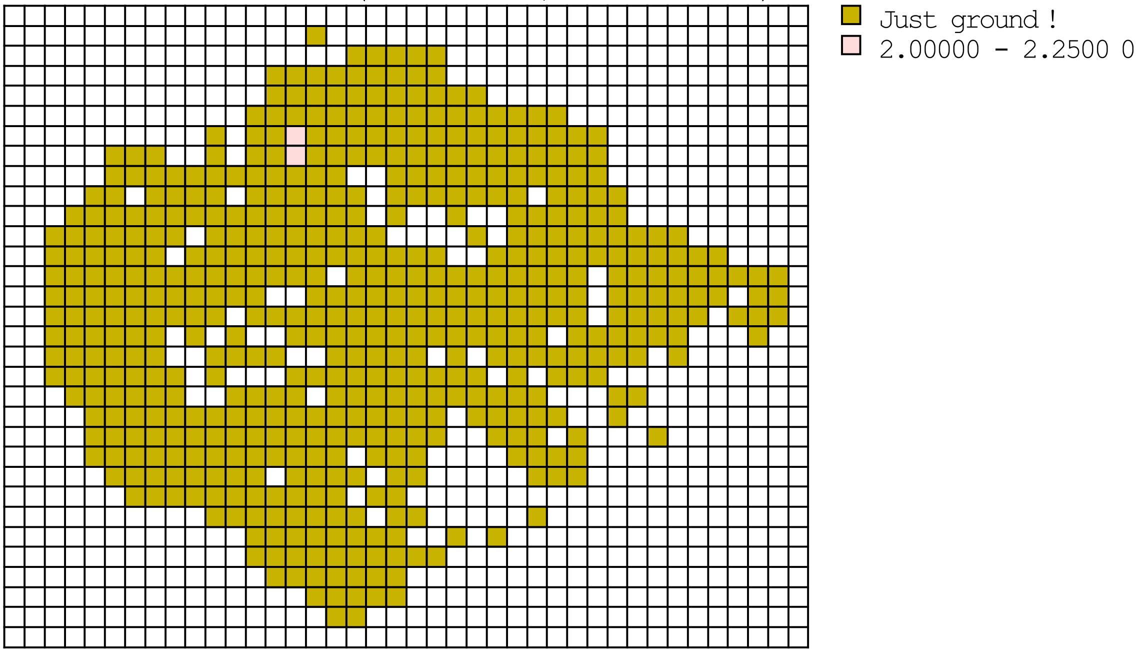

2 species give score :
159 Elaenia_dayi_tyleri(1.000)
471 Roraimia_adusta_duidae(1.000)

Consensus area 30 of 41 (from 1 areas; max. values)

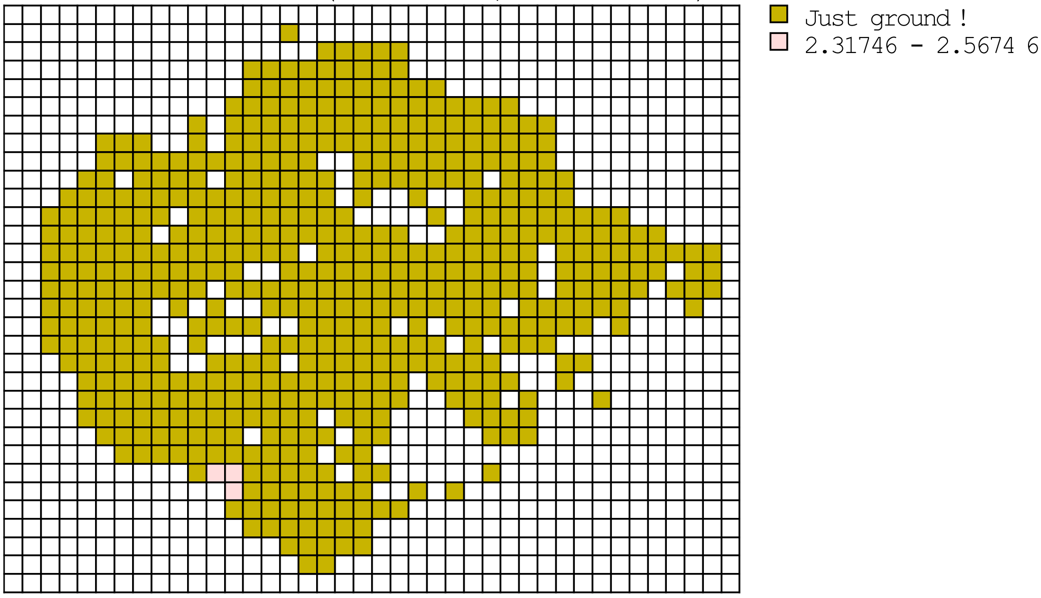

4 species give score :

26 Atlapetes_rufinucha_rufinucha(0.222)
977 Phibalura_boliviana_(1.000)
985 Phyllomyias_weedeni_(0.429)
1043 Schistocichla_brunneiceps_(0.667)

Consensus area 31 of 41 (from 1 areas; max. values)

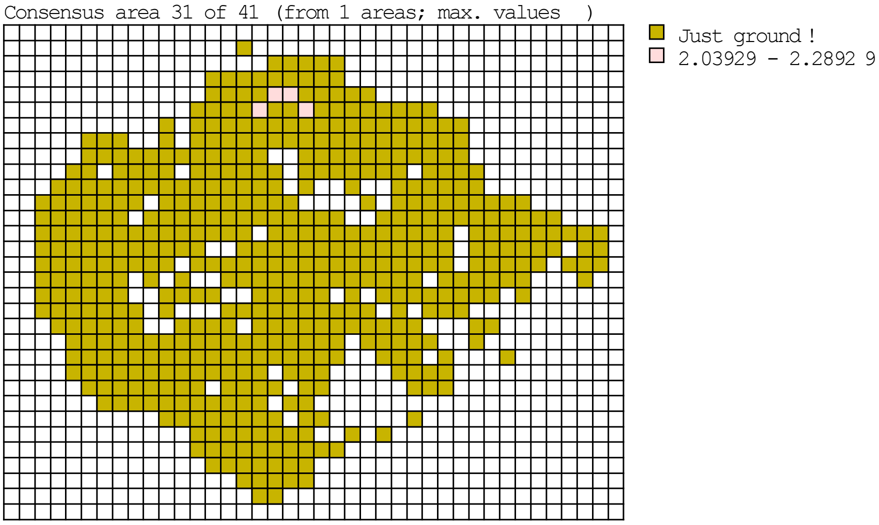

8 species give score :

73 Cranioleuca_demissa_demissa(0.214)
239 Hyloctistes_subulatus_lemae(0.125)
292 Myioborus_castaneocapilla_castaneocapilla(0.250)
411 Pyrrhura_egregia_obscura(0.250)
424 Schistocichla_leucostigma_obscura(0.200)
472 Roraimia_adusta_mayri(0.500)
581 Troglodytes_rufulus_fulvigularis(0.167)
1053 Setopagis_whitelyi_(0.333)

Consensus area 32 of 41 (from 1 areas; max. values)

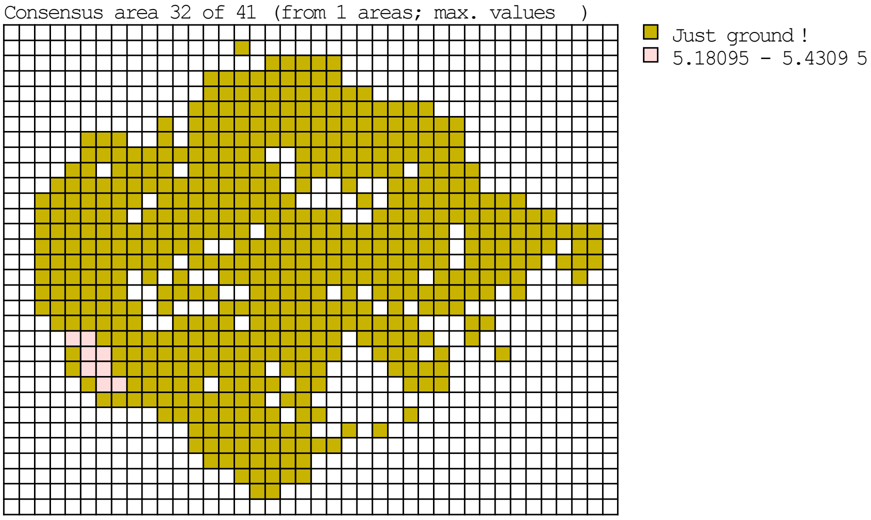

11 species give score :
42 Cranioleuca_albicapilla_albicapilla(0.214)
106 Cercomacra_nigrescens_notata(0.800)
243 Hypocnemis_subflava_subflava(0.625)
323 Myrmoborus_leucophrys_koenigorum(0.750)
359 Epinecrophylla_spodionota_sororia(0.375)
430 Picumnus_lafresnayi_taczanowskii(0.500)
475 Rhegmatorhina_melanosticta_brunneiceps(0.167)
531 Pheugopedius_coraya_cantator(0.625)
618 Amazilia_viridicauda_(0.250)
825 Herpsilochmus_motacilloides_(0.500)
1008 Pipreola_pulchra_(0.375)

Consensus area 33 of 41 (from 1 areas; max. values)

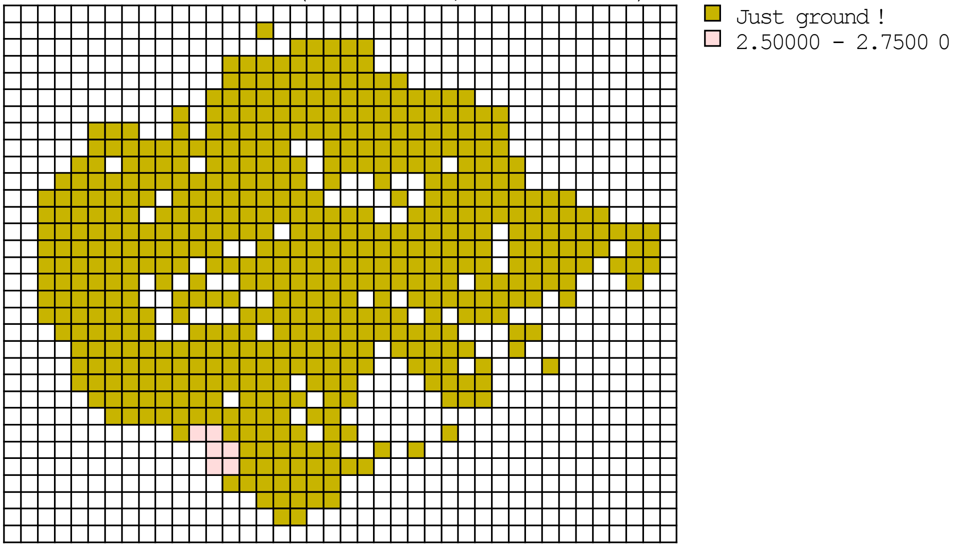

4 species give score :
0 Anairetes_alpinus_bolivianus(0.333)
26 Atlapetes_rufinucha_rufinucha(0.833)
977 Phibalura_boliviana_(0.500)
985 Phyllomyias_weedeni_(0.833)

Consensus area 34 of 41 (from 1 areas; max. values)

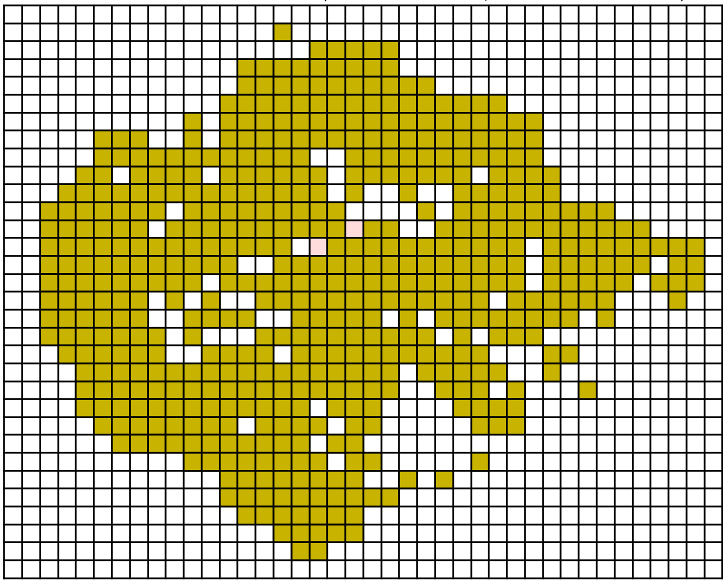

Just ground !
2.00000 - 2.2500 0

2 species give score :
426 Picumnus_lafresnayi_pusillus(1.000) 962 Percnostola_minor_(1.000)

Consensus area 35 of 41 (from 1 areas; max. values)

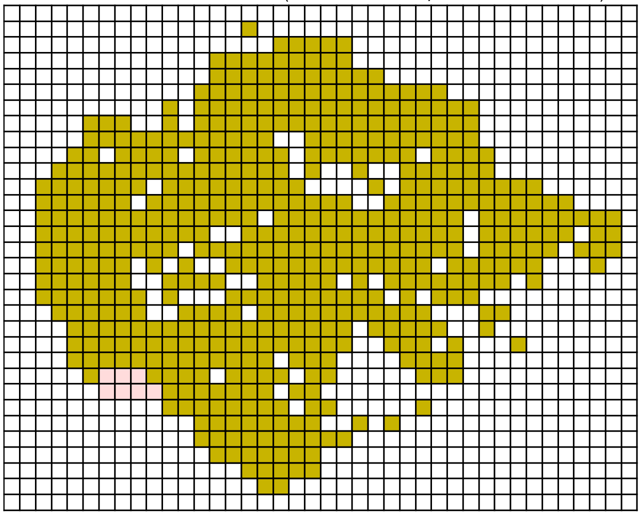

Just ground !
4.13542 - 4.3854 2

8 species give score :
8 Aglaeactis_castelnaudii_regalis(0.571) 44 Cranioleuca_albicapilla_albigula(0.571)
489 Synallaxis_gujanensis_canipileus(0.222) 641 Asthenes_ottonis_(1.000)
644 Atlapetes_canigenis_(0.462) 791 Grallaria_erythroleuca_(0.571)
950 Oreonympha_nobilis_(0.556) 981 Phlogophilus_harterti_(0.182)

Consensus area 36 of 41 (from 1 areas; max. values)

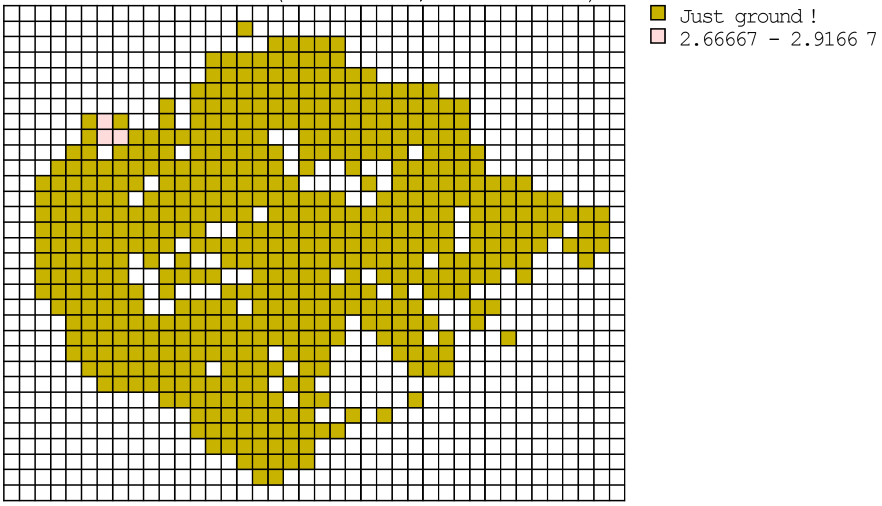

3 species give score :
31 Amazilia_viridigaster_viridigaster(1.000)
301 Myrmothera_campanisona_Mmodesta(1.000)
348 Epinecrophylla_ornata_ornata(0.667)

Consensus area 37 of 41 (from 1 areas; max. values)

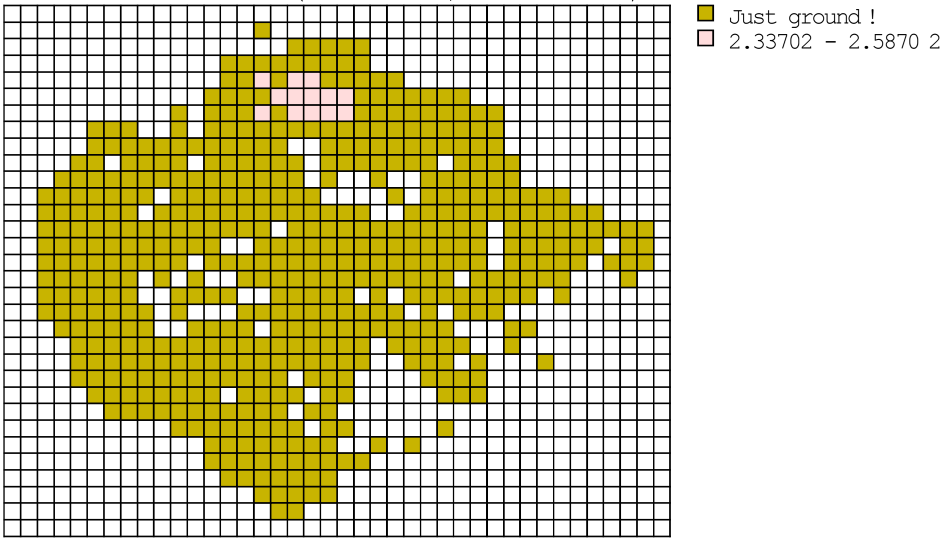

6 species give score :
73 Cranioleuca_demissa_demissa(0.467)
233 Herpsilochmus_roraimae_roraimae(0.579)
239 Hyloctistes_subulatus_lemae(0.353)
411 Pyrrhura_egregia_obscura(0.308)
599 Tangara_xanthogastra_phelpsi(0.400)
1053 Setopagis_whitelyi_(0.231)

Consensus area 38 of 41 (from 1 areas; max. values)

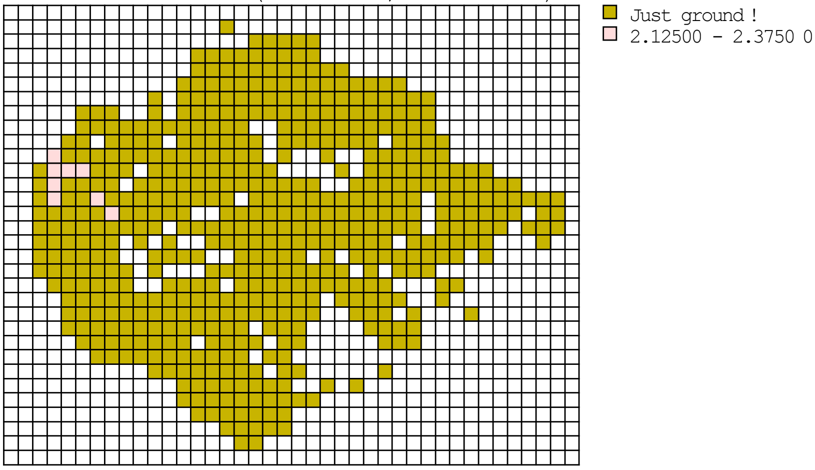

4 species give score :
28 Anabacerthia_ruficaudata_subflavescens(0.500)
208 Hylopezus_fulviventris_fulviventris(0.750)
349 Epinecrophylla_ornata_saturata(0.250)
571 Topaza_pyra_amaruni(0.625)

Consensus area 39 of 41 (from 1 areas; max. values)

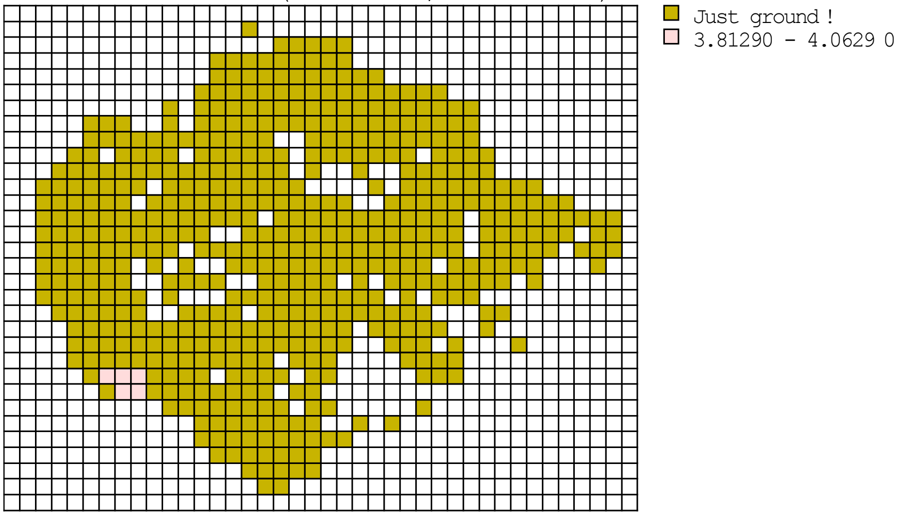

8 species give score :
8 Aglaeactis_castelnaudii_regalis(0.800)
44 Cranioleuca_albicapilla_albigula(0.800)
489 Synallaxis_gujanensis_canipileus(0.286)
641 Asthenes_ottonis_(0.556)
643 Asthenes_vilcabambae_(0.286)
644 Atlapetes_canigenis_(0.385)
791 Grallaria_erythroleuca_(0.429)
950 Oreonympha_nobilis_(0.273)

Consensus area 40 of 41 (from 1 areas; max. values)

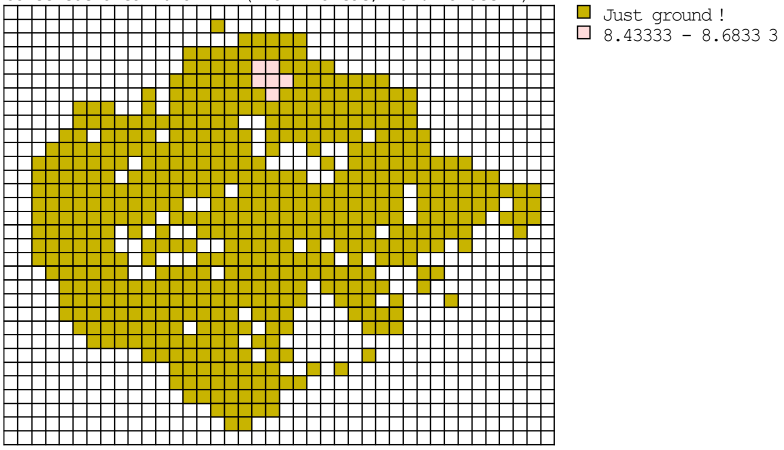

20 species give score :

23 Atlapetes_personatus_personatus(0.667)
141 Diglossa_major_major(0.500)
158 Elaenia_dayi_dayi(0.333)
289 Myrmotherula_behni_yavii(0.333)
292 Myioborus_castaneocapilla_castaneocapilla(0.333)
346 Mitrospingus_oleagineus_obscuripectus(0.500)
347 Mitrospingus_oleagineus_oleagineus(0.500)
351 Myiophobus_roraimae_roraimae(0.500)
357 Myrmothera_simplex_pacaraimae(0.333)
366 Microcerculus_ustulatus_obscurus(0.667)
367 Microcerculus_ustulatus_ustulatus(0.300)
408 Pyrrhura_egregia_egregia(0.667)
428 Schistocichla_leucostigma_saturata(0.333)
468 Pipreola_whitelyi_kathleenae(0.333)
469 Pipreola_whitelyi_whitelyi(0.333)
470 Roraimia_adusta_adusta(0.250)
542 Pheugopedius_coraya_obscurus(0.500)
549 Thamnophilus_insignis_insignis(0.250)
581 Troglodytes_rufulus_fulvigularis(0.400)
670 Campylopterus_hyperythrus_(0.400)

11 - AoE identified by PAE through species occurrence.

Map created in ArcGIS 10.1 (http://www.esri.com)

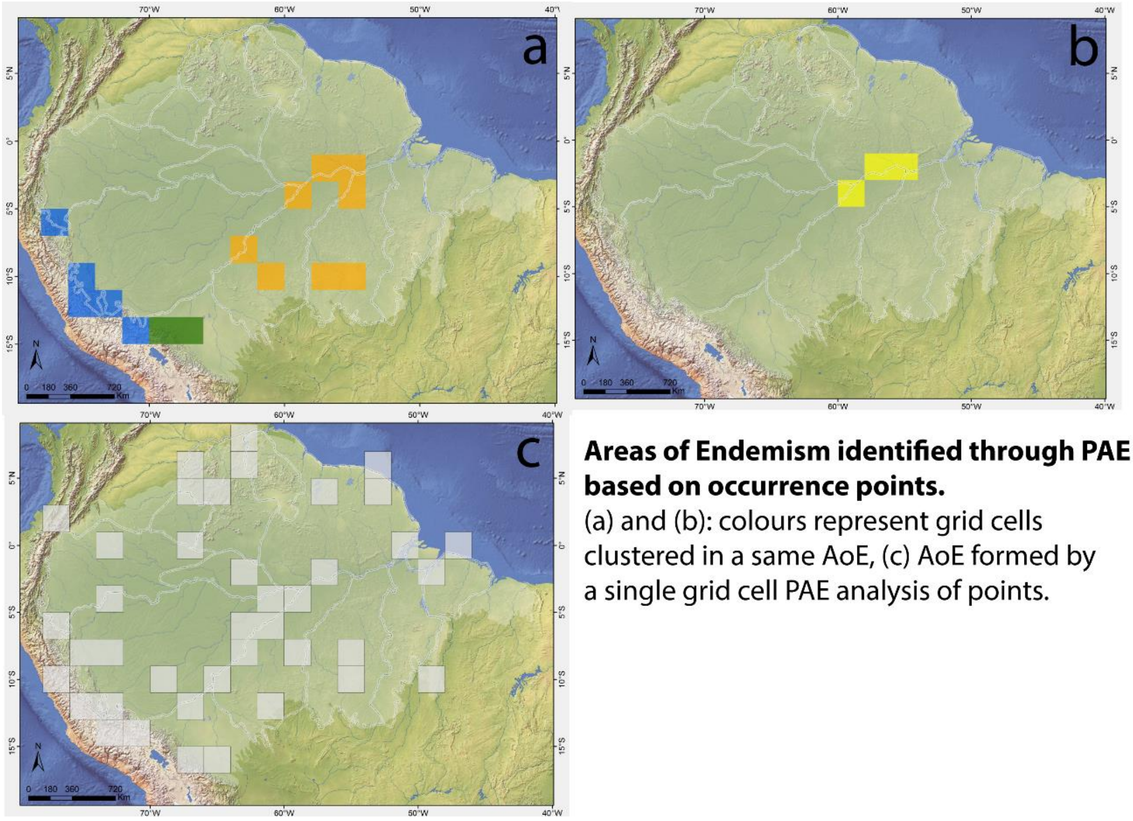

Areas of Endemism identified through PAE based on occurrence points.
(a) and (b): colours represent grid cells clustered in a same AoE, (c) AoE formed by a single grid cell PAE analysis of points.

12 - Consensus tree of PAE based on species occurrence.

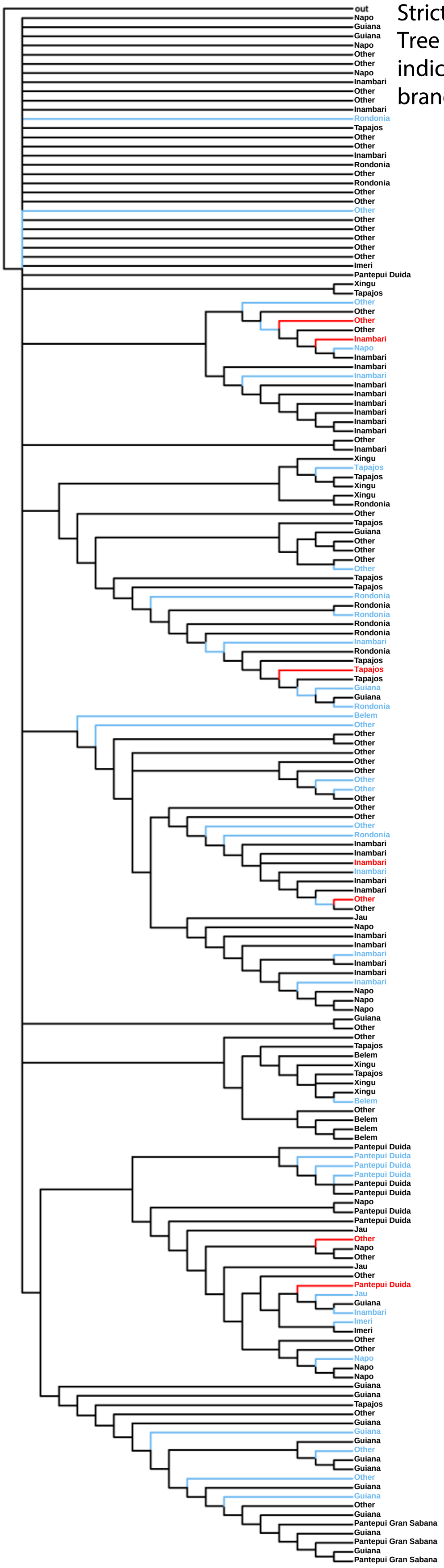

Strict consensus of PAE based on occurrence points. Tree with 7,112 steps. Branches and terminals in blue indicate the presence of one endemic species, and rec branches and terminals indicate two endemic species.

13 - Tree of constrained PAE based on species occurrence.

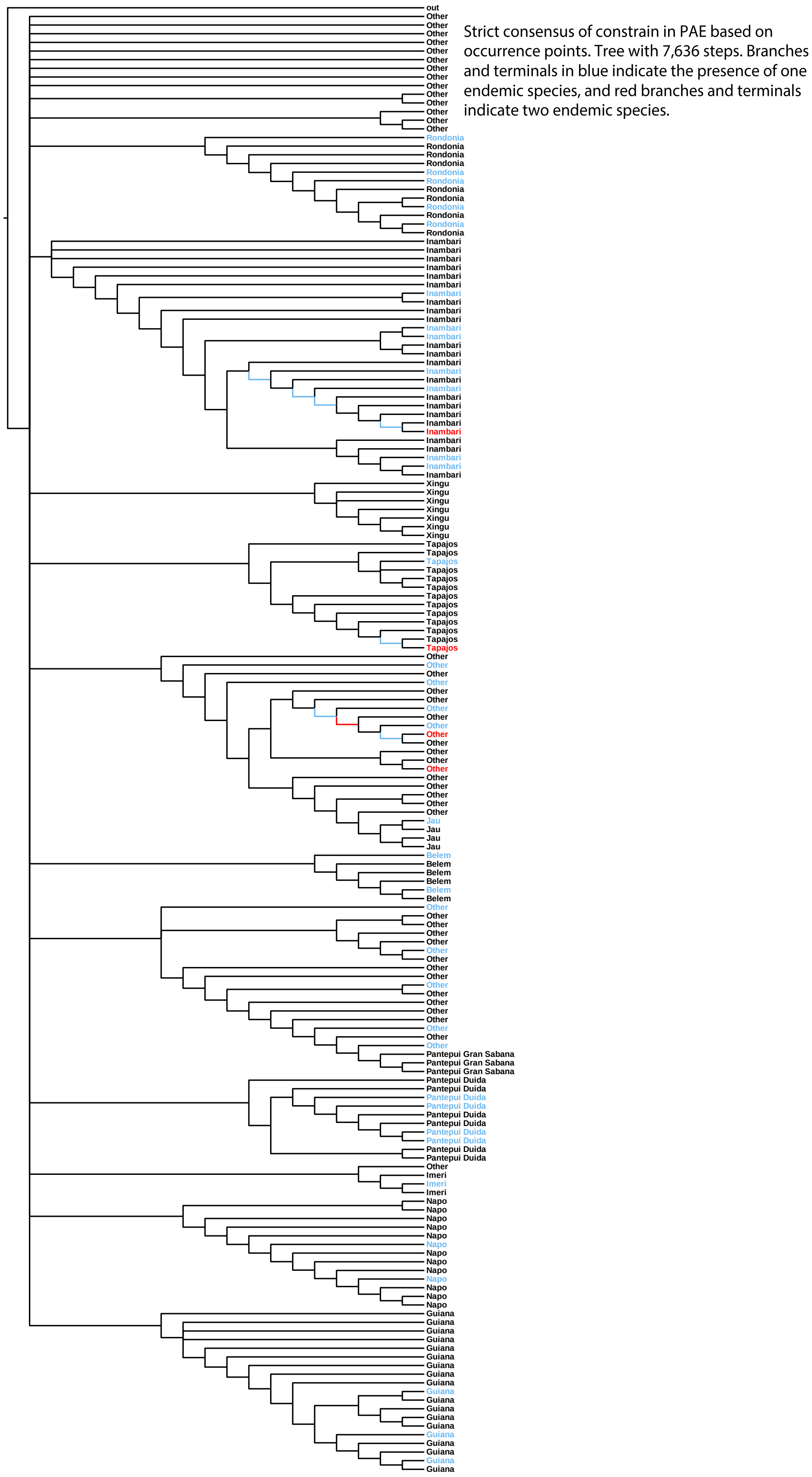

Strict consensus of constrain in PAE based on occurrence points. Tree with 7,636 steps. Branches and terminals in blue indicate the presence of one endemic species, and red branches and terminals indicate two endemic species.

14 - AoEs identified by PAE through subspecies occurrence.

Map created in ArcGIS 10.1 (http://www.esri.com)

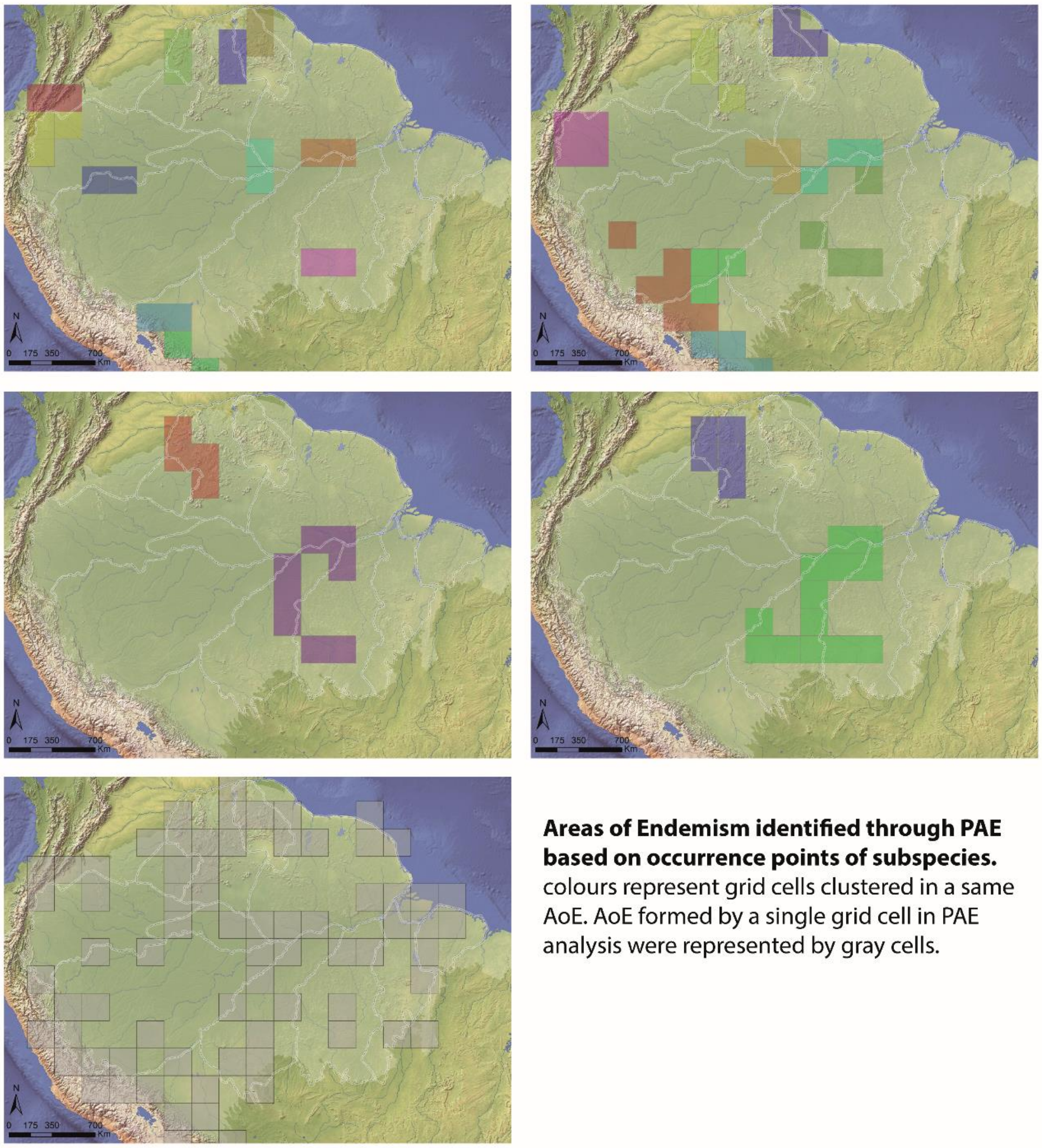

Areas of Endemism identified through PAE based on occurrence points of subspecies. colours represent grid cells clustered in a same AoE. AoE formed by a single grid cell in PAE analysis were represented by gray cells.

15 - Consensus tree of PAE based on subspecies occurrence.

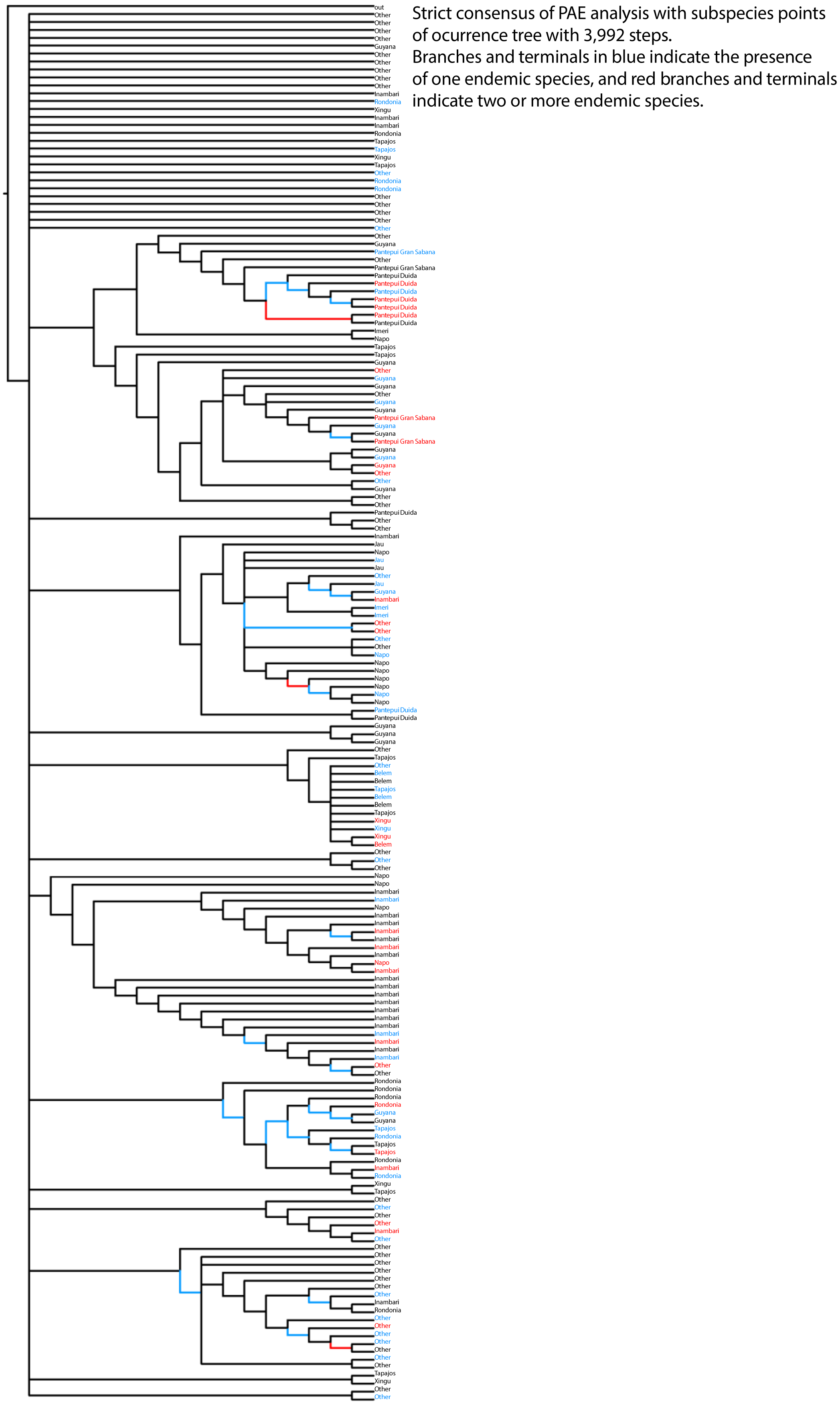

Strict consensus of PAE analysis with subspecies points of ocurrence tree with 3,992 steps.
Branches and terminals in blue indicate the presence of one endemic species, and red branches and terminals indicate two or more endemic species.

16 - Tree of constrained PAE based on subspecies occurrence.

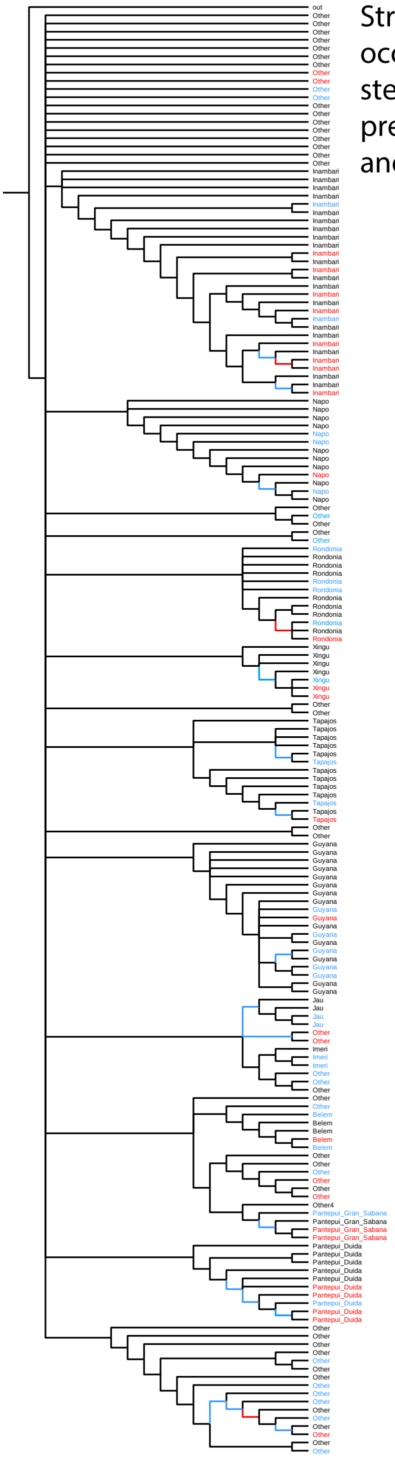

Strict consensus of constrain in PAE based on occurrence points of subspecies. Tree with 8,904 steps. Branches and terminals in blue indicate the presence of one endemic species, and red branches and terminals indicate two or more endemic species.

17 - Autocorrelogram of Moran I analysis of three axis of NMDS analysis.

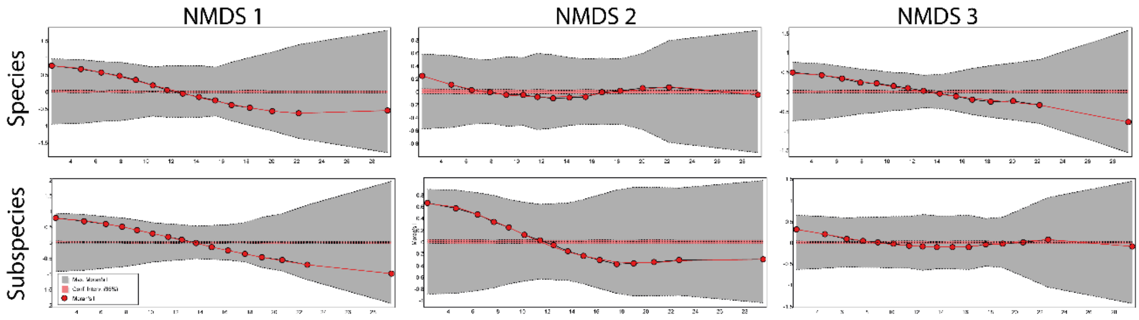

18 - Most relevant breaks in species composition obtained by Monmonier's Algorithm

Map created in ArcGIS 10.1 (http://www.esri.com)

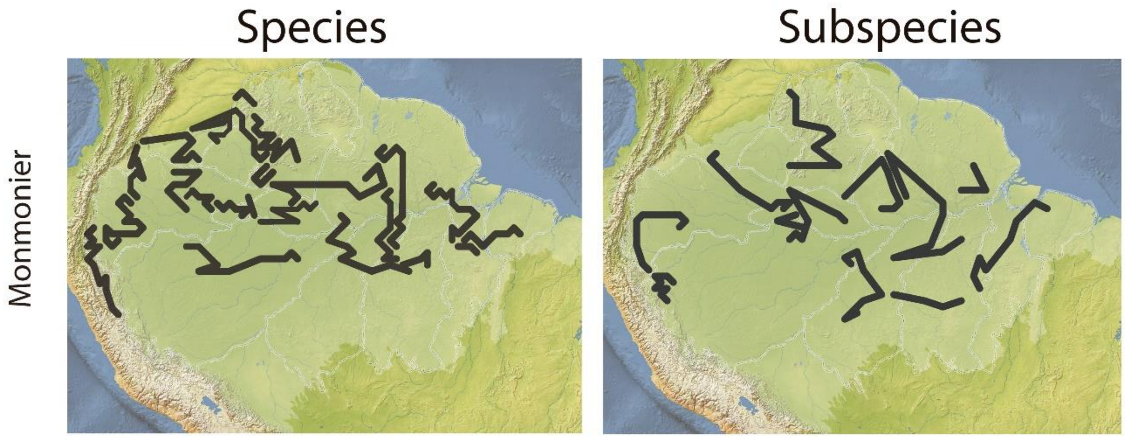

19 - Interpolation of the three axes of NMDS based on species occurrence, subspecies occurrence. Numbers indicate correlation between maps.

Map created in ArcGIS 10.1 (http://www.esri.com)

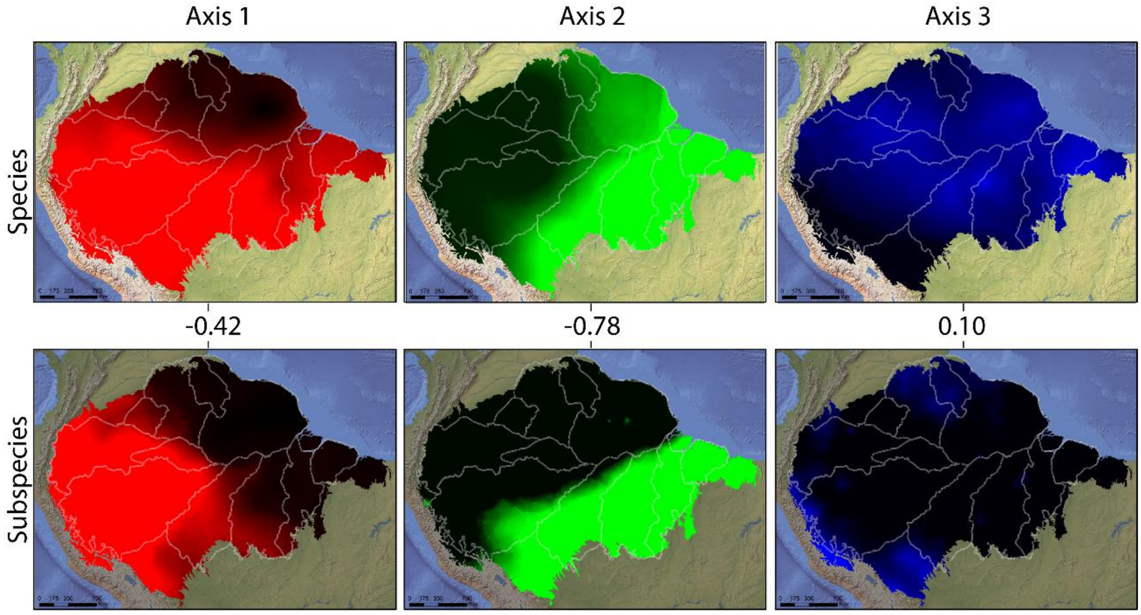

20 - Unsupervised classification of the spatial variation in species composition. Colours represent different groups in the classification. Each line represents a classification in the number of classes indicated at left.

Map created in ArcGIS 10.1 (http://www.esri.com)

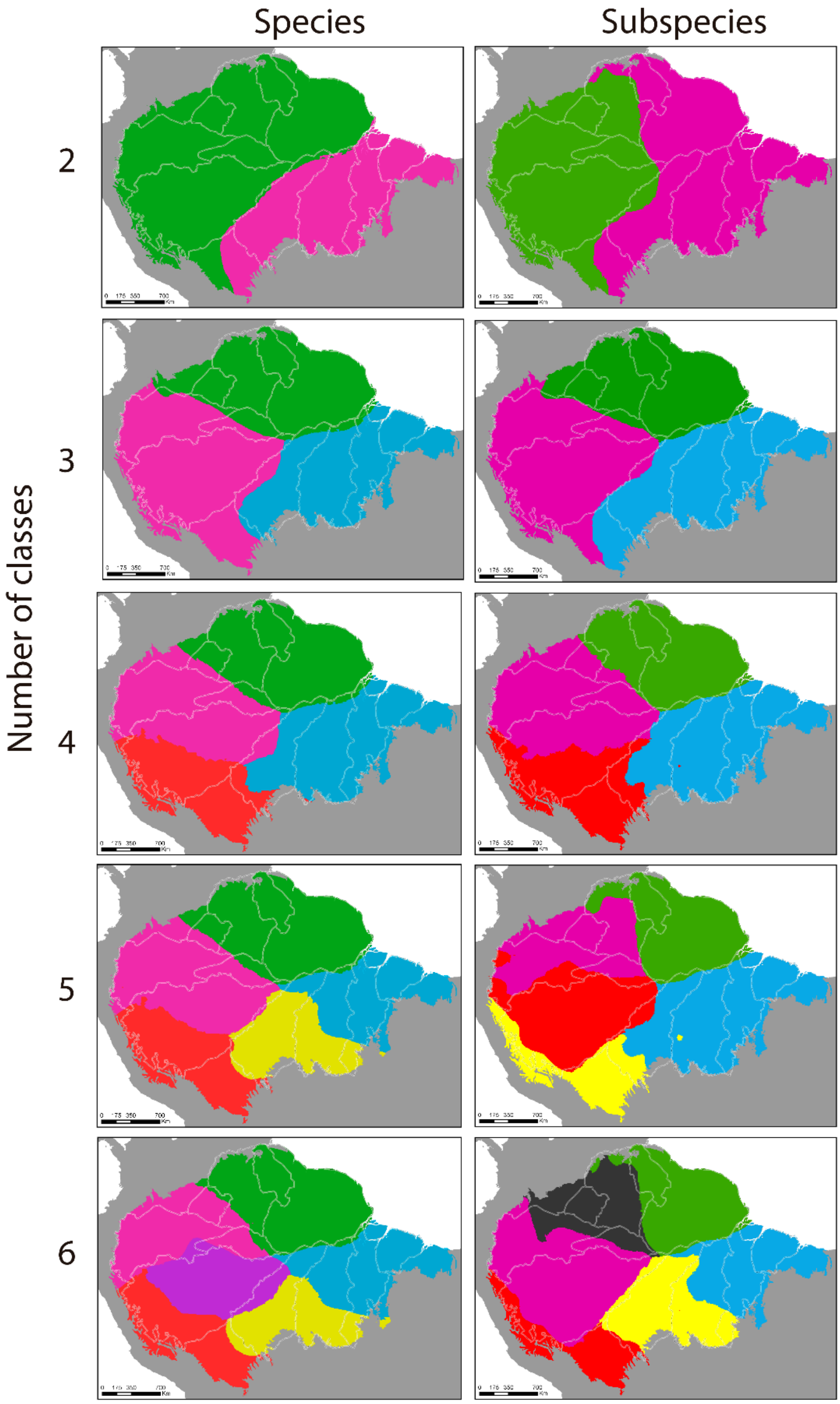

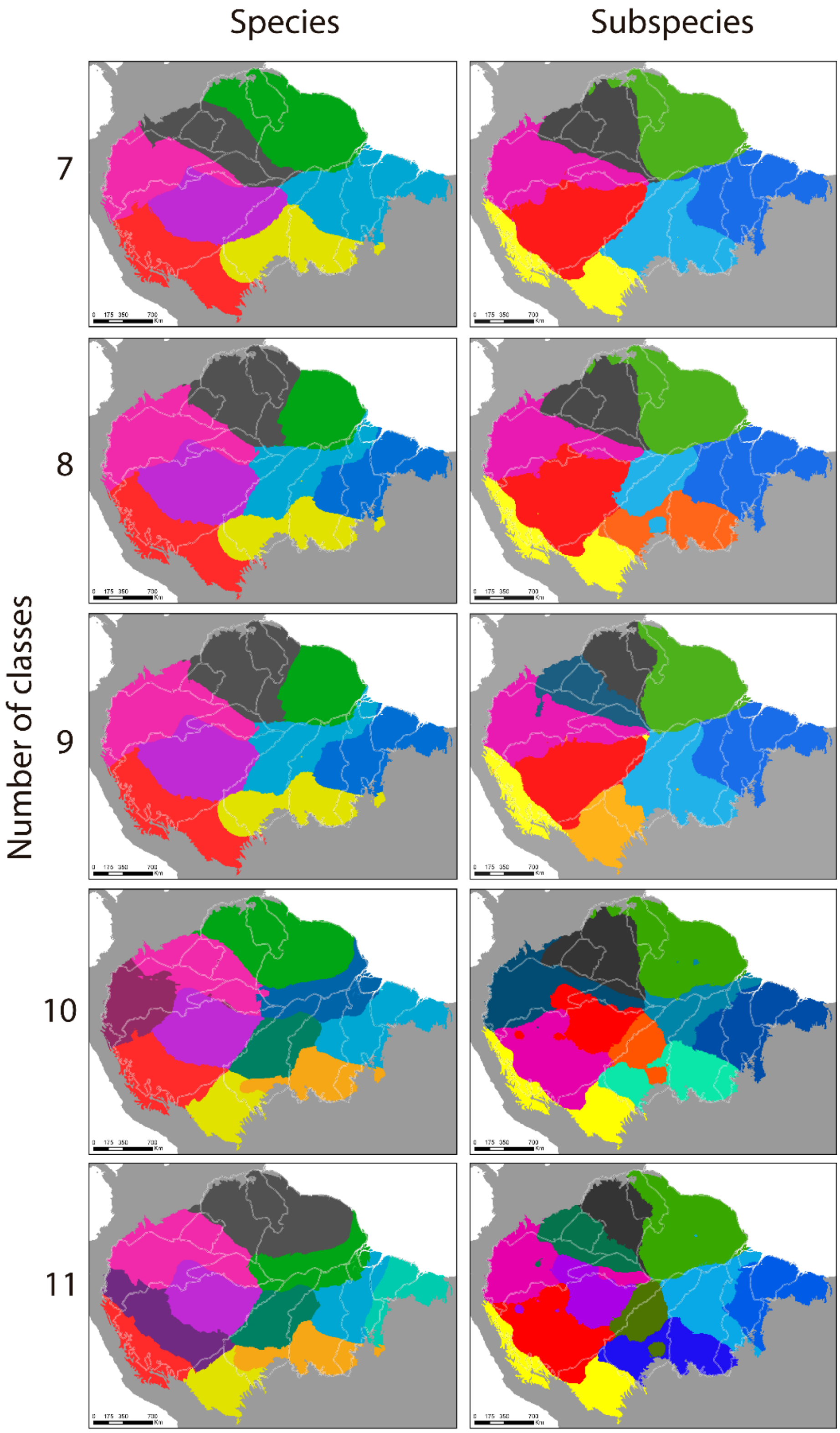
Species
Subspecies
Number of classes
7
8
9
10
11

21- Interpolation of the three axes of NMDS based on species and subspecies occurrence. Beta-diversity is partitioned into turnover and nestedness components

Map created in ArcGIS 10.1 (http://www.esri.com)

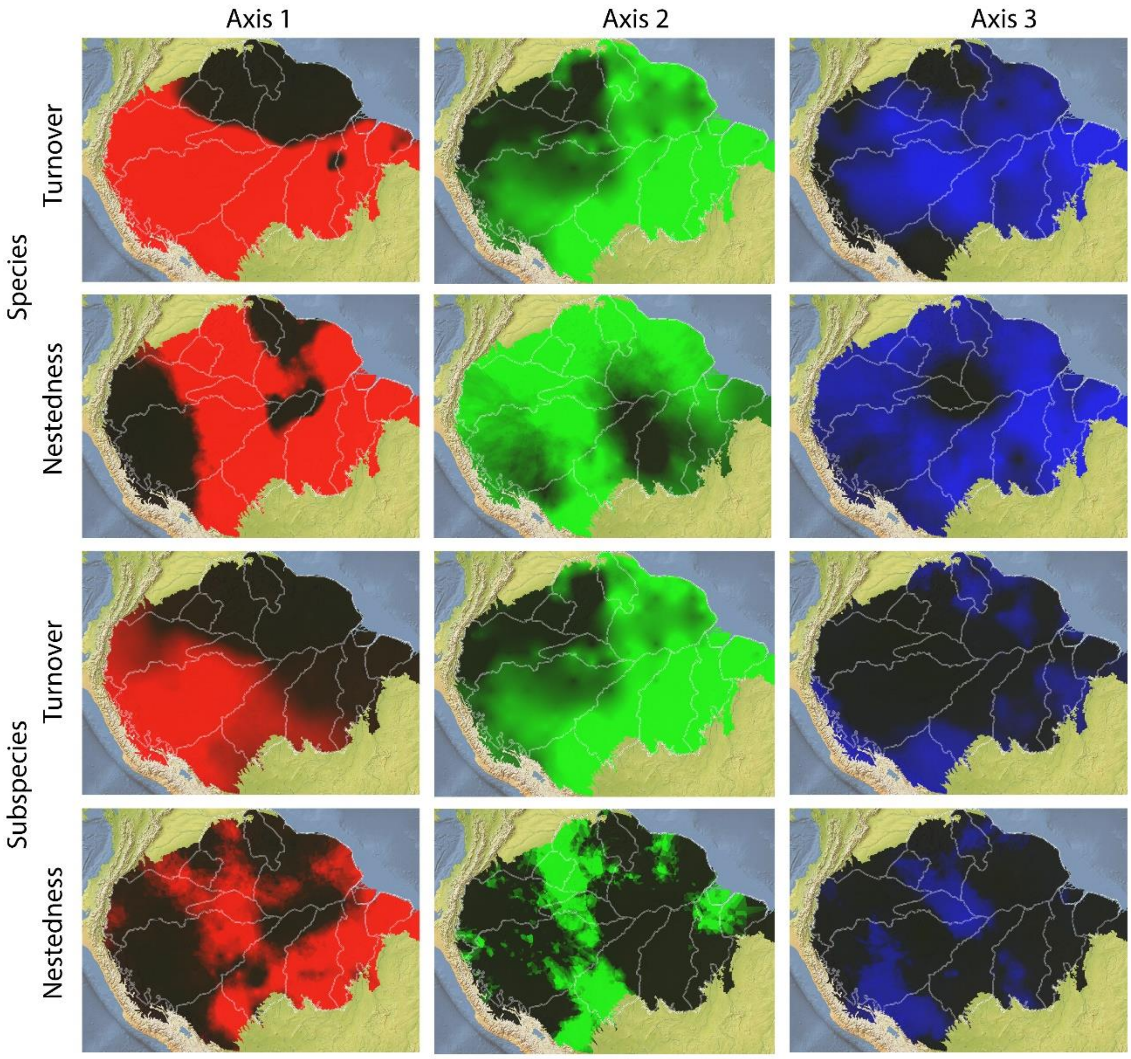

22- Results of Discriminant Analysis.

		Guiana	Imeri	Inambari	Napo	PantepuiDuida	PantepuiGranSabana	Belem	Jau	Rondonia	Tapajos	Xingu	Total
NMDS based species	PercentCorrect	86.87	66.00	44.00	61.00	0.00	0.00	54.08	37.00	72.16	15.00	59.00	45.42
	pvalue	0.09	0.09	0.09	0.09	0.09	0.09	0.09	0.09	0.09	0.09	0.09	
NMDS based in subspecies	PercentCorrect	88.88	99.93	89.00	59.00	65.21	88.88	97.93	39.00	83.87	58.58	75.25	76.76
	pvalue	0.09	0.09	0.09	0.09	0.08	0.09	0.09	0.09	0.08	0.09	0.09	
